# Dissection of FOXO1-Induced LYPLAL1-DT Impeding Triple-Negative Breast Cancer Progression via Mediating hnRNPK/β-Catenin Complex

**DOI:** 10.34133/research.0289

**Published:** 2023-12-15

**Authors:** Yuhui Tang, Wenwen Tian, Shaoquan Zheng, Yutian Zou, Jindong Xie, Junsheng Zhang, Xing Li, Yuying Sun, Jing Lan, Ning Li, Xiaoming Xie, Hailin Tang

**Affiliations:** ^1^ State Key Laboratory of Oncology in South China, Guangdong Provincial Clinical Research Center for Cancer, Sun Yat-sen University Cancer Center, Guangzhou 510060, P. R. China.; ^2^ Affiliated Cancer Hospital and Institute of Guangzhou Medical University, No.78 Hengzhigang Road, Guangzhou 510095, P. R. China.; ^3^ Breast Disease Center, The First Affiliated Hospital, Sun Yat-sen University, 58 Zhongshan Er Road, Guangzhou 510080, P. R. China.; ^4^ Department of General Surgery, The First Affiliated Hospital of Soochow University, Suzhou 215006, P. R. China.

## Abstract

Triple-negative breast cancer (TNBC) is considered as the most hazardous subtype of breast cancer owing to its accelerated progression, enormous metastatic potential, and refractoriness to standard treatments. Long noncoding RNAs (lncRNAs) are extremely intricate in tumorigenesis and cancerous metastasis. Nonetheless, their roles in the initiation and augmentation of TNBC remain elusive. Here, in silico analysis and validation experiments were utilized to analyze the expression pattern of clinically effective lncRNAs in TNBC, among which a protective lncRNA LYPLAL1-DT was essentially curbed in TNBC samples and indicated a favorable prognosis. Gain- and loss-of-function assays elucidated that LYPLAL1-DT considerably attenuated the proliferative and metastatic properties along with epithelial-mesenchymal transition of TNBC cells. Moreover, forkhead box O1 (FOXO1) was validated to modulate the transcription of LYPLAL1-DT. Mechanistically, LYPLAL1-DT impinged on the malignancy of TNBC mainly by restraining the aberrant reactivation of the Wnt/β-catenin signaling pathway, explicitly destabilizing and diminishing β-catenin protein by interacting with heterogeneous nuclear ribonucleoprotein K (hnRNPK) and constricting the formation of the hnRNPK/β-catenin complex. Conclusively, our present research revealed the anti-oncogenic effects of LYPLAL1-DT in TNBC, unraveling the molecular mechanisms of the FOXO1/LYPLAL1-DT/hnRNPK/β-catenin signaling axis, which shed innovative light on the potential curative medicine of TNBC.

## Introduction

Female breast cancer has currently outstripped lung cancer to be the leading cause of cancer morbidity in 2020 worldwide, in addition to the fifth core cause of global cancer mortality [1]. As the most aggressive subtype of breast cancer, triple-negative breast cancer (TNBC) constituted roughly 15 to 20% of patients with breast cancer and was defined to lack the expression of estrogen receptor (ER), progesterone receptor (PR), and human epidermal growth factor receptor type 2 (Her-2) markers [2]. Patients with TNBC might gain a relatively unfavorable prognosis due to its rapid aggressiveness, high rates of relapse, frequently emerged distant metastasis, and refractoriness to standard therapy [3,4]. Therefore, it is essentially crucial to plumb novel biomarkers of TNBC tumorigenesis and early metastasis for seeking the advanced treatment strategies.

Long noncoding RNAs (lncRNAs) are characterized by a vital class of transcripts exceeding 200 nucleotides and attaining limited or no protein-coding potential [5,6]. lncRNAs play an essential role in diverse biological processes, comprising embryonic development, homeostasis maintenance, and especially regulating cancerous progression [7]. A host of TNBC-implicated lncRNAs have been identified, and for instance, lncRNA LINK-A activates normoxic hypoxia-inducible factor α (HIF1α) signaling and correlates with an inferior prognosis in TNBC [8], in addition to our previously studied lncRNA HUMT and LINC01638, respectively, implying TNBC lymph node metastasis by recruiting YBX1 and maintaining the mesenchymal traits of TNBC cells by interacting with c-myc [9,10]. However, more clinically effective lncRNAs revealing the oncogenesis and metastasis of TNBC remain elusive and call for more explorations.

Wnt signaling manipulates a stack of developmental process, comprising cell growth, differentiation, migration, genetic stability, epithelial-mesenchymal transition, stem cell renewal, and so on [11,12]. Described as the canonical Wnt pathway, the Wnt/β-catenin signaling pathway is mainly dependent by the stabilized β-catenin accumulations in the cytoplasm and translocation in the nucleus to form the transcriptional complex by binding the transcription factors (TFs) T cell factor/lymphoid enhancer factor (TCF/LEF) and activating the downstream oncogenic genes [13]. Emerging evidences illustrate that lncRNAs take partly pivotal effects in modulating the Wnt/β-catenin signaling pathway in diverse cancers [14]. Nevertheless, the underlying mechanisms of Wnt/β-catenin signaling-related lncRNAs in TNBC require more dissections.

Here, we identified a novel tumor-suppressed lncRNA LYPLAL1-DT (lysophospholipase like 1 divergent transcript) in TNBC, which indicated a favorable prognosis and curbed the proliferative and metastatic abilities of TNBC cells whether in vitro or in vivo. Additional mechanistic assays indicated that LYPLAL1-DT was transcriptionally regulated by forkhead box O1 (FOXO1) and destabilized β-catenin protein by means of interacting with heterogeneous nuclear ribonucleoprotein K (hnRNPK) and subsequently attenuating the formation of the hnRNPK/β-catenin complex. Accordingly, our data shed light on that LYPLAL1-DT served as a novel protective biomarker and turned to be a promising curative target for the initiation and metastasis of TNBC.

## Results

### LYPLAL1-DT expression is impeded in TNBC samples and indicates a favorable prognosis

**Fig. 1.**
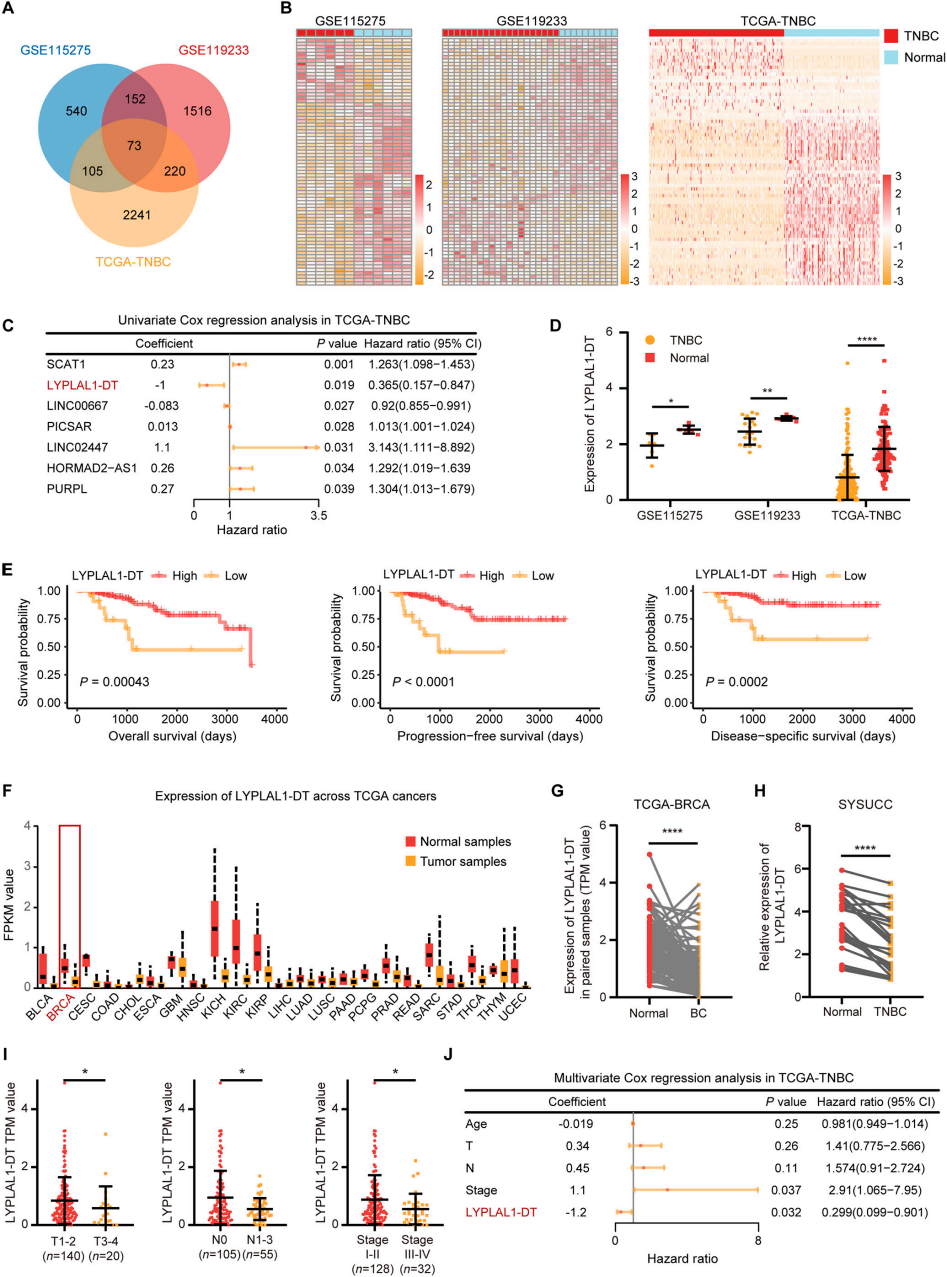
LYPLAL1-DT expression is curbed in TNBC samples and correlates with a favorable prognosis. (A) Venn diagram depicting the identification of 73 common differentially expressed lncRNAs from GSE115275, GSE119233, and TCGA-TNBC cohorts. (B) Heatmaps showing the expression patterns of 73 candidate lncRNAs in three datasets. (C) Univariate Cox regression analysis based on 73 eligible lncRNAs in TCGA-TNBC dataset. (D) Expression level of LYPLAL1-DT in TNBC and nontumor normal samples of three datasets. (E) Kaplan–Meier curves displaying TNBC patients with high expression of LYPLAL1-DT gain favorable overall survival, progression-free survival, and disease-specific survival. (F) Expression level of LYPLAL1-DT in tumor and nontumor normal samples across various cancer types in the TCGA cohort, including breast cancer (BRCA). (G) Expression level of LYPLAL1-DT in 120 matched breast cancer (BC) and noncancerous normal samples of the TCGA-BRCA cohort. (H) Relative expression level of LYPLAL1-DT in 24 pairs of TNBC and matched nontumor tissues of the SYSUCC cohort using RT-qPCR. (I) Expression level of LYPLAL1-DT in TNBC patients with various T stage, N stage, or AJCC stage from the TCGA-TNBC cohort. (J) Multivariate Cox regression analysis incorporating age, T stage, N stage, AJCC stage, and LYPLAL1-DT. Error bars represent mean ± SD. *P < 0.05, **P < 0.01, ****P < 0.0001.

To investigate the lncRNA pivotal to TNBC progression, the substantial differentially expressed lncRNAs were uncovered between TNBC and nontumoral samples in GSE115275, GSE119233, and The Cancer Genome Atlas database (TCGA)-TNBC cohorts (Fig. S1A) and 73 differentially expressed lncRNAs were screened out after intersecting the results (Fig. 1A), the expression patterns of which were displayed in the heatmaps (Fig. 1B). Furthermore, seven clinically vital lncRNAs were obtained after performing univariate Cox regression analysis for overall survival (OS) based on the expression of 73 candidate lncRNAs above in TCGA-TNBC dataset (Fig. 1C). Subsequently, scrutinizing the significance and uniformity of the expression (Fig. S1B) and prediction to OS, progression-free survival (PFS), and disease-specific survival (DSS) of TNBC patients (Fig. S2A to F), LYPLAL1-DT was selected as the focus of the present study. LYPLAL1-DT (Ensembl ID: ENSG00000228063) was the divergent transcript of the gene encoding lysophospholipase like 1 (LYPLAL1) and located at chromosome 1q41 (Fig. S4C). Ulteriorly, LYPLAL1-DT expression was remarkably impeded in TNBC samples compared to the nontumoral samples in three cohorts (Fig. 1D) and high expression of LYPLAL1-DT was related to a favorable OS, PFS, and DSS in the TCGA-TNBC cohort (Fig. 1E). Similar to transcriptional down-regulation in breast cancer, LYPLAL1-DT was universally decreased in various cancer samples in comparison with noncancerous normal samples (Fig. 1F). Furthermore, LYPLAL1-DT expression was considerably diminished in 120 breast cancer samples compared to the paired nontumoral samples in the TCGA-BRCA cohort (Fig. 1G), which was verified in 24 pairs of para-cancerous breast and TNBC tissues by real-time quantitative polymerase chain reaction (RT-qPCR) in the Sun Yat-sen University Cancer Center (SYSUCC) cohort (Fig. 1H). To further investigate the importance of LYPLAL1-DT in clinical practice, the relevance of LYPLAL1-DT and several clinicopathologic parameters containing T stage, N stage, and American Joint Committee on Cancer (AJCC) stage was uncovered by comparing LYPLAL1-DT expression between TNBC groups with more alleviated or more aggressive progression in the TCGA-TNBC cohort. The results deciphered that low expression of LYPLAL1-DT represented higher T stage, N stage, and AJCC stage (Fig. 1I). In addition, multivariate Cox regression analysis incorporating age, N and T stage, AJCC stage, and LYPLAL1-DT was performed, indicating that AJCC stage and LYPLAL1-DT conserved as essential independent predictors for TNBC prognosis (Fig. 1J).

In order to further confirm whether LYPLAL1-DT played the same role in non-TNBC subtypes, we executed Kaplan–Meier survival analyses of OS, PFS, and DSS using LYPLAL1-DT expression in the non-TNBC cohort (*n* = 789) of the TCGA database, which verified that LYPLAL1-DT expression was not correlated to the OS, PFS, and DSS in the non-TNBC cohort (Fig. S3A). Additionally, univariate Cox regression analysis for OS based on LYPLAL1-DT expression in the TCGA–non-TNBC cohort unveiled that LYPLAL1-DT was not an independent prognostic predictor for non-TNBC patients (Fig. S3B). Afterward, LYPLAL1-DT expression was compared in non-TNBC patients with different grades of T stage, N stage, or AJCC stage, which hinted that LYPLAL1-DT exerted negligible effect to predict a favorable or worse clinicopathologic grade for non-TNBC patients (Fig. S3C). In a nutshell, LYPLAL1-DT indeed served as a critical clinical predictor for TNBC than for non-TNBC.

To evaluate the coding probability of LYPLAL1-DT, National Center for Biotechnology Information (NCBI) ORFfinder was first used and no typical open reading frame (ORF) for protein coding that exceeded 300 nucleotides was detected on the plus-strand sequence of LYPLAL1-DT (Fig. S4A and B), which indicated that there is an extremely low possibility for coding potential of LYPLAL1-DT. Additionally, the PhyloCSF model was utilized to validate the result above and the negative PhyloCSF value calculated along the entire sequence of LYPLAL1-DT suggested a low coding capability (Fig. S4C). Moreover, CPAT and CPC2 web tools were further employed to discover that LYPLAL1-DT has a low coding probability similar to the previous typical lncRNAs, like NEAT1, XIST, LINK-A, and HOTAIR [8,15–17] (Fig. S4D). Taken together, the results above confirmed that LYPLAL1-DT had no coding potential and conserved as a novel lncRNA.

### LYPLAL1-DT is transcriptionally regulated by FOXO1

**Fig. 2.**
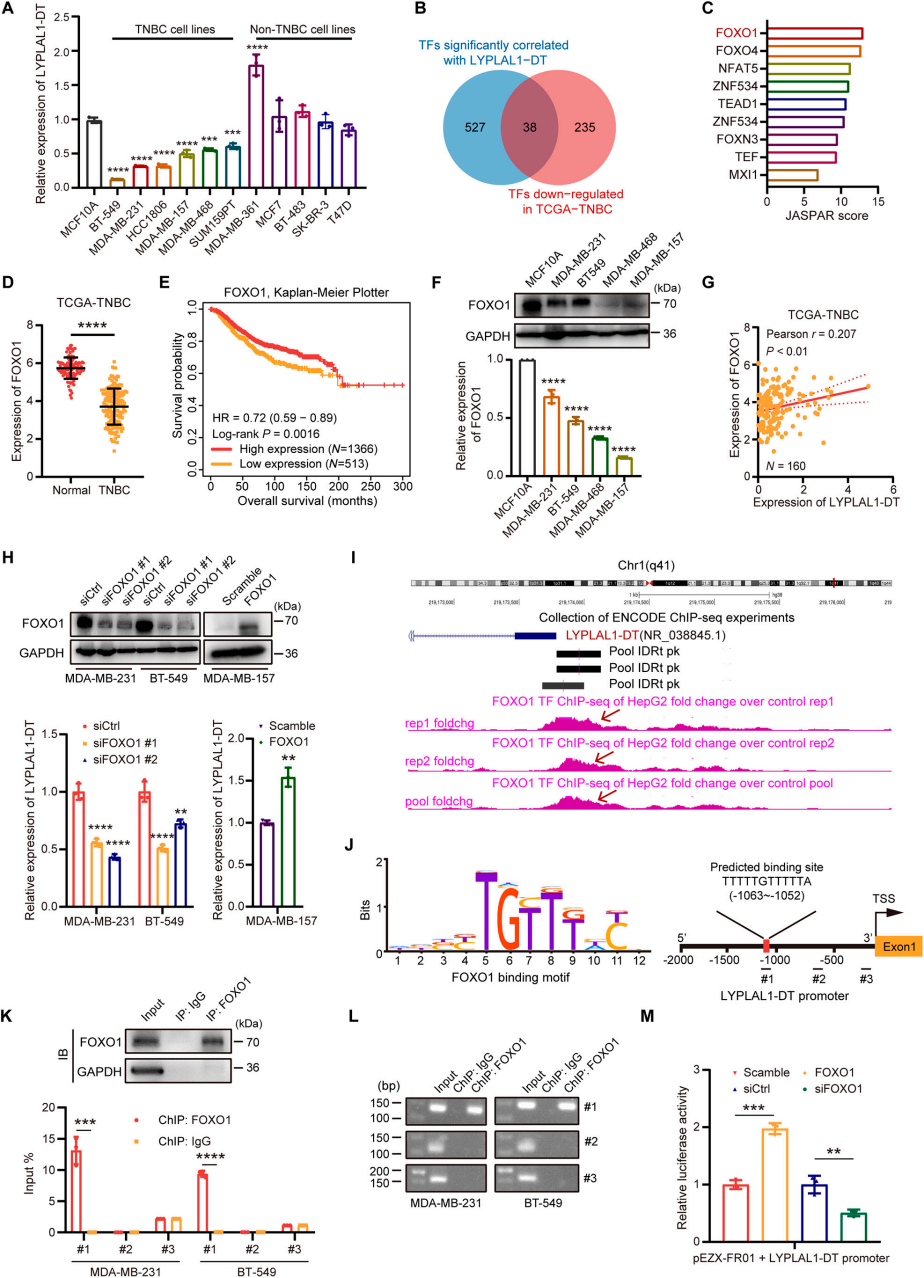
LYPLAL1-DT is transcriptionally modulated by FOXO1.(A) Relative expression level of LYPLAL1-DT in TNBC cell lines, non-TNBC cell lines, and MCF10A. (B) Venn diagram delineating the identification of potential transcription factors (TFs) regulating LYPLAL1-DT. (C) Binding probability between the eligible TFs and the promoter of LYPLAL1-DT calculated in the JASPAR database. (D) Expression level of FOXO1 in nontumor normal samples and TNBC samples in the TCGA-TNBC cohort. (E) Kaplan–Meier curve showing that breast cancer patients with low expression of FOXO1 attain an inferior overall survival. (F) Expression level of FOXO1 protein (top) and FOXO1 RNA (bottom) in TNBC cell lines and MCF10A. (G) Significant positive correlation between LYPLAL1-DT and FOXO1 expression levels in the TCGA-TNBC cohort. (H) Validation of silencing FOXO1 in MDA-MB-231 and BT-549 cells and overexpressing FOXO1 in MDA-MB-157 cells (top), in addition with the corresponding expression alterations of LYPLAL1-DT (bottom). (I) ChIP-seq from the ENCODE database verifying FOXO1 binding peaks on the promoter region of LYPLAL1-DT in HepG2 cells. (J) The binding motif of FOXO1 (left) and the predicted binding site of FOXO1 on the promoter region of LYPLAL1-DT as well as the designed fragments for the further ChIP analysis (right). (K) Validation of FOXO1 antibody utilized in IP assay (top) and RT-qPCR result for ChIP assay using antibodies against FOXO1 and IgG (bottom). (L) Confirmation of RT-qPCR products in ChIP assay using nuclear acid electrophoresis. (M) Promoter dual-luciferase reporter assay conducted by cotransfections of plasmid pEZX-FR01 inserting LYPLAL1-DT promoter and plasmid overexpressing FOXO1 (FOXO1), or siRNA knocking down FOXO1 (siFOXO1), or the corresponding control plasmid (Scramble) or nontarget siRNA (siCtrl), which unraveled that FOXO1 definitely bound with the promoter of LYPLAL1-DT. Error bars represent mean ± SD. *P < 0.05, **P < 0.01, ***P < 0.001, ****P < 0.0001.

Expression patterns of LYPLAL1-DT in a nontumoral mammary epithelial cell line MCF10A and a number of breast cancer cell lines were explored using RT-qPCR, and the result disclosed that LYPLAL1-DT expression was noticeably reduced in a host of TNBC cell lines, especially BT-549 and MDA-MB-231, comparing to that in MCF10A and a series of non-TNBC cell lines (Fig. 2A), which was consistent with the results in nontumoral and TNBC samples and tissues. It is widely acknowledged that TFs are essentially crucial to regulate RNA expression including lncRNA. Hence, to further investigate the upstream TF controlling LYPLAL1-DT, we initially identified the TFs considerably correlated with LYPLAL1-DT when setting the significant criterion to *P* < 0.05 and Pearson *r* > 0.2 (Table S4) and the TFs down-regulated in the TCGA-TNBC cohort (Table S5). After intersecting with the results above, 38 potential TFs were acquired (Fig. 2B) and the JASPAR database was exploited to figure out the probability of these eligible TFs in binding the promoter of LYPLAL1-DT (Table S6), which illustrated that FOXO1 could be the most likely to contribute to regulating the expression of LYPLAL1-DT (Fig. 2C). Furthermore, FOXO1 expression was detected to considerably decline in TNBC samples of the TCGA-TNBC cohort compared to nontumor normal breast samples (Fig. 2D) and the same tendency was found in 14 pairs of TNBC samples and normal breast samples (Fig. S5A). The lower expression of FOXO1 implied an inferior OS for breast cancer patients (Fig. 2E), as well as a shortened recurrence-free survival (RFS) and distant metastasis-free survival (DMFS) (Fig. S5B). In addition, FOXO1 expression was validated in MCF10A and several TNBC cell lines by Western blot and RT-qPCR, which demonstrated that the expression of FOXO1 was dramatically slashed in TNBC cell lines contrasting that in MCF10A (Fig. 2F). Likewise, the expression levels of FOXO1 in another nontumoral mammary epithelial cell line HMEL and a multitude of breast cancer cell lines were presented in Fig. S5C, with regard to the Cancer Cell Line Encyclopedia (CCLE) database, which hinted that in comparison with HMEL, FOXO1 expression was diminished in an ocean of TNBC cell lines, except SUM159PT and SUM149PT.

Besides, the positive correlation of LYPLAL1-DT expression and FOXO1 expression in the TCGA-TNBC cohort was shown in Fig. 2G and subsequently was validated in TNBC cell lines using small interfering RNAs (siRNAs) targeting FOXO1 (termed siFOXO1#1 and #2) and plasmid pcDNA-3.1 overexpressing FOXO1 (termed FOXO1), which found that silencing FOXO1 or overexpressing FOXO1 indeed induced LYPLAL1-DT down-regulation or up-regulation (Fig. 2H and Fig. S5D). The chromatin immunoprecipitation sequencing (ChIP-seq) result of FOXO1 protein from the ENCODE database visualized in the University of California Santa Cruz (UCSC) Genome database hinted that FOXO1-binding pool could peak in about 1,000 base pairs (bp) upstream from the transcription start site (TSS) of LYPLAL1-DT (Fig. 2I). According to the binding motif of FOXO1 originated from the HOCOMOCO database [18], the binding site of FOXO1 on the LYPLAL1-DT promoter region was calculated and the specific primers for various promoter segments of LYPLAL1-DT were synthesized for further analysis (Fig. 2J). To confirm which site(s) FOXO1 combined with LYPLAL1-DT promoter, ChIP was applied in MDA-MB-231 and BT549, and the outcome of the following RT-qPCR disclosed that FOXO1 protein bound the fragment −1,115 to −971 bp upstream from TSS of LYPLAL1-DT (site #1) rather than other fragments (Fig. 2K). The products of RT-qPCR were further validated using nuclear acid electrophoresis (Fig. 2L). Ultimately, the promoter dual-luciferase reporter assay was conducted to reconfirm the combination of FOXO1 and the promoter region of LYPLAL1-DT, which unveiled that the luciferase activity was dramatically promoted in the group cotransfected FOXO1 and plasmid pEZX-FR01 inserting LYPLAL1-DT promoter than the control group, whereas the luciferase activity was obviously decreased in the group cotransfected siFOXO1 #1 and plasmid pEZX-FR01 containing LYPLAL1-DT promoter than the siCtrl group (Fig. 2M). In summary, these results elucidated that the transcriptional expression of LYPLAL1-DT is actually mediated by FOXO1.

### LYPLAL1-DT restricts TNBC proliferation, metastasis and EMT in vitro

**Fig. 3.**
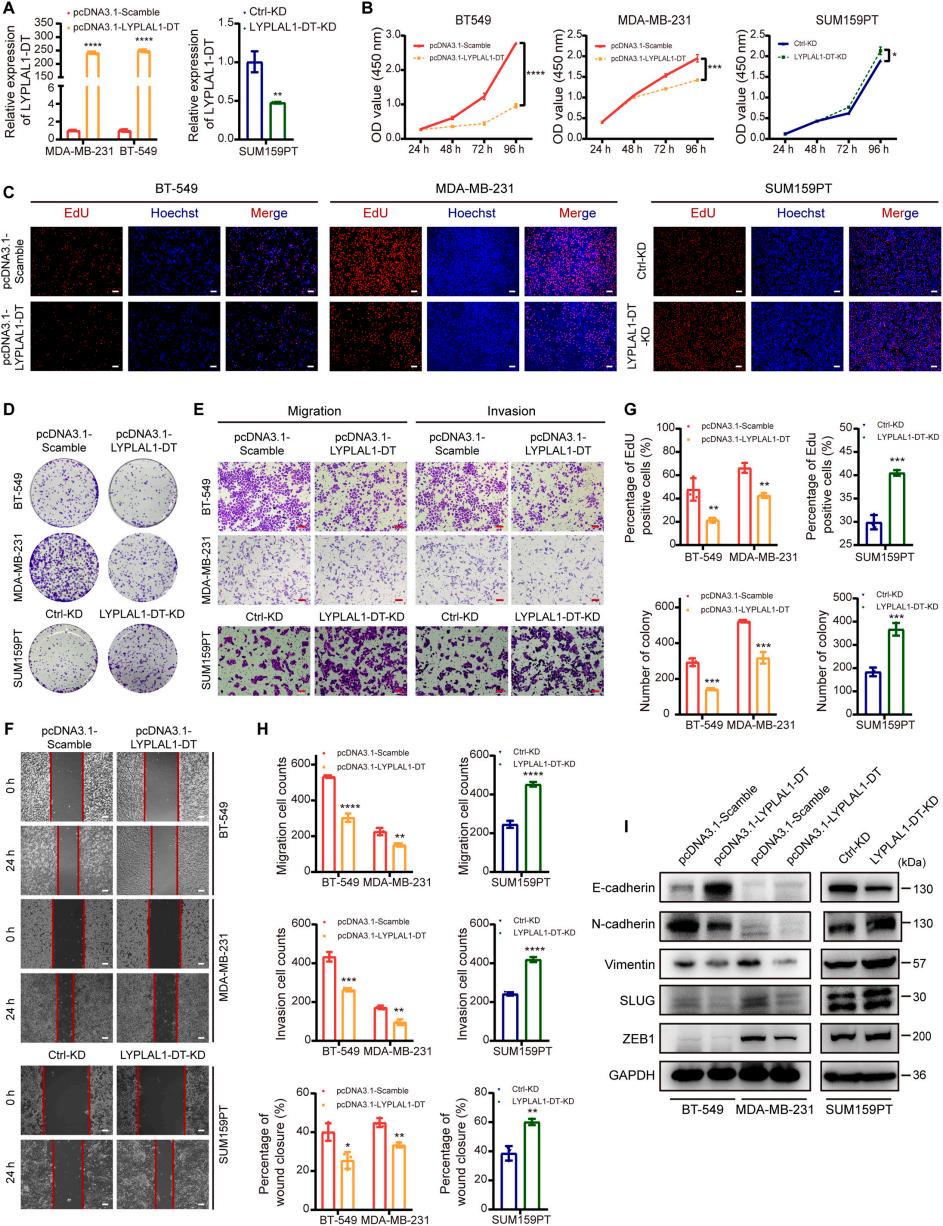
LYPLAL1-DT exerts antitumorigenic and antimetastatic effects to TNBC cells in vitro. (A) RT-qPCR result validating effects of overexpressing LYPLAL1-DT in MDA-MB-231 and BT-549 cells and knocking down LYPLAL1-DT in SUM159PT cells. (B) CCK-8 proliferation assays manifesting that overexpressing LYPLAL1-DT limited the proliferation of TNBC cells, while silencing LYPLAL1-DT promoted the proliferative nature. (C to F) Representative graphs of EdU staining proliferation assays (C), colony formation assays (D), transwell assays (E), and wound-healing assays (F) after performing overexpression or knockdown of LYPLAL1-DT in TNBC cells. (G) Quantitative data of EdU staining proliferation assays (top) and colony formation assays (bottom). (H) Quantitative data of transwell assays evaluating migration (top) or invasion (medium) properties and wound-healing assays (bottom). (I) Markers of epithelial-mesenchymal transition (EMT) were investigated in condition of overexpressing or knocking down LYPLAL1-DT in TNBC cells. Error bars represent mean ± SD. Scale bars in this figure all signify 100 μm. *P < 0.05, **P < 0.01, ***P < 0.001, ****P < 0.0001.

To substantiate the function of LYPLAL1-DT and its impact on TNBC progression, functional experiments were performed after LYPLAL1-DT was exogenously overexpressed in BT-549 as well as MDA-MB-231 cells and knocked down in SUM159PT cells (Fig. 3A). Cell counting kit-8 (CCK-8) proliferation assays and 5-ethynyl-2' -deoxyuridine (EdU) staining assays implied that enhancing LYPLAL1-DT expression could suppress the proliferation of TNBC cells, while knocking down LYPLAL1-DT induced the opposite effects (Fig. 3B, C, and G). Likewise, colony formation assays illustrated that increasing LYPLAL1-DT expression could restrain the ability of colony formation in both TNBC cells, whereas decreasing LYPLAL1-DT had a boosting impact (Fig. 3D and G). To further explore the impact of LYPLAL1-DT on the metastasis of TNBC cells, transwell assays and wound-healing assays were operated, which figured out that overexpression of LYPLAL1-DT could restrict the natures of migration and invasion in BT-549 and MDA-MB-231 cells, whereas silencing LYPLAL1-DT could augment the mobility of SUM159PT cells (Fig. 3E, F, and H). It is universally known that aberrant reactivation of epithelial-mesenchymal transition (EMT) is correlated to malignant properties of tumor cells during cancer progression and metastasis [19], and thus, markers of EMT were investigated, which showed that improving LYPLAL1-DT expression enabled to curb EMT of TNBC cells and repressing LYPLAL1-DT expression had a contrary effect to EMT of TNBC cells (Fig. 3I and Fig. S5E). Collectively, these in vitro results unraveled that overexpression of LYPLAL1-DT limits proliferation, migration, invasion, and EMT of TNBC cells in vitro.

### LYPLAL1-DT obstructs tumor growth and lung metastasis of TNBC cells in vivo

**Fig. 4.**
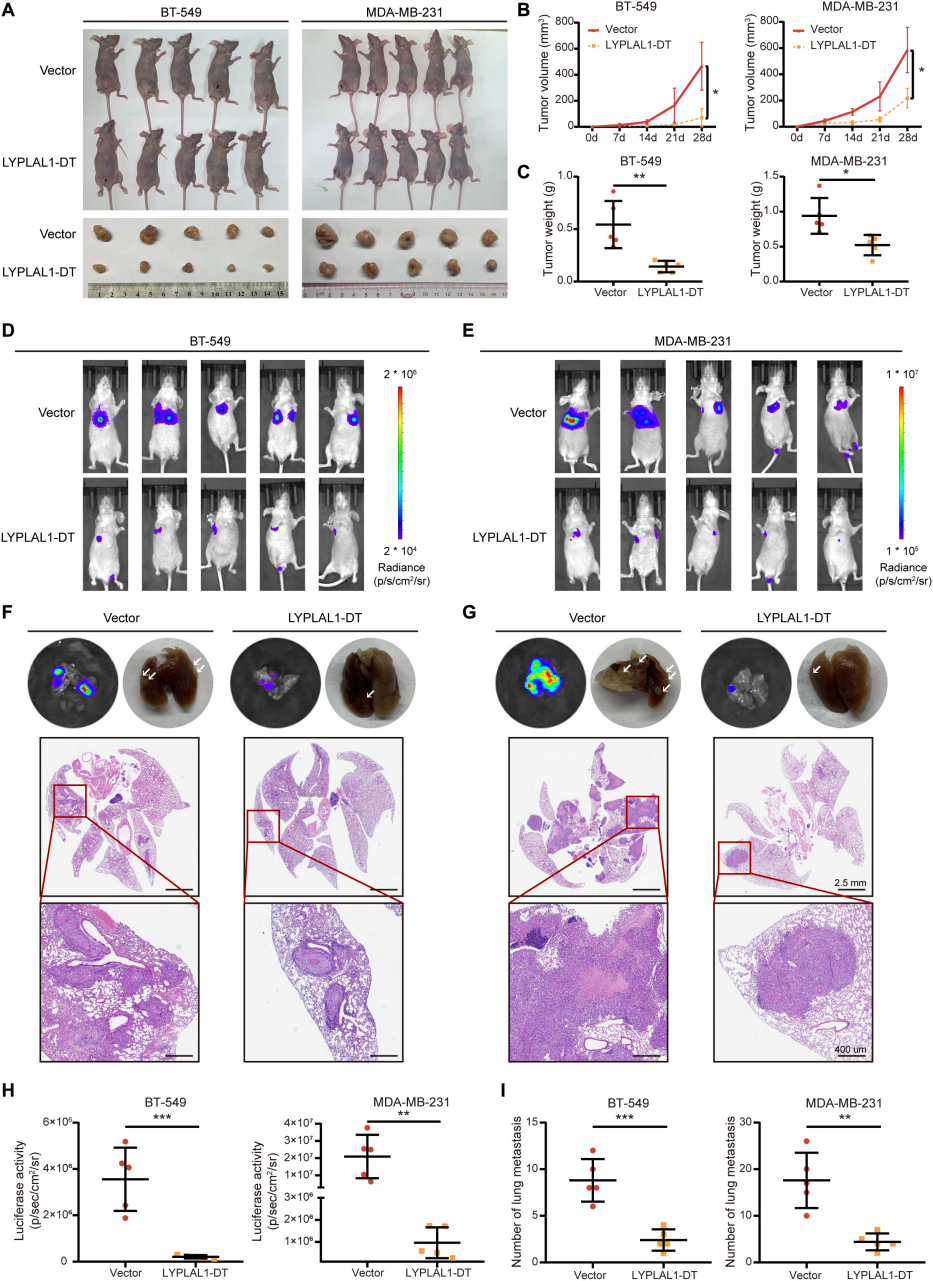
LYPLAL1-DT obstructs tumor growth and lung metastasis of TNBC cells in vivo. (A to C) Representative images of xenograft tumor morphology (A), quantitative data of tumor growth (B), and tumor weights of xenograft tumors (C) in nude mice (five mice per group) injected subcutaneously into the mammary fat pad using the corresponding stably transfected TNBC cells. (D to I) Representative images of optical luciferase imaging assays in vivo (D and E) and lung metastatic morphology and H&E staining in lung metastatic foci (F and G), as well as quantitative data of optical luciferase imaging assays (H) and metastatic foci in lung tissue (I) in nude mice (five mice per group) injected intravenously via the tail vein using the corresponding stably transfected TNBC cells. Error bars represent mean ± SD. *P < 0.05, **P < 0.01, ***P < 0.001, ****P < 0.0001.

The influence of LYPLAL1-DT on the tumor growth and metastasis of TNBC cells in vivo was further ascertained by models of xenograft tumor and lung metastasis established in nude mice using BT-549 and MDA-MB-231 cells stably overexpressing LYPLAL1DT (LYPLAL1-DT group) or stably transfecting the empty vector (Vector group). The results of xenograft tumor models presented that the tumors were visibly smaller and gained slower growth and more limited weights in the LYPLAL1-DT groups than in the Vector groups (Fig. 4A to C). Additionally, lung metastasis models in vivo suggested that the number of lung metastatic nodules was strikingly reduced in the LYPLAL1-DT group of both two TNBC cells contrasting the control group and was validated using hematoxylin and eosin (H&E) staining assay (Fig. 4D to I). Summarily, the aforementioned results corresponded with in vivo results, which clarified that LYPLAL1-DT obstructs TNBC tumorigenesis and lung metastasis.

### LYPLAL1-DT hinders Wnt/β-catenin pathway by destabilizing β-catenin protein

**Fig. 5.**
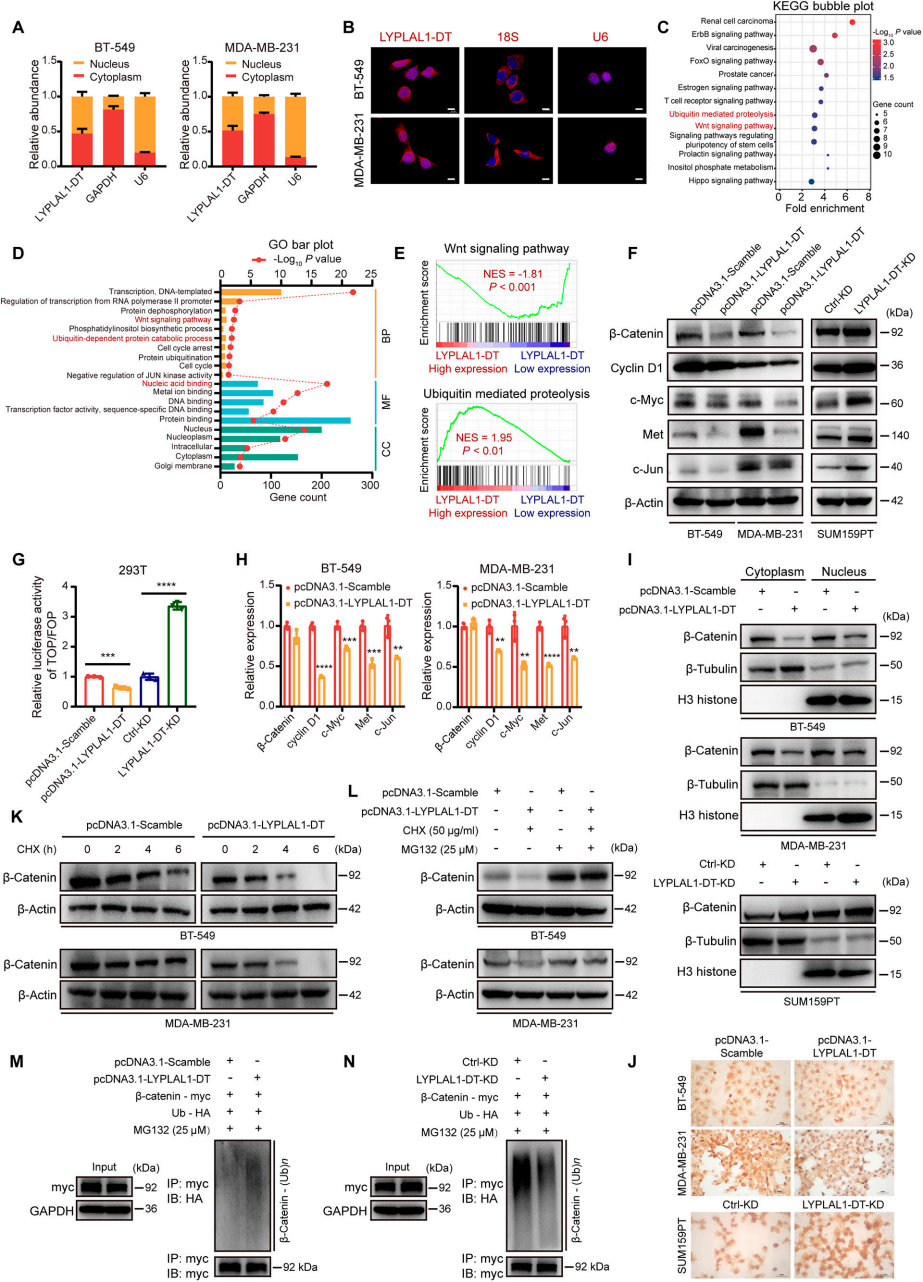
LYPLAL1-DT inhibits the Wnt/β-catenin pathway by enhancing ubiquitination of β-catenin and thereby destabilizing its protein. (A) Subcellular fractionation assays showing the relative abundance of LYPLAL1-DT in the cytoplasm and nucleus of TNBC cells. GAPDH and U6 were applied as positive controls for the cytoplasmic and nuclear fractions, respectively. (B) RNA-FISH depicting the translocation of LYPLAL1-DT in the cytoplasm and nucleus of TNBC cells (scale bar, 20 μm). 18S and U6 were applied as positive controls for the cytoplasmic and nuclear fractions, respectively. (C and D) Functional enrichment assays using KEGG pathways (C) and GO pathways (D) based on the coding genes highly correlated to LYPLAL1-DT. (E) GSEA analysis illustrating that the expression of LYPLAL1-DT was negatively related to the Wnt signaling pathway but positively associated with ubiquitin-mediated proteolysis. (F) Expression levels of β-catenin and its downstream oncogenic genes were detected in TNBC cells ectopically overexpressing or silencing LYPLAL1-DT. (G) Results of TOP/FOP-flash luciferase assays indicating the transcriptional activity of β-catenin after increasing or decreasing LYPLAL1-DT expression. (H) RT-qPCR results confirming the relative RNA alterations of β-catenin and its downstream oncogenic genes when overexpressing LYPLAL1-DT. (I) Subcellular expression of β-catenin protein in the cytoplasm and nucleus was assessed after up-regulating or down-regulating LYPLAL1-DT in TNBC cells. β-Tubulin and H3 histone were administrated as positive controls for the nuclear and cytoplasmic fractions, respectively. (J) Immunocytochemistry staining showing β-catenin accumulation in the nucleus after overexpressing or knocking down LYPLAL1-DT in TNBC cells (scale bar, 50 μm). (K) CHX-chase assay performed in TNBC cells overexpressing LYPLAL1-DT and meanwhile treated with CHX (50 μg/ml) at 0, 2, 4, and 6 h to observe β-catenin expression. (L) Western blot showing the expression of β-catenin in LYPLAL1-DT-overexpressed or control TNBC cells treated with or without CHX and MG132 (25 μM) at 6 h. (M and N) Ubiquitination of β-catenin was investigated in input samples (left) and IP samples (right). Error bars represent mean ± SD. **P < 0.01, ***P < 0.001, ****P < 0.0001.

To elucidate the underlying mechanism of how LYPLAL1-DT mediated TNBC progression and metastasis, the subcellular localization of LYPLAL1-DT was initially detected, which was considered as the primary determinant of the molecular function of lncRNA [20]. The subcellular fractionation assays and RNA-FISH (fluorescence in situ hybridization) experiment demonstrated that LYPLAL1-DT was evenly located in the cytoplasm and the nucleus of BT-549 and MDA-MB-231 cells (Fig. 5A and B and Fig. S6A). Thereafter, to explore the biological pathways LYPLAL1-DT might get involved in, the notable genes highly correlated to LYPLAL1-DT were attained using Spearman correlation test in TCGA-TNBC matrix as the significant criterion was determined to |Spearman *ρ*| > 0.4 and *P* < 0.05 (Table S7), and then functional enrichment analyses were conducted based on these LYPLAL1-DT-related genes. Kyoto Encyclopedia of Genes and Genomes (KEGG) and Gene Ontology (GO) pathways enrichment analyses connoted that LYPLAL1-DT was likely to function in the Wnt signaling pathway and ubiquitin-mediated proteolysis (Fig. 5C and D), and Gene Set Enrichment Analysis (GSEA) analysis further denoted that the expression of LYPLAL1-DT was negatively linked to the Wnt signaling pathway but positively associated with ubiquitin-mediated proteolysis (Fig. 5E).

The canonical Wnt signaling pathway takes effects through β-catenin-dependent activation of downstream genes in a host of developmental processes, including cell growth, migration, and self-renewal of stem cells [11]. Therefore, Western blotting analysis was exploited to verify that ectopic overexpression of LYPLAL1-DT substantially dwindled β-catenin protein and its downstream target gene proteins consisting of CCND1 (cyclin D1), MYC (c-Myc), MET, and JUN (c-Jun) in TNBC cells, while silencing LYPLAL1-DT essentially activated β-catenin and the downstream genes (Fig. 5F and Fig. S6B). Furthermore, the outcomes of TOP/FOP-flash luciferase reporter analysis and RT-qPCR confirmed that LYPLAL1-DT inhibited β-catenin-dependent signaling in TNBC cells but not mediated the transcription of β-catenin (Fig. 5G and H). The impact of LYPLAL1-DT on the subcellular distribution of β-catenin was identified using Western blotting analysis, which discovered that overexpressed LYPLAL1-DT diminished the β-catenin protein in the cytoplasm and the nucleus of TNBC cells, whereas the opposite results were obtained by knocking down LYPLAL1-DT (Fig. 5I). Additionally, immunocytochemistry (ICC) analyses were executed in TNBC cells, which indicated that overexpressing LYPLAL1-DT reduced the accumulation of β-catenin in the nucleus, while LYPLAL1-DT knockdown increased that conversely (Fig. 5J and Fig. S6C). Since LYPLAL1-DT was detected to modulate the protein level of β-catenin but not RNA level, the hypothesis was formulated that LYPLAL1-DT might affect posttranscriptional modification of β-catenin, like ubiquitylation. Therefore, cycloheximide (CHX)-chase assay and rescue assay using MG132 were executed to reveal that LYPLAL1-DT could destabilize β-catenin protein and facilitate the degradation of β-catenin (Fig. 5K and L). Additionally, ubiquitination assay indicated that ubiquitination of β-catenin was raised in the presence of LYPLAL1-DT (Fig. 5M and Fig. S6D and F), and on the contrary, ubiquitination of β-catenin was weakened in the absence of LYPLAL1-DT (Fig. 5N and Fig. S6E and F), which explained the accelerating degradation of β-catenin protein. However, RNA immunoprecipitation (RIP) assay signified that LYPLAL1-DT attained no direct interaction with β-catenin protein (Fig. S6G). To summarize, LYPLAL1-DT regulates Wnt/β-catenin signaling by enhancing ubiquitination of β-catenin and destabilizing the protein.

### LYPLAL1-DT modulated β-catenin protein by interacting with hnRNPK

**Fig. 6.**
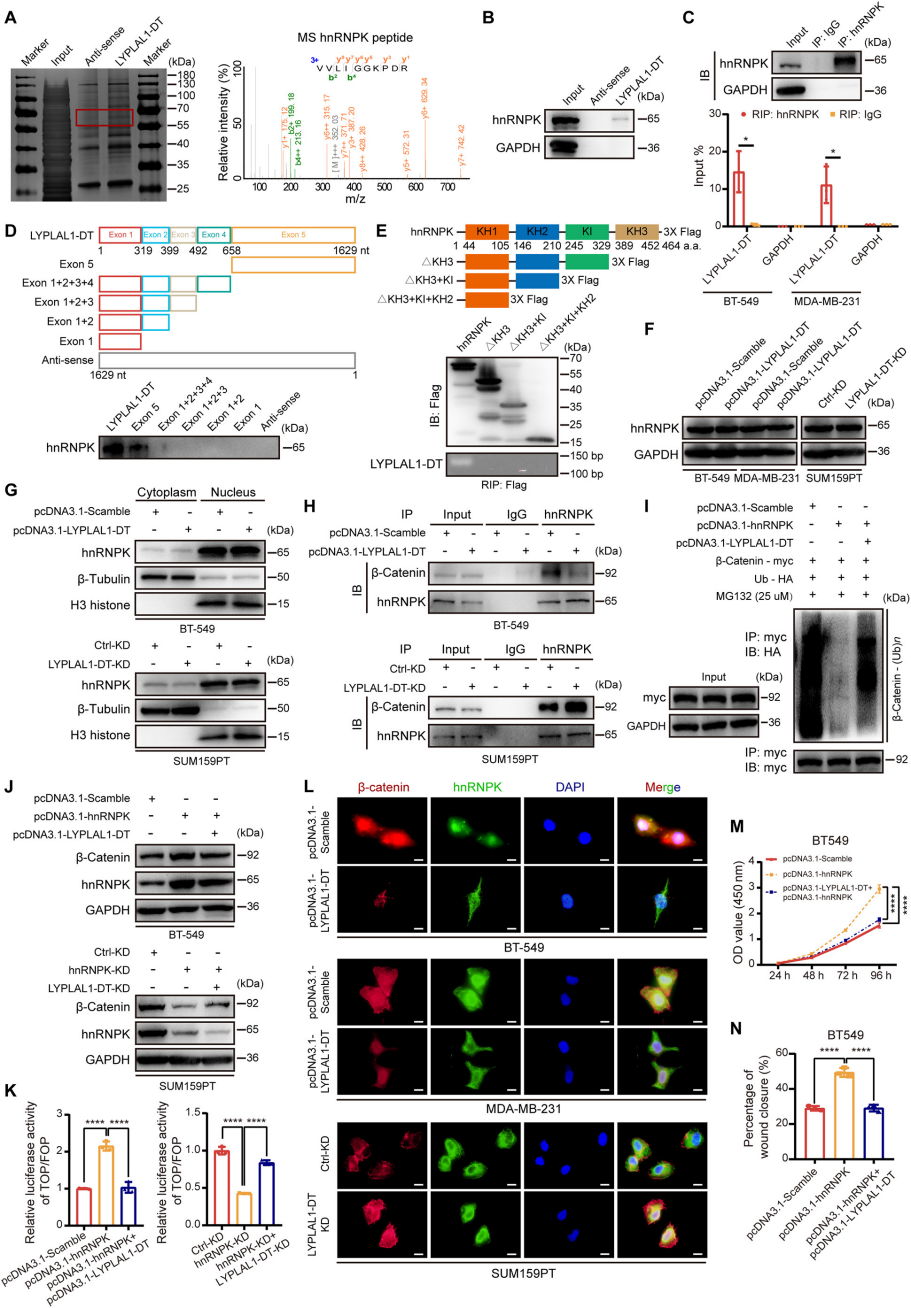
LYPLAL1-DT regulates ubiquitination of β-catenin by interacting with hnRNPK. (A) Result of RNA pull-down analysis was displayed by silver staining (left) and mass spectrometry (right). (B) Western blot for RNA pull-down analysis validating the interplay between LYPLAL1-DT and hnRNPK. (C) Validation of hnRNPK antibody used in IP assay (top) and results of RIP assay (bottom) to reconfirm the direct connection between LYPLAL1-DT and hnRNPK. (D) A schematic diagram of full-length of LYPLAL1-DT and specific exon fragments (top) and Western blot for RNA pull-down analysis using different biotinylated RNA fragments of LYPLAL1-DT (bottom). (E) A schematic picture of full domains and constructed flag-tagged truncations of hnRNPK (top) and Western blotting analysis to evaluate transfections of flag-tagged truncations of hnRNPK (medium), as well as results of RIP assays using anti-flag antibody (bottom). (F and G) Expression level of hnRNPK in whole TNBC cells (F) and in the cytoplasm or nucleus (G) after elevating or diminishing LYPLAL1-DT expression. (H) Western blotting analysis for β-catenin and hnRNPK in the IP samples with antibodies against hnRNPK or IgG in the presence or absence of overexpressed LYPLAL1-DT. (I) Ubiquitination of β-catenin was discovered in input samples (left) and IP samples using antibody against HA (right) after cotransfections under guidance of the designed groups. (J) Western blotting analyses showing that LYPLAL1-DT partly attenuated hnRNPK-induced accumulation of β-catenin in TNBC cells. (K) TOP/FOP-flash luciferase assays indicating that LYPLAL1-DT partially limited hnRNPK-generated transcriptional activity of β-catenin. (L) Representative IF images displaying alterations of colocalizations between β-catenin and hnRNPK in LYPLAL1-DT-overepressed BT-549 cells (top) or MDA-MB-231 cells (medium) and LYPLAL1-DT-silenced SUM159PT cells (bottom) (scale bar, 20 μm). (M and N) Results of CCK-8 proliferation assay (M) and quantitative data of wound-healing assays (N) after conducting cotransfections by hnRNPK plasmid and LYPLAL1-DT plasmid. Data are mean ± SD. *P < 0.05, ****P < 0.0001.

To further unravel the detailed mechanism of how LYPLAL1-DT modulated β-catenin protein, RNA pull-down assay was employed in BT-549 cells using biotinylated LYPLAL1-DT sense and antisense, and then silver staining assay and mass spectrometry were exploited to clarify the differential protein bands in sense group (Table S8), in which hnRNPK caught our attention (Fig. 6A). According to the previous researches [21,22], hnRNPK could interact directly with β-catenin and stabilize β-catenin protein, and thus, the hypothesis was drafted whether LYPLAL1-DT might affect β-catenin by gaining interaction with hnRNPK. The combination between LYPLAL1-DT and hnRNPK was validated by RNA pull-down assay and Western blotting (Fig. 6B), followed by in vitro RIP assay to reconfirm the interaction (Fig. 6C and Fig. S6H). Ulteriorly, to dig out which region(s) of LYPLAL1-DT binds to hnRNPK, specific exons of LYPLAL1-DT were in vitro transcribed to RNA and biotinylated for RIP assays, which suggested that exon 5 of LYPLAL1-DT was responsible for the interplay with hnRNPK (Fig. 6D and Fig. S6I). It is widely considered that K homology (KH) domains including KH1, KH2, and KH3 of hnRNPK were essential for the RNA-binding capability, while the K interactive (KI) domain localized between KH2 and KH3 exerted unclear effect to RNA–protein interaction [23,24]. Consequently, a series of flag-tagged truncations of hnRNPK were constructed to clarify which domain(s) of hnRNPK contributed to combine with LYPLAL1-DT and RIP assays showed that deleting the KH3 domain abolished the interplay between hnRNPK and LYPLAL1-DT (Fig. 6E and Fig. S6J), which indicated that the KH3 domain is crucial to the connection between hnRNPK and LYPLAL1-DT.

Nevertheless, LYPLAL1-DT slightly alters the overall protein level of hnRNPK in TNBC cells (Fig. 6F), together with hnRNPK protein in the cytoplasm or the nucleus of TNBC cells (Fig. 6G), manifesting that LYPLAL1-DT interacted with hnRNPK to take no effect in its expression or shuttling distribution over the cytoplasm and the nucleus. What is more, the interactions between hnRNPK and β-catenin in the presence or absence of LYPLAL1-DT were detected using co-immunoprecipitation (IP), showing that overexpressed LYPLAL1-DT sharply attenuated the interaction between hnRNPK and β-catenin, while the interplay was vitally elevated after knocking down LYPLAL1-DT (Fig. 6H). Afterward, ubiquitination assays were conducted to uncover that hnRNPK enabled to hinder ubiquitination of β-catenin, which was partly regained by promoting LYPLAL1-DT expression (Fig. 6I and Fig. S6K and L). Next, the results of Western blotting and TOP/FOP-flash luciferase reporter analyses unveiled that hnRNPK-mediated reactivation of β-catenin and the downstream signaling was partially vitiated by overexpressing LYPLAL1-DT and somewhat promoted by silencing LYPLAL1-DT (Fig. 6J and K). The influence LYPLAL1-DT exerted on the interaction between hnRNPK and β-catenin was ascertained using immunofluorescence (IF) assays in TNBC cells, implying that the simultaneous translocation of hnRNPK and β-catenin in the cytoplasm or nucleus was visibly reduced in LYPLAL1-DT overexpression status and oppositely promoted in LYPLAL1-DT knockdown status (Fig. 6L). Ultimately, the rescue functional assays comprising CCK-8 proliferation assay and wound-healing assay were exploited to figure out that overexpressing hnRNPK eliminated LYPLAL1-DT-induced tumor suppressor effects in vitro (Fig. 6M and N and Fig. S6M to P). In a nutshell, LYPLAL1-DT interacts directly with hnRNPK to disrupt the formation of the hnRNPK/β-catenin complex and thereby eliminates hnRNPK-generated stabilization of β-catenin.

### LYPLAL1-DT-induced anticarcinogenic effects were exerted by limiting abnormal reactivation of β-catenin

**Fig. 7.**
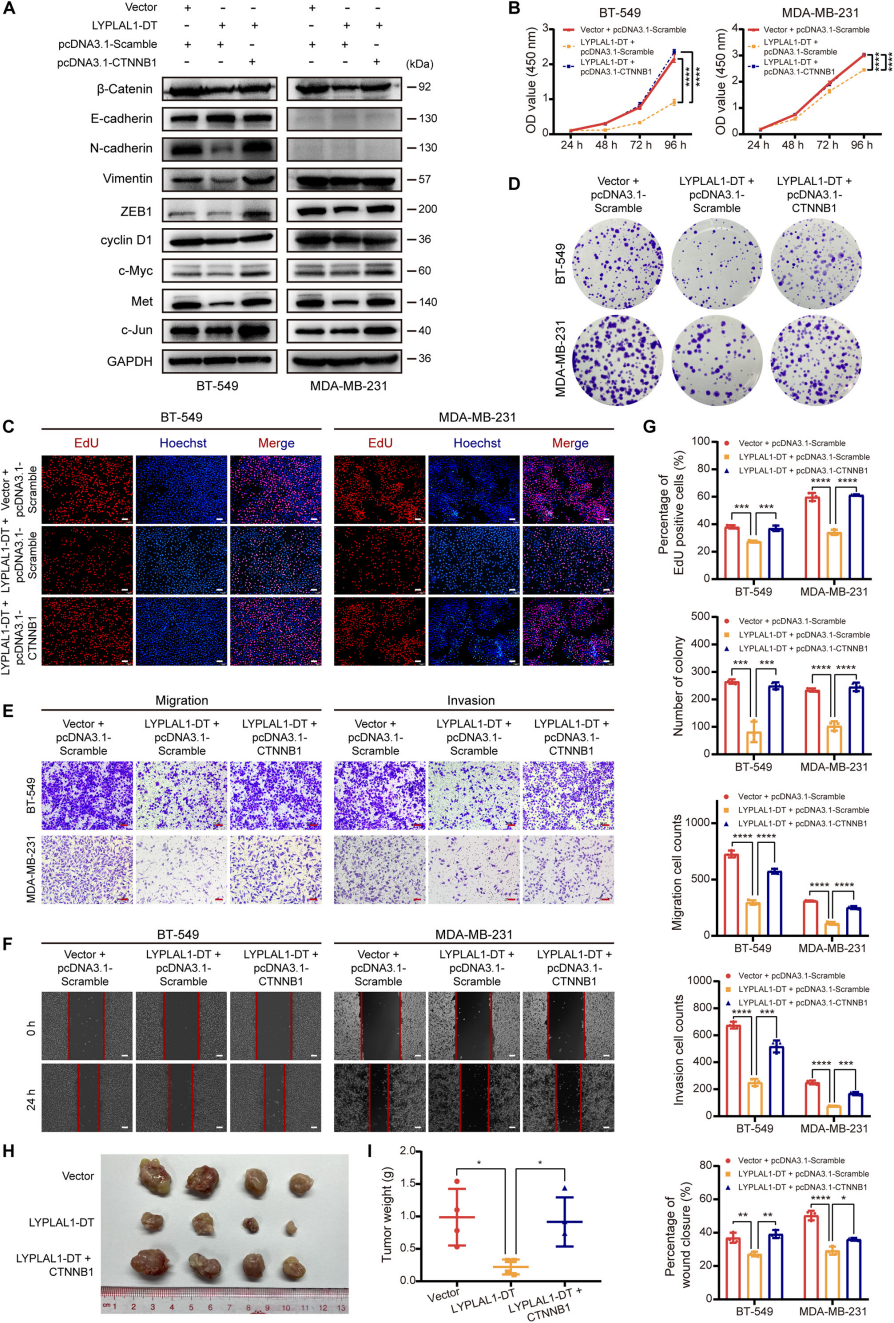
LYPLAL1-DT functions as a tumor suppressor partly by restraining aberrant reactivation of β-catenin. (A) Expression levels of β-catenin, markers of EMT, and β-catenin-activated downstream oncogenic genes were detected in stably LYPLAL1-DT-overexpressed or the control BT-549 and MDA-MB-231 cells with transfection with CTNNB1 (encoding β-catenin) plasmid or the control plasmid according to the instructions of the designed groups. (B to F) Result of CCK-8 proliferation assays (B), representative graphs of EdU staining proliferation assays (C), colony formation assays (D), transwell assays (E), and wound-healing assays (F) conducted in the designed groups above were displayed. Scale bars represent 100 μm. (G) Quantitative data of EdU staining proliferation assays, colony formation assays, transwell assays evaluating migration or invasion ability, and wound-healing assays. (H and I) Image of xenograft tumor morphology (H) and quantitative data of tumor weights of xenograft tumors (I) in nude mice (4 mice per group) injected subcutaneously into the mammary fat pad using stably LYPLAL1-DT-overexpressed MDA-MB-231 cells or stably transfected MDA-MB-231 cells simultaneously overexpressing LYPLAL1-DT and β-catenin or the control cells. Data are mean ± SD. *P < 0.05, **P < 0.01, ***P < 0.001, ****P < 0.0001.

To explore whether the anti-oncogenic function of LYPLAL1-DT was based on the limitation of β-catenin, the rescue functional experiments were performed in BT-549 and MDA-MB-231 cells stably transfected LYPLAL1-DT. Plasmid pcDNA-3.1 inserted the sequence of CTNNB1 gene encoding β-catenin (pcDNA3.1-CTNNB1), or the corresponding empty vector (pcDNA3.1-Scramble) was transfected in TNBC cells stably overexpressing LYPLAL1-DT (termed LYPLAL1-DT) or the corresponding control cells (termed Vector) under guidance of our designed groups. Initially, the results of Western blotting analysis depicted that LYPLAL1-DT-induced diminished expression of EMT markers and Wnt signaling target genes was recovered by overexpressing β-catenin (Fig. 7A). In vitro CCK-8 and EdU staining proliferation assays indicated that the inferior proliferative characteristic of TNBC cells endowed by LYPLAL1-DT was enhanced with assistance of the promoted β-catenin protein (Fig. 7B, C, and G). Subsequently, in vitro colony formation assays illuminated that the restricted colony formation ability developed by LYPLAL1-DT was reacquired in the presence of ectopically overexpressed β-catenin (Fig. 7D and G). Additionally, transwell assays and wound-healing assays delineated that β-catenin enabled to reverse the constrained migration and invasion capabilities of TNBC cells derived from LYPLAL1-DT (Fig. 7E to G). In vivo xenograft tumor models were established in nude mice using MDA-MB-231 cells simultaneously stably overexpressing LYPLAL1-DT and β-catenin (termed LYPLAL1-DT + CTNNB1), as well as Vector cells or LYPLAL1-DT cells. The results of in vivo tumor growth assays exhibited that LYPLAL1-DT-generated smaller tumors and suppressed weights of tumors regained malignant nature by virtue of β-catenin-dependent facilitated tumor growth (Fig. 7H and I). Taking the aforementioned results, LYPLAL1-DT enables TNBC cells to harbor antitumorigenesis property through negative regulation of β-catenin-activated oncogenic signaling.

### FOXO1/ LYPLAL1-DT/hnRNPK/β-catenin axis becomes a novel target in TNBC

**Fig. 8.**
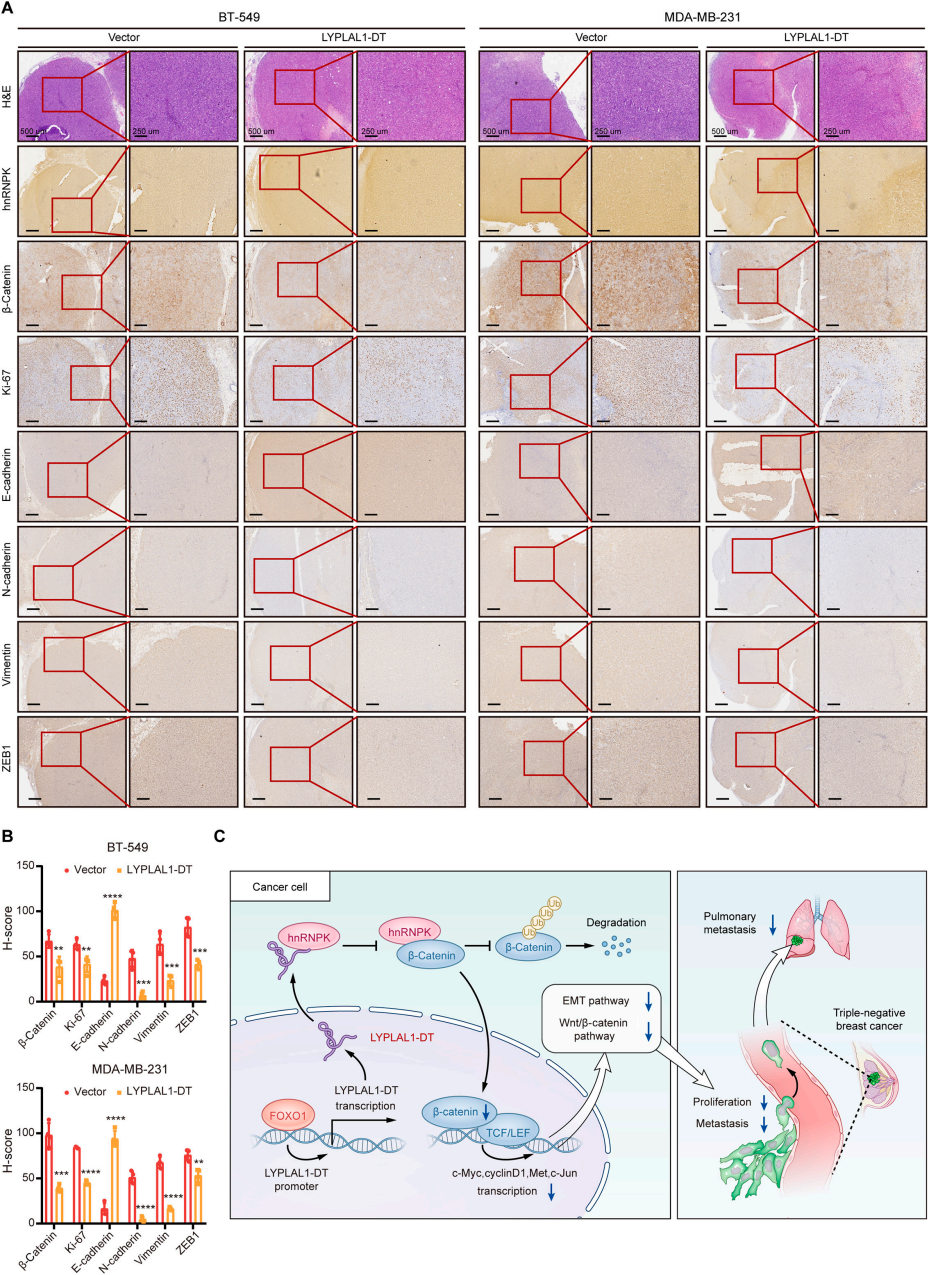
LYPLAL1-DT conserves as a novel target for TNBC treatment. (A) Representative images of H&E staining and IHC analyses using hnRNPK, β-catenin, Ki-67, E-cadherin, N-cadherin, vimentin, and ZEB1 antibodies in tissues of the xenograft tumors developed by stably LYPLAL1-DT-overexpressed BT-549 or MDA-MB-231 cells and the corresponding control cells in nude mice. (B) The expression of β-catenin, Ki-67, E-cadherin, N-cadherin, vimentin, and ZEB1 by IHC H-score in Fig. 8A. (C) Schematic view illustrating the functional role and regulatory mechanisms of LYPLAL1-DT in suppressing TNBC progression. Data are mean ± SD. **P < 0.01, ***P < 0.001, ****P < 0.0001.

To further corroborate the existence of the LYPLAL1-DT/hnRNPK/β-catenin axis in vivo, H&E staining and immunohistochemical (IHC) analyses were applied to explore the alteration of hnRNPK, β-catenin, and Ki-67 as well as EMT-related proteins in tissues of xenograft tumors from Fig. 4A, showing that expression levels of β-catenin, Ki-67, N-cadherin, vimentin, and ZEB1 were remarkably diminished in LYPLAL1-DT groups in comparison with Vector groups, and E-cadherin was substantially promoted in LYPLAL1-DT groups (Fig. 8A and B), which conformed to the results in vitro. The schematic graph demonstrating the mechanism of LYPLAL1-DT-generated suppression of tumorigenesis and metastasis in TNBC was displayed in Fig. 8C, which enlightened that the FOXO1/ LYPLAL1-DT/hnRNPK/β-catenin axis turned into an innovative target for TNBC therapy.

## Discussion

Accumulating evidence has unraveled that lncRNAs are of pivotal significance to regulate the cancerous development, treatment, relapse, and metastasis, including breast cancer [25]. Although a mass of differentially expressed lncRNAs related to TNBC development have currently been clarified, the functional roles and potential mechanism of many TNBC-implicated lncRNAs have not been elucidated precisely yet. In our present study, a novel lncRNA, LYPLAL1-DT, was disinterred to excessively diminished in TNBC samples and cell lines compared to the nontumoral control groups and correlated clinically with a superior prognosis for TNBC patients through RT-qPCR and bioinformatics analysis. A set of functional experiments unveiled that promoting LYPLAL1-DT expression eliminated the competence of proliferation and metastasis in TNBC cells, indicating the tumor suppressor impact of LYPLAL1-DT. Apart from the previous study delineating that LYPLAL1-DT exerted protective effects to human umbilical vein endothelial cells (HUVECs) in type 2 diabetes with macrovascular complication via mediation of the miR-204-5p/SIRT1 axis [26], LYPLAL1-DT has not hitherto been explored in cancers.

It has been a prevalent appreciation that TFs are crucially involved in the modulation of lncRNA expression [27]. Exemplifying TFs from forkhead box family, FOXO1 regulates LINC01197 to assist its tumor-suppressive functions in pancreatic adenocarcinoma [28]. FOXO3A induces LINC00926 to limit breast cancer growth and metastasis by dint of impeding PGK1-mediated Warburg effect [29]. FOXC1 mediates LINC00301 to promote the tumor progression and generate an immunosuppressive microenvironment in non-small cell lung cancer [30]. Likewise, our current data revealed that FOXO1 could modulate the expression of LYPLAL1-DT in TNBC by binding the LYPLAL1-DT promoter. According to the previous studies, FOXO1 mainly plays a protective factor in various cancers, especially in TNBC [31–35], which is consistent with our investigations and intensely confirmed the positively correlated expression alterations of FOXO1 and LYPLAL1-DT. Radically, our results provide more enlightenment to the regulatory effects of TFs in lncRNAs.

The functions and molecular mechanisms of lncRNAs are primarily ascertained by the subcellular localization [36,37]. For instance, lncRNAs largely localized in the nucleus, like HUMT and NRAD1, monitor the transcription of genes [9,38], while the cytoplasmic lncRNAs generally function as sponges for miRNAs to modulate the expression level of the linked mRNAs, such as ARNILA, CCAT2, and lnc015192 [39–42], along with others participating in the mediation of the cellular signaling pathway [8,43–46]. The subcellular fractionation assays and RNA-FISH experiment proved that LYPLAL1-DT was located in both cytoplasm and nucleus of TNBC cells, and the further mechanistic explorations elucidated that LYPLAL1-DT restricted the Wnt/β-catenin signaling pathway by promoting the ubiquitination of β-catenin and subsequently destabilizing its protein rather than regulating its transcription. Similarly, LINC01133 attenuated the progression of gastric cancer via sponging miR-106a-3p to alter APC expression and finally inhibit the Wnt/β-catenin signaling pathway [47]. LINC01197 reduced the proliferative property of pancreatic adenocarcinoma cells by restraining the Wnt/β-catenin signaling pathway [28]. However, RIP assay indicated that LYPLAL1-DT did not directly interact with β-catenin protein, which impelled us to further extract the direct protein partner of LYPLAL1-DT to alter the expression of β-catenin protein.

Ulteriorly, RNA pull-down assay and mass spectrometry result implied that LYPLAL1-DT could directly bind with RNA-binding protein hnRNPK, which was identified to modulate the posttranscriptional modifications of β-catenin by directly interacting with β-catenin [21,48]. Additionally, there have already been several researches uncovering the intermediating effects of hnRNPK between lncRNAs and β-catenin, and in detail, lncRNA CASC11 interacted with hnRNPK and then activated Wnt/β-catenin signaling to augment the growth and metastasis in colorectal cancer [49]. lncRNA pancEts-1 contributed to neuroblastoma development through hnRNPK-induced β-catenin stabilization [22]. SDCBP-AS1 suppressed gastric tumorigenicity and metastasis via destabilizing β-catenin by regulating the posttranscriptional modifications of hnRNPK [50]. Moreover, IP assay and Western blotting analysis illustrated that LYPLAL1-DT did not affect the overall expression and subcellular distribution in the cytoplasm and nucleus of hnRNPK but curbed the interplay between hnRNPK and β-catenin and diminished hnRNPK-mediated β-catenin stabilization. Hence, how LYPLAL1-DT mediates the interaction between hnRNPK and β-catenin is our next-step research, such as influencing the SUMOylation of hnRNPK, which is required for the direct interaction between hnRNPK and other proteins [50–52]. RNA fragment RNA pull-down assays and protein truncations RIP assays showed that exon 5 of LYPLAL1-DT and the KH3 domain of hnRNPK were responsible for the interaction between LYPLAL1-DT and hnRNPK, which gives insight into the research of interaction between RNA-binding protein and lncRNAs.

Despite that our study revealed the interaction of LYPLAL1-DT and hnRNPK, the detailed posttranscriptional modifications of hnRNPK induced by LYPLAL1-DT needed analysis in the further research. In addition, since the major complex containing APC, Axin1, and glycogen synthase kinase 3β (GSK3β) primarily triggers the degradation of β-catenin in ubiquitination-mediated proteolysis, whether LYPLAL1-DT reshapes the combination between β-catenin and degradation complex demands our investigations in the future study.

Heretofore, target therapies for the Wnt/β-catenin signaling pathway are varied and clinical experiments are nascent in a host of cancers including breast cancer [53]. We previously discovered that LGR5 could enhance the Wnt/β-catenin pathway activity to generate stemness in TNBC cells and the application of Wnt antagonist Dkk1 yielded detrimental effects to the stemness of TNBC cells [54]. Moreover, we detected that fibroblast growth factor receptor 4 (FGFR4) could trigger β-catenin/TCF4 signaling to induce anti-HER2 resistance in recalcitrant HER2-positive breast cancer and the utilization of a selective inhibitor of FGFR4, roblitinib, exhibited a potent antitumor effect on trastuzumab-resistant HER2-positive breast cancer cells [55]. As to preclinical study of antitumoral noncoding RNAs, we established a versatile hTERT-based breast cancer-specific promoter VISA composite to overexpress antitumoral circRGPD6 or miR-34a (TV-circRGPD6 and TV-miR-34a nanoparticles), which selectively increased the expression of circRGPD6 or miR-34a to suppress breast cancer stem cell-mediated growth and metastasis in vivo and in vitro [56–59]. Based on the particular biosynthesis technology and the present findings, we are currently constructing a novel TV-LYPLAL1-DT nanoparticle in this hTERT promoter-driven VISA delivery composite to selectively promote the expression of LYPLAL1-DT and impair the β-catenin pathway in TNBC, which will be focused in the sequential research.

Conclusively, our research clarified a novel tumor-suppressive lncRNA LYPLAL1-DT, which was considerably down-regulated in TNBC and clinically predicted a favorable prognosis for TNBC patients. Mechanistically, LYPLAL1-DT was transcriptionally regulated by the upstream TF FOXO1 and constrained reactivation of Wnt/β-catenin signaling in virtue of interacting with hnRNPK and destabilizing β-catenin protein, thereby diminishing cytoplasmic accumulation and nuclear translocation of β-catenin. Briefly, our study proved LYPLAL1-DT to be a potential biomarker for diagnosis and prognosis for TNBC patients.

## Materials and methods

### Clinical samples acquisition

All human TNBC tissues and the adjacent nontumoral tissues were randomly acquired from patients undergoing surgical resection from 2008 to 2011 at the Sun Yat-sen University Cancer Center (SYSUCC) in Guangzhou, China. All tissues were immediately submerged in RNAlater solution (Invitrogen, CA, USA) upon acquisition and stored at −80 °C until RNA extraction. All patients in the SYSUCC cohort provided informed consent for the utilization of specimens, and the institutional review board of SYSUCC approved this study. The public databases and in silico analysis applied in the study were illustrated in Supplementary Methods.

### Cell lines culture

The normal mammary epithelial cell line MCF-10A and all human breast cancer cell lines as well as embryo kidney cell line 293T were purchased from the American Type Culture Collection (ATCC; Manassas, VA, USA) and cultured according to standard guidelines. All cells were maintained in a humidified incubator (Thermo Fisher Scientific, MA, USA) at 37 °C with 5% CO_2_. Short tandem repeat profiling was employed to authenticate all cell lines, and no mycoplasma infection was detected in all cell lines.

### Construction and transfection of plasmids and siRNAs or ASOs

Full-length cDNAs of human LYPLAL1-DT (1,629 bp) and CTNNB1 (2,346 bp) were biosynthesized and then cloned into the plasmid vectors pcDNA3.1 and pEZ-Lv201, respectively, for transient and stable transfection (GeneCopoeia, MD, USA). Likewise, full sequences of human FOXO1 (1,968 bp) and hnRNPK (1,392 bp) cDNAs were synthesized and cloned into plasmid pcDNA3.1 (Umine Biotechnology Co., Guangzhou, China). Additionally, the promoter of LYPLAL1-DT was cloned from whole-genome DNA of MDA-MB-231 and subcloned into plasmid pEZX-FR01 (GeneCopoeia, MD, USA). Truncations of hnRNPK were obtained with appropriate primers from PCR and subcloned into pcDNA3.1 with 3X Flag c-tag. The corresponding empty vectors were used as an overexpression control (Scramble, pcDNA3.1-Scramble, or Vector). All abovementioned constructions were validated by sequencing. A mixture of three siRNAs and three antisense oligonucleotides (ASOs) for knocking down LYPLAL1-DT was provided by Ribobio Corporation (Guangzhou, China), and other siRNAs targeting FOXO1 and hnRNPK were biosynthesized by GenePharma Corporation (Shanghai, China), while nontargeting siRNA was employed as a control of knockdown (siCtrl). All the detailed sequences of siRNAs or ASOs above are provided in Table S1. All transfections were finalized with the Lipofectamine 3000 kit (Invitrogen, CA, USA) following the manufacturer’s instructions.

### RNA attainment and real-time quantitative PCR analysis

Total RNA of tissue samples and breast cancer cell lines was attained with TRIzol reagent (Invitrogen, CA, USA) under guidelines of the manufacturer and subsequently transcribed reversely into cDNA in virtue of reverse transcription system mixture (Takara Bio Inc., Shiga, Japan) as previously described [60]. Afterward, RNA levels were investigated through RT-qPCR with three independent replicates utilizing the SYBR Green method (Takara Bio Inc., Shiga, Japan) on a Bio-Rad CFX96. lncRNA or mRNA expression was normalized against β-actin for each sample, and the relative RNA expression was determined by means of the 2^−ΔΔCt^ method. Table S2 displayed all the sets of primers.

### Western blotting assay

Proteins isolated from cell lines were segregated by SDS-PAGE with electrophoresis and transferred onto polyvinylidene difluoride (PVDF) membrane (Millipore, MA, USA). Afterward, the membrane was subsequently blocked in tris-buffered saline with Tween (TBST) containing 5% skim milk and incubated with primary antibody overnight at 4 °C. Ulteriorly, the membrane was exposed to secondary antibody for 1 h at room temperature. Eventually, the protein bands were investigated by chemiluminescence using ECL Western Blotting Substrate (Affinity Biosciences, OH, USA). All the antibodies for Western blot analysis are exhibited in Table S3.

### IP, ChIP, RIP, and RNA pull-down assay and mass spectrometry

IP assays were executed using IP lysis buffer (Beyotime, Shanghai, China) adding protease and phosphatase inhibitors (Beyotime, Shanghai, China), the appropriate antibodies, and protein A/G magnetic beads (MCE, Shanghai, China). ChIP assays were accomplished with the Magnetic Bead ChIP Kit (Thermo Fisher Scientific, MA, USA) according to the manufacturer’s protocols. RIP assays were employed with the Magna RIP RNA-Binding Protein Immunoprecipitation Kit (Millipore, MA, USA) conforming to the instructions of the manufacturer as previously described [9,61]. RNA pull-down assays were finalized exploiting Pierce Magnetic RNA-Protein Pull-Down Kit (Thermo Fisher Scientific, MA, USA) following the guidelines as previously described [9]. Moreover, the specific strips originated from RNA pull-down assay were cut for mass spectrometry, which was performed at Fitgene Corporation (Guangzhou, China). The detailed procedures of IP, ChIP, RIP, and RNA pull-down assays were demonstrated in Supplementary Methods.

### Cell counting kit-8 (CCK-8), EdU staining, colony formation, in vitro transwell assays and wound-healing assay

In vitro functional experiments including CCK-8, EdU staining assay, colony formation assay, transwell assays, and wound-healing assay were completed in accordance with the exhaustive processes depicted in Supplementary Methods.

### In vivo xenograft tumor growth and lung metastasis assays

Three- to 4-week-old female BALB/c nude mice (Vital River, Beijing, China) were injected subcutaneously in the fat pads of breast using 5 × 10^6^ MDA-MB-231 cells or 1 × 10^7^ BT-549 cells both stably ectopically overexpressing LYPLAL1-DT and the corresponding control cells after a week quarantine. Approximate tumor volume (mm^3^) was gauged weekly and determined following the formula (tumor volume = length × width^2^ /2) as previously described [9]. The mice were euthanized in 28 days, and the tumors were harvested and instantly weighted, which eventually were fixed with 4% paraformaldehyde overnight and used for further IHC and H&E staining analyses, which were portrayed in Supplementary Methods.

Model of lung metastasis was generated in female nude mouse through intravenous injection via the tail vein using 2 × 10^6^ luciferase-labeled MDA-MB-231 or BT-549 stably overexpressing-LYPLAL1-DT and the corresponding control cells. At 60 days after injection, the mice were examined by in vivo optical luciferase imaging assay and eventually sacrificed, and the lungs were obtained, immediately imaged by in vivo optical luciferase imaging assay, and photographed after fixing with 4% paraformaldehyde overnight. Ultimately, the lung tissues were embedded in paraffin and prepared for further H&E staining analysis. The number of metastatic foci was calculated from lung H&E staining slides. In vivo optical luciferase imaging assays were performed with Xenogen IVIS Spectrum (Xenogen, CA, USA) after mice were injected with 100 μl of 15 μg/μl d-luciferin (Yeason, Shanghai, China) intraperitoneally, and fluorescence images were taken setting the exposure time to 1 s. All the animals’ procedures were conducted by approval of the Sun Yat-Sen University Animal Care and Use Committee.

### Promoter dual-luciferase reporter assay and TOP/FOP-flash luciferase reporter analysis

Promoter dual-luciferase reporter assay and TOP/FOP-flash luciferase reporter analyses were performed utilizing the Dual-Luciferase Reporter Assay Kit (Promega, WI, USA) according to the protocol of the manufacturer, which was concisely illuminated in Supplementary Methods.

### RNA-FISH and subcellular fractionation

To unravel the subcellular distribution of LYPLAL1-DT in TNBC cells, RNA-FISH was performed using Cy3-labled RNA probes for LYPLAL1-DT, 18S, and U6 (Table S1) and Fluorescent In Situ Hybridization Kit (GenePharma, Shanghai, China) and 4′,6-diamidino-2-phenylindole (DAPI) solution (Solarbio, Beijing, China) following the manufacturer’s instructions as previously described [62]. Images were captured with a fluorescence microscope (Olympus, Tokyo, Japan).

The subcellular fractionation was finalized with PARIS kit (Invitrogen, CA, USA) for further RT-qPCR and Western blot analyses under guidance of the manufacturer. RNA expression of LYPLAL1-DT in fractions of the cytoplasm and the nucleus was verified by RT-qPCR analysis.

### CHX-chase assay and Ubiquitination assay

CHX-chase assay was undertaken with CHX (Sigma-Aldrich, Darmstadt, Germany), serving as the protein synthesis inhibitor. In detail, 50 μg/ml of CHX mixed in serum-free medium was employed to treat the cells in six-well plates, and β-catenin protein was investigated through Western blot analysis at 0, 2, 4, and 6 h. Furthermore, 25 μM MG132 (Sigma-Aldrich, Darmstadt, Germany), the proteasome inhibitor, was used to rescue CHX-chase assay.

For ubiquitination assay, 293T cells were cotransfected with hemagglutinin (HA)-tagged ubiquitin vector (Ub-HA), myc-tagged β-catenin vector (β-catenin-myc), and LYPLAL1-DT overexpression vector or LYPLAL1-DT knockdown silencer, along with the corresponding controls. At 24 h after transfection, cells were incubated with 25 μM MG132 for 6 h. Cells were treated with IP lysis buffer, and IP assay was executed with myc tag antibody following the aforementioned procedures. Western blot analysis was applied to measure all the proteins.

### IF analysis

The cells were seeded onto chamber slides and fixed with 4% formaldehyde solution, followed by penetration with 1% Triton X-100. In addition, cells were incubated with primary antibodies against β-catenin and hnRNPK (Table S3) simultaneously overnight at 4 °C and subsequently incubated with CoraLite594-conjugated goat anti-rabbit and CoraLite488-conjugated goat anti-mouse secondary antibodies (Table S3), while the cell nuclei were stained with DAPI solution. Last, the cells were imaged with a fluorescence microscope (Olympus, Tokyo, Japan).

### Statistical analysis

All statistical analyses were executed via RStudio software (version 4.0.2) and GraphPad Prism 8 software. Univariate and multivariate Cox regression analyses based on OS of patients in the TCGA-TNBC cohort were conducted to clarify the clinical value of promising lncRNAs and especially whether LYPLAL1-DT was an independent prognostic predictor of TNBC. The log-rank test was employed in each Kaplan–Meier curve to compare the survival rates between groups. Paired sample Student’s *t* test was exploited to contrast the expression of LYPLAL1-DT between paired tumor tissues and nontumoral tissues. Pearson correlation test was applied to screen out TFs significantly correlated with LYPLAL1-DT. Spearman correlation test was accomplished to seek significant LYPLAL1-DT-correlated genes for further functional enrichment analyses. Comparisons were implemented by dint of the two-tailed Student’s *t* test between two groups and one-way analysis of variance (ANOVA) test with Bonferroni correction among three groups, as well as two-way ANOVA test with Holm–Sidak correction in CCK-8 and in vivo xenograft tumor growth experiments. All experiments were executed at least in triplicate. For statistical scatter plots and bar plots, data were shown as the mean ± SD. A two-tailed value of *P* < 0.05 was determined statistically significant.

## Data Availability

The data for bioinformatics analysis in this study are derived primarily from TCGA and GEO databases. Other data and materials from other public databases or biotechnical companies are provided in our article and additional files.
