# Supplementary material for: Dissection of FOXO1-Induced LYPLAL1-DT Impeding Triple-Negative Breast Cancer Progression via Mediating hnRNPK/β-Catenin Complex
Source: Research (Wash D C). 2023 Dec 15;6:0289. doi: 10.34133/research.0289 (PMC10726293; doi:10.34133/research.0289)
Supplement: Supplementary 1 — Supplementary Methods Figs. S1 to S6 Tables S1 to S8 [file research.0289.f1.docx]

**Supplementary Figure Legends**

**
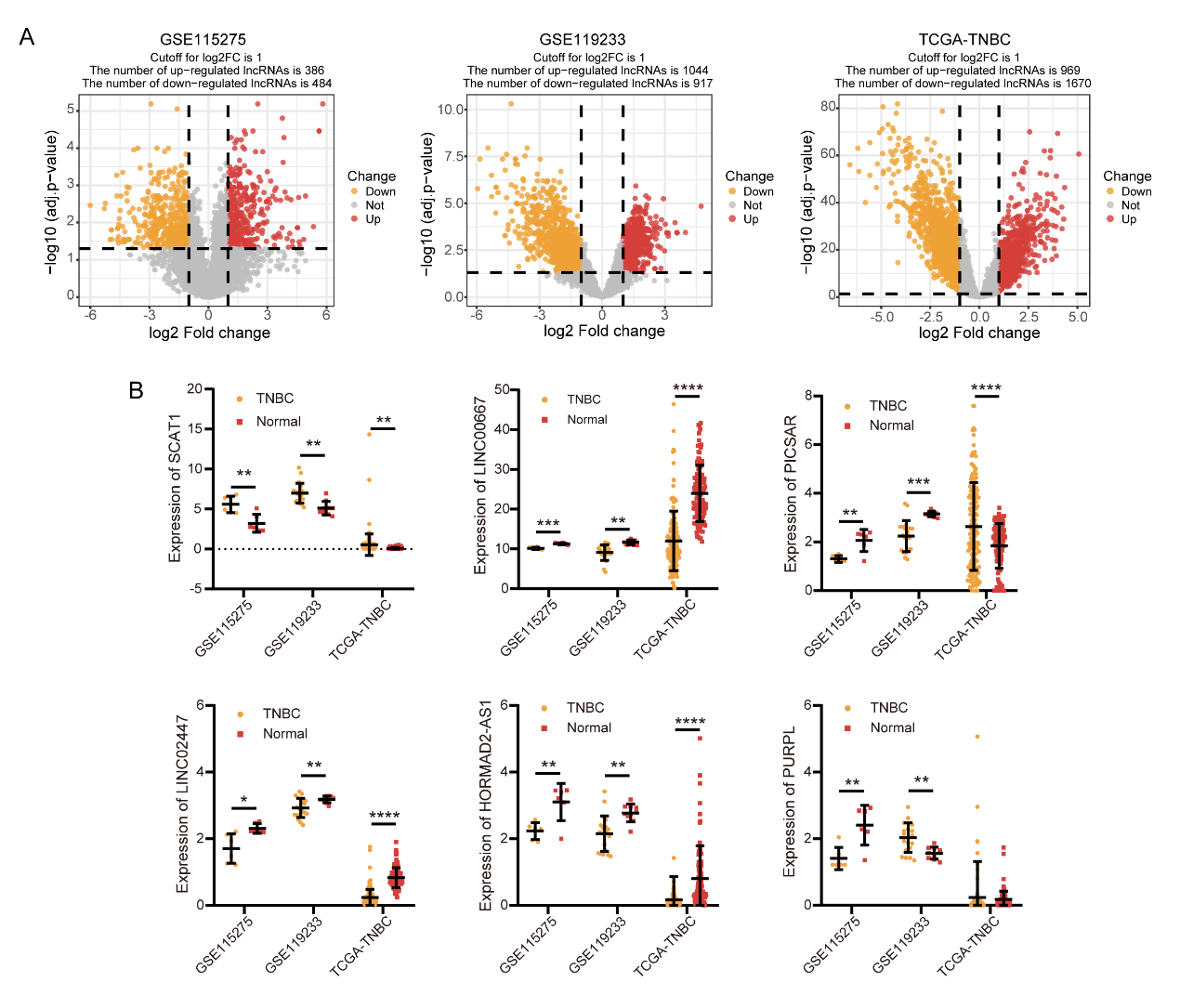
**

**Supplementary Figure S1.** Significant differentially expressed lncRNAs are screened out.

**A** Volcano maps illustrating the identifications of significant differentially expressed lncRNAs in GSE115275, GSE119233 and TCGA-TNBC cohorts, respectively. **B** Expression level of SCAT1, LINC00667, PICSAR, LINC02447, HORMAD2-AS1 and PURPL in TNBC and non-tumor Normal samples of three datasets, respectively. Error bars represent mean ± SD. * means *p* < 0.05, ** means *p* < 0.01, **** means *p* < 0.0001.


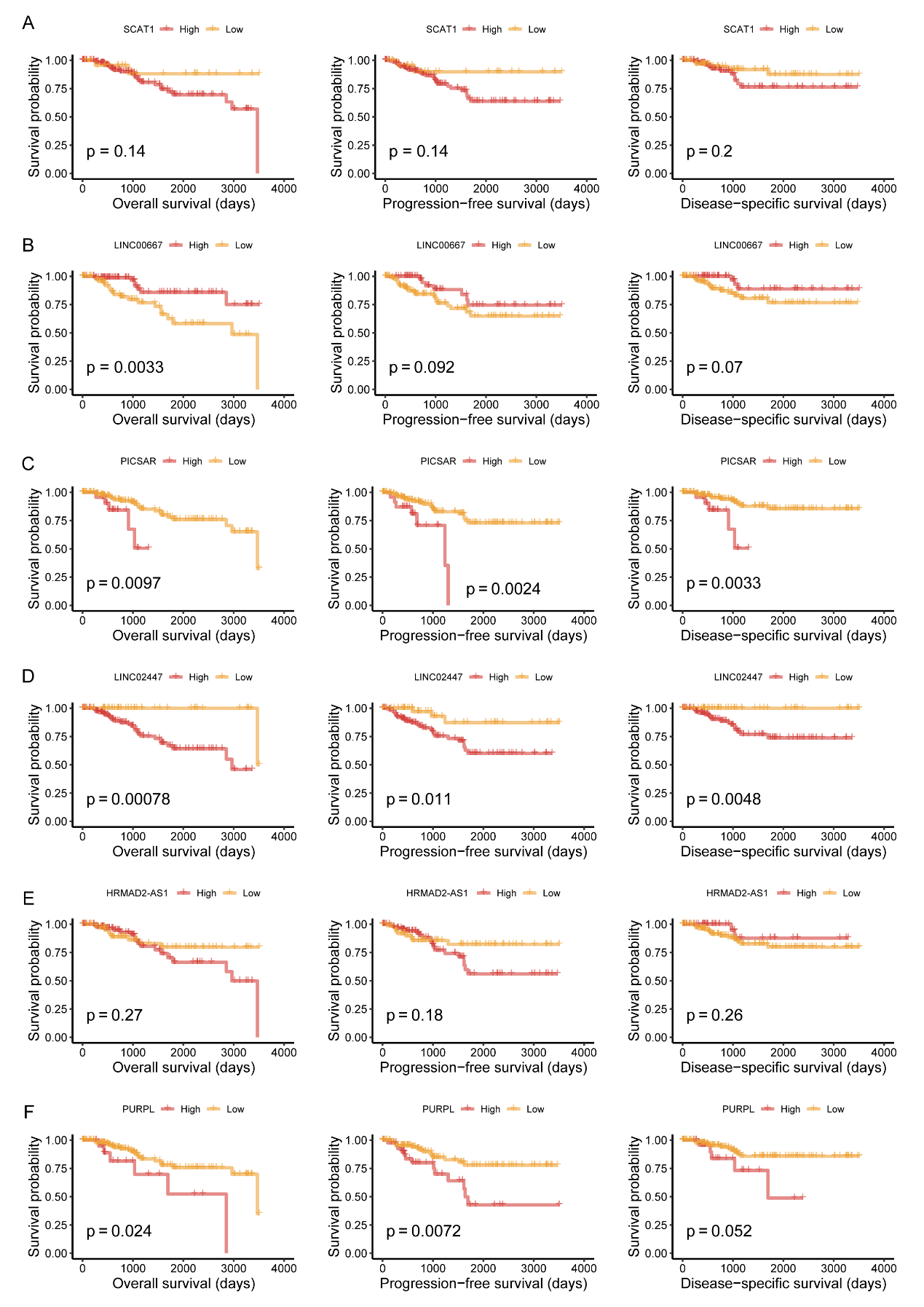


**Supplementary Figure S2.** Kaplan-Meier curves for overall survival, progression-free survival and disease-specific survival based on the expression of SCAT1 (**A**), LINC00667 (**B**), PICSAR (**C**), LINC02447 (**D**), HORMAD2-AS1 (**E**) and PURPL (**F**) in TCGA-TNBC cohort.


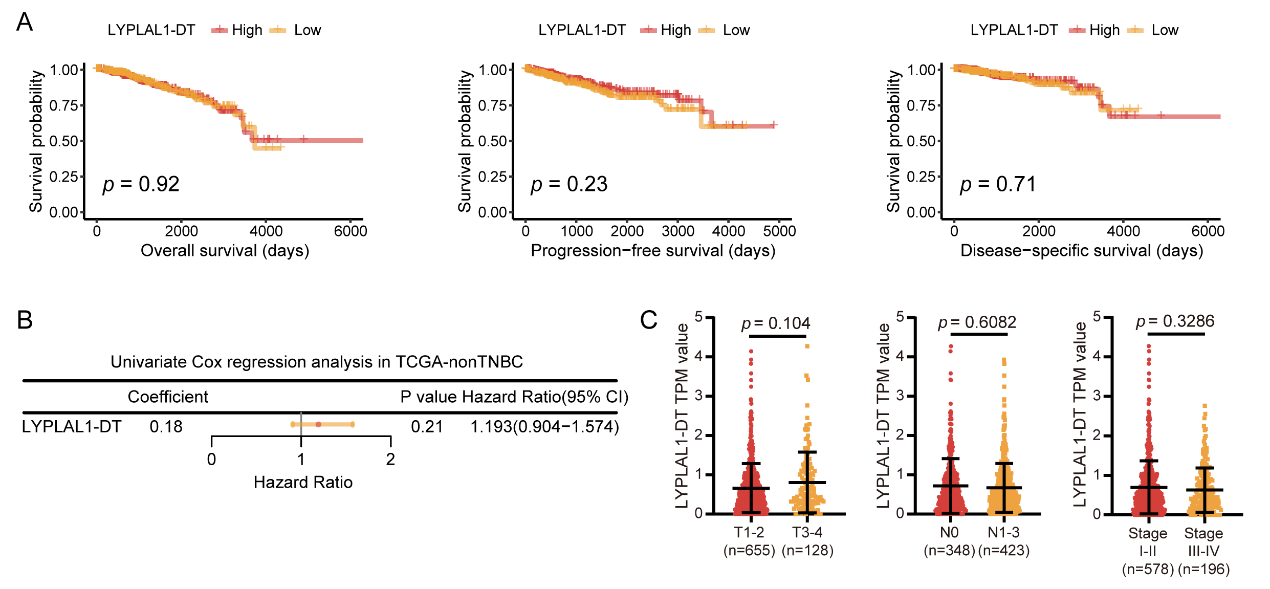


**Supplementary Figure S3.** LYPLAL1-DT expression is not relevant to the prognosis of non-TNBC patients.

A Kaplan-Meier curves for overall survival, progression-free survival and disease-specific survival based on LYPLAL1-DT expression in TCGA-nonTNBC cohort (n = 789). **B** Univariate Cox regression analysis based on LYPLAL1-DT in TCGA-nonTNBC cohort. **C** Expression levels of LYPLAL1-DT with various T stage, N stage or AJCC Stage from TCGA-nonTNBC cohort.


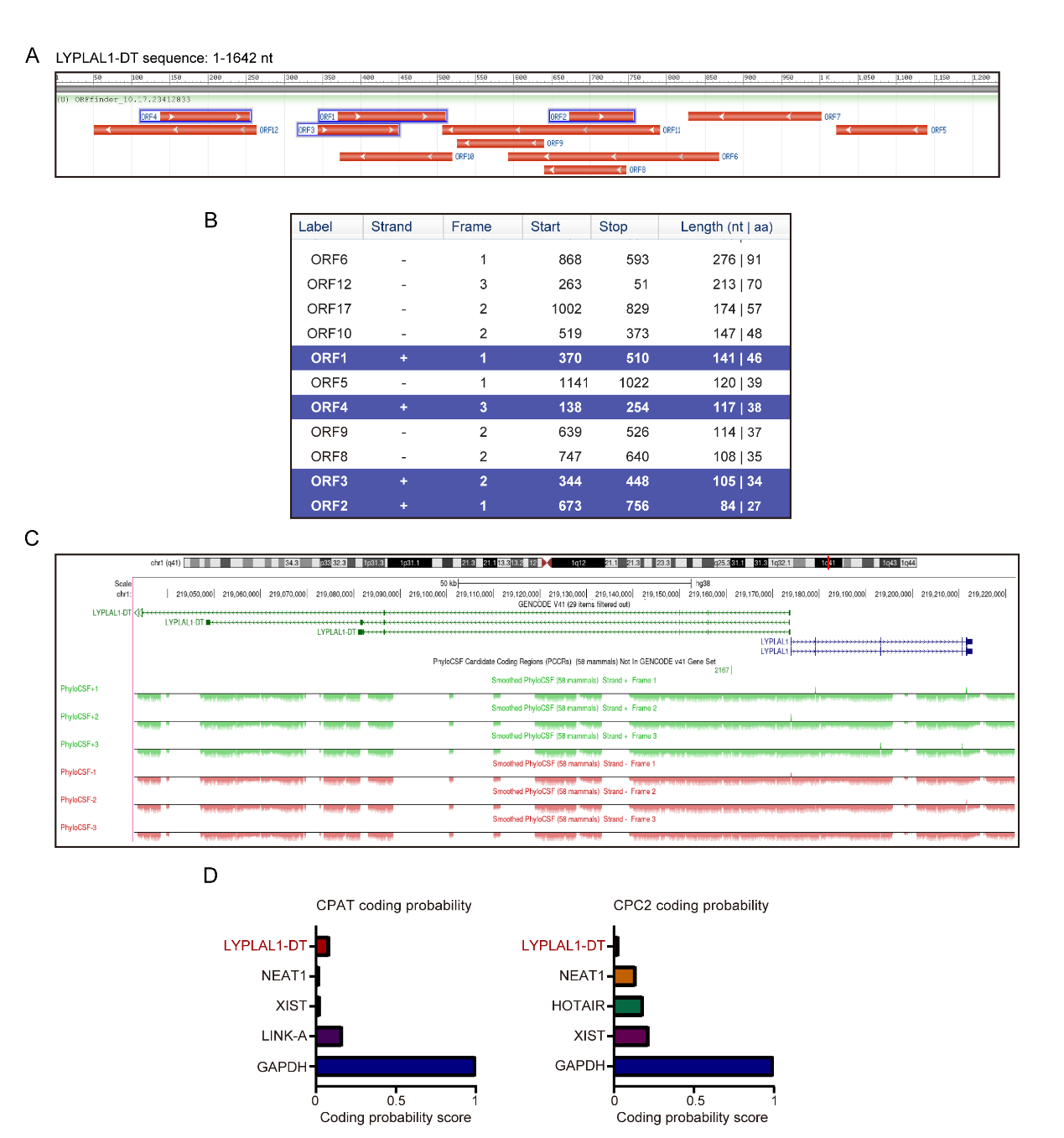


**Supplementary Figure S4.** LYPLAL1-DT has no protein-coding potential.

**A-B** Location (**A**) and length (**B**) of open read frame in the plus-strand of LYPLAL1-DT. **C** PhyloCSF model was employed to predict the coding potential of LYPLAL1-DT. **D** Coding probability score of LYPLAL1-DT, several typical lncRNAs and GAPDH calculated in CPAT (left) and CPC2 (right) web tools.


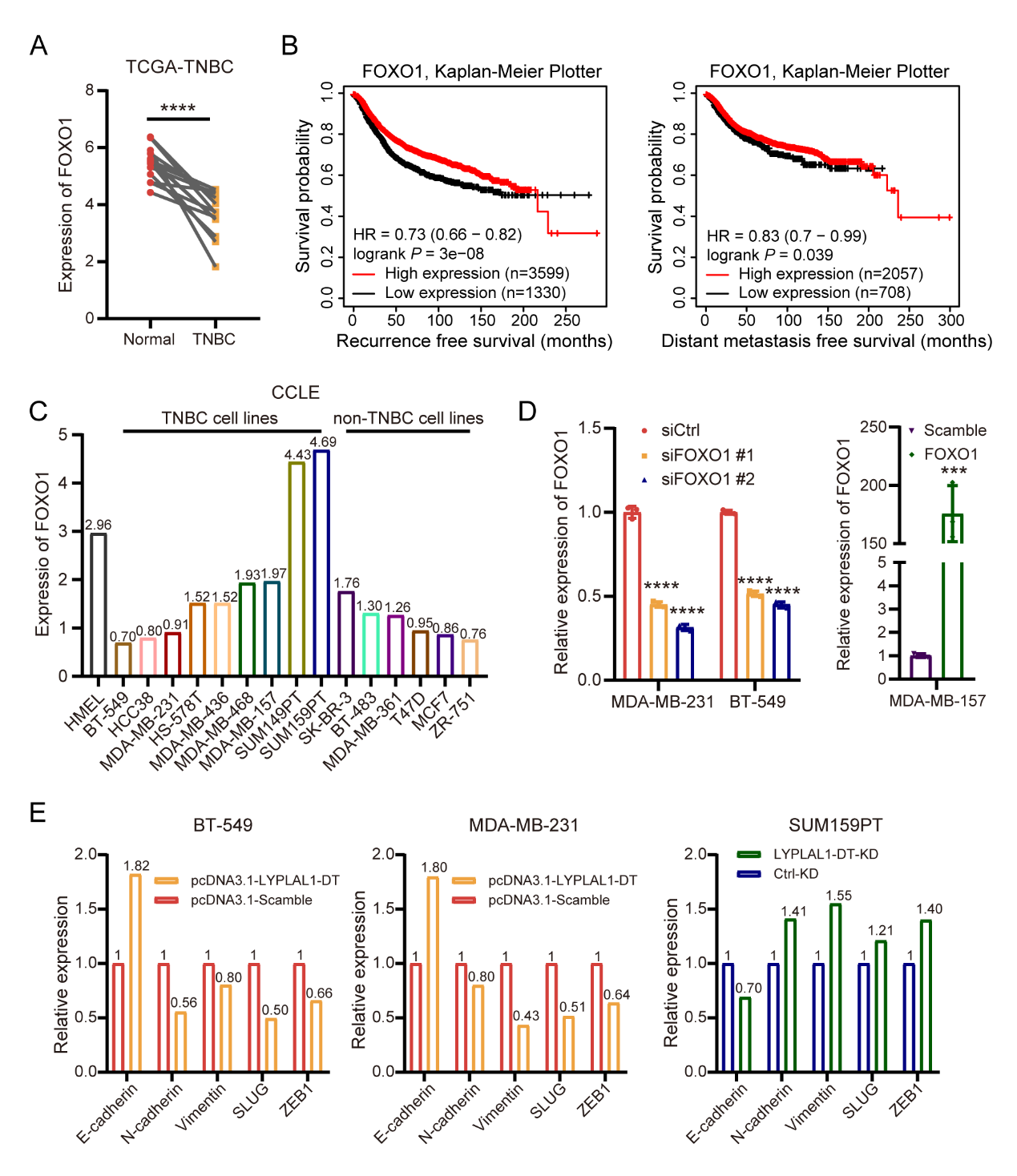


**Supplementary Figure S5.** FOXO1 is down-regulated in TNBC cohort and related to a favorable prognosis.

**A** Expression level of FOXO1 in 14 pairs of TNBC samples and matched non-tumoral Normal samples from TCGA-TNBC cohort. **B** Kaplan-Meier curves to predict recurrence free survival (left) and distant metastasis free survival (right) for patients with breast cancer based on expression of FOXO1 in Kaplan-Meier Plotter website. **C** Expression level of FOXO1 in a series of BC cell lines and non-cancerous mammary epithelial cell line HMEL according to CCLE database. **D** Validation of RNA expression after silencing or overexpressing FOXO1 in TNBC cells. Data were shown by mean ± SD. ** means *p* < 0.01, *** means *p* < 0.001, **** means *p* < 0.0001.


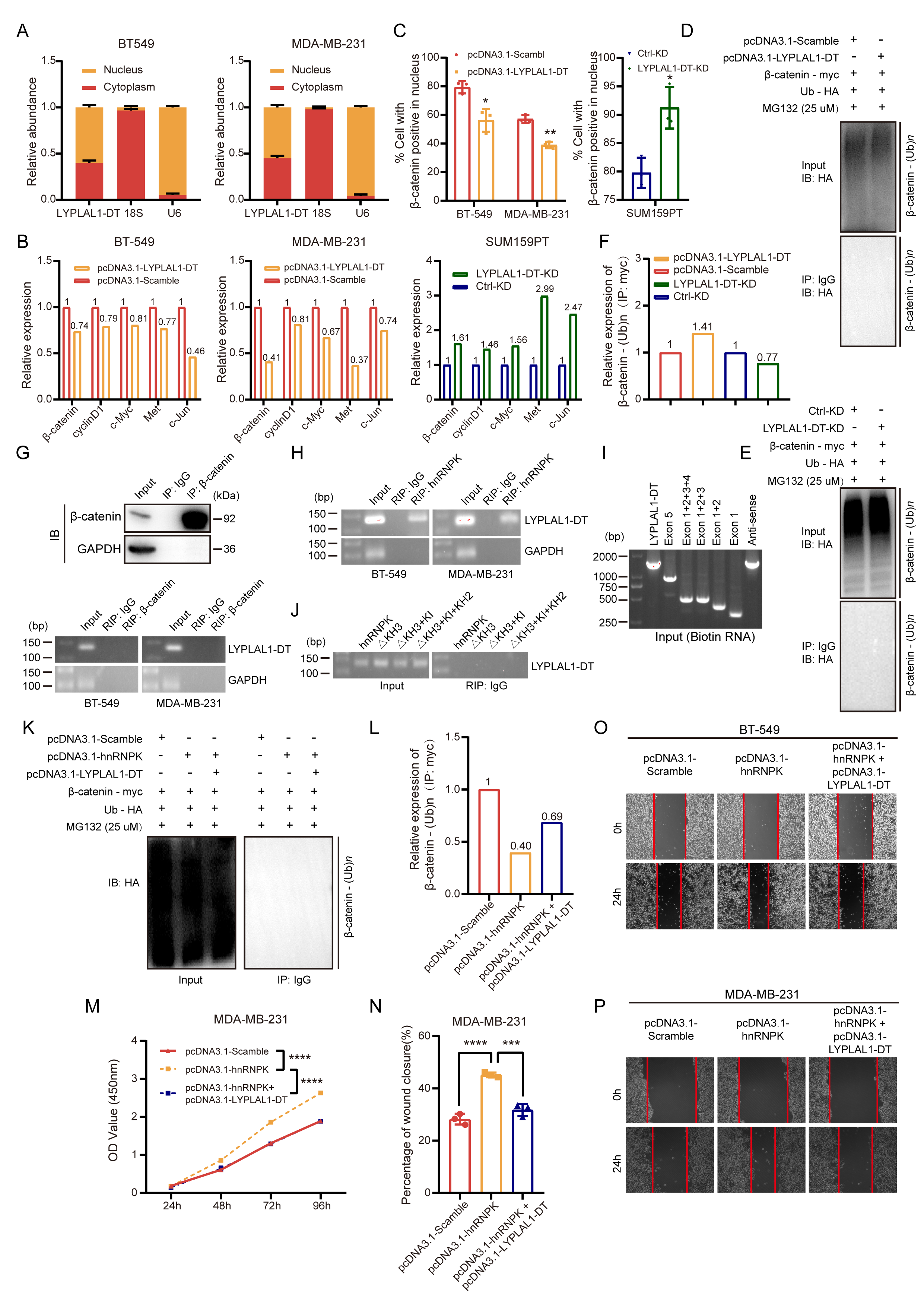


**Supplementary Figure S6.** The underlying mechanism of LYPLAL1-DT in TNBC was elucidated in supplementary proof.

**A-C** Statical analyses of RNA-FISH assay in **Fig 5B** (**A**), expression levels of protein in Western blots dataset of **Fig. 5F** (**B**) and ICC assays in **Fig 5J** (**C**). **D-E** Ubiquitination of β-catenin was investigated in input or IgG control samples using antibody against HA after co-transfecting myc-tagged β-catenin plasmid, HA-tagged ubiquitin plasmid and LYPLAL1-DT plasmid (**D**) or LYPLAL1-DT knockdown mixtures (**E**) in 293T cells. **F** Statical analyses of expression level of the ubiquitinated β-catenin in **Fig. 5M-N.** **G** Validation of β-catenin antibody employed in IP assay (top) and results of RIP assay (bottom) to clarify LYPLAL1-DT did not directly interact with β-catenin. **H** Confirmation of products from RIP by hnRNPK and IgG antibodies and subsequent RT-qPCR analysis using nuclear acid electrophoresis. **I** Results from nuclear acid electrophoresis to verify the different biotinylated RNA fragments of LYPLAL1-DT. **J** Products of amplifying LYPLAL1-DT by RIP using flag antibody and RT-qPCR analysis in input samples and IgG samples were validated via nuclear acid electrophoresis. **K** Ubiquitination of β-catenin was investigated in input or IgG control samples of the designed groups. **L** Statical analyses of expression level of the ubiquitinated β-catenin in **Fig. 6I**. **M-N** Results of CCK-8 proliferation assay (**M**) and quantitative data of wound-healing assays (**N**) after conducting co-transfections by hnRNPK plasmid and LYPLAL1-DT plasmid in MDA-MB-231 cells. **O-P** Representative graphs of wound-healing assays after conducting co-transfections by hnRNPK plasmid and LYPLAL1-DT plasmid in BT-549 (**O**) and MDA-MB-231 cells (**P**). Data were shown by mean ± SD. * means *p* < 0.05, ** means *p* < 0.01. *** means *p* < 0.001. **** means *p* < 0.0001.

**Supplementary Methods**

**Public databases and bioinformatics analysis**

The transciptome expression data for lncRNAs in TNBC samples and normal breast samples were derived from The Cancer Genome Atlas (TCGA) database (<https://portal.gdc.cancer.gov/repository>) and GSE115275 and GSE119233 in the Gene Expression Omnibus (GEO) database (<https://www.ncbi.nlm.nih.gov/geo/>). The selective criteria for TCGA-TNBC cohort were as follows: 1) samples were available for Overall survival (OS) data and mRNA and lncRNA expression data; 2) the molecular subtypes were confirmed by immunohistochemistry (IHC) and Her-2 status was further determined by fluorescence in situ hybridization (FISH) if intermediate positive in IHC. Therefore, this study enrolled 160 TNBC samples and 113 normal breast samples from TCGA-TNBC cohort, 6 TNBC samples and matched non-tumoral samples from GSE115275 cohort^1^, as well as 20 TNBC samples and 10 normal breast samples from GSE119233 cohort^2^ for initial potential lncRNAs selection and further bioinformatics analysis. The University of Alabama at Birmingham Cancer data (UALCAN) analysis Portal^3^ (<http://ualcan.path.uab.edu/>) was utilized to investigate the expression level of LYPLAL1-DT across cancers and adjacent non-tumoral tissues from TCGA database. To screen out the meaningful differentially expressed lncRNAs between TNBC samples and non-tumoral breast samples, “limma” R package^4^ was applied in TCGA-TNBC cohort, GSE115275 cohort and GSE119233 cohort as the significant criteria were set to |log2FC| > 1 and adjust *p* value < 0.05. The coding probability of LYPLAL1-DT was assessed via the public website Open Reading Frame Finder (<https://www.ncbi.nlm.nih.gov/orffinder/>), PhyloCSF software, Coding-Potential Assessment Tool (<http://lilab.research.bcm.edu/cpat/>) and Coding Potential Calculator 2 (<http://cpc2.gao-lab.org/>) ^5,6^. The transcription factors (TFs) that probably regulated the expression of LYPLAL1-DT were predicted using JASPAR database (<https://jaspar.genereg.net/>). Expression levels of FOXO1 in a series of BC cell lines were obtained from CCLE database (https://sites.broadinstitute.org/ccle/). The ChIP-seq datasets related to FOXO1 were retrived from ENCODE database (<https://www.encodeproject.org>) and the mapping result in the promoter of LYPLAL1-DT was visualized in UCSC database (<http://genome.ucsc.edu/>). Kaplan-Meier survival plots of OS, recurrence free survival (RFS) and distant metastasis free survival (DMFS) of breast cancer patients on the basis of FOXO1 expression were conducted in the Kaplan-Meier Plotter website (<http://kmplot.com/analysis/index.php?p=service&cancer=breast>)^7^. Functional enrichment analyses utilizing Kyoto Encyclopedia of Genes and Genomes (KEGG) and Gene Ontology (GO) pathways were executed via DAVID database (<https://david.ncifcrf.gov/>) based on significant LYPLAL1-DT-correlated genes and GSEA software based on the matrix separated to LYPLAL1-DT high-expression and low-expression subgroups in TCGA-TNBC cohort under guidance of instructions^8-10^.

**Construction of lentivirus in TNBC cells**

Stable LYPLAL1-DT-overexpressing and CTNNB1-overexpressing TNBC cell lines were generated via lentivirus produced in 293T cell line after transiently transfecting pEZ-Lv201-LYPLAL1-DT, pEZ-Lv201-CTNNB1 or the empty vector with two packaging assistant lentivirus vectors to 293T according to the guidance of manufacturer and screened by treatment of puromycin (Yeasen Biotechnology Co., Shanghai, China). All transfections were finalized with assist of the Lipofectamine 3000 kit (Invitrogen, CA, USA) following the manufacturer’s instructions.

**Immunoprecipitation (IP)**

Cells were collected from 10 cm dishes and lysed with 1 ml of IP lysis buffer (Beyotime, Shanghai, China) adding protease and phosphatase inhibitors (Beyotime, Shanghai, China) on ice for 30 min. After completing centrifugation (12,000 g, 15 min, 4 °C), the supernatant was divided into 100 μL (for input), 450 μL (for IgG) and 450 μL (for IP) aliquots. Then, an isotype-matched antibody against IgG or the appropriate antibodies specific for FOXO1, myc tag, β-catenin, hnRNPK and Flag tag were added in the 450 ul supernatant that gained incubation overnight at 4 °C. Thereafter, 50 microliters of protein A/G magnetic beads were added into the farrago of antibodies and supernatant and rotated for 2 hours at 4 °C. Afterwards, the precipitates were washed 5 times with IP lysis buffer to remove all unbound proteins and the immune complexes were added with 40-60 ul of 1X loading dye and then boiled. Ultimately, the precipitates with loading dye were utilized to Western blotting analysis with the suitable antibodies.

**Chromatin immunoprecipitation (ChIP)**

1% formaldehyde was applied for DNA crosslink with cells in 10 cm dishes for 10 min at room temperature while glycine was used to terminate the crosslinking reaction. And Magnetic Bead ChIP Kit (Thermofisher, MA, USA) were subsequently employed following the manufacturer’s protocols. In brief, the DNA-protein complex was digested by MNase and sonicated to produce 200-800bp DNA fragments. After incubation with anti-FOXO1 antibody (18592-1-AP, Proteintech, Wuhan, China) or negative control IgG antibody (30000-0-AP, Proteintech, Wuhan, China) overnight at 4 °C, ChIP grade protein A/G magnetic beads were exploited for immunoprecipitation of DNA-protein complex in lysates. Eventually, the retrieved DNA was amplified using the designed primer sets for RT-qPCR and nuclear acid electrophoresis. And the primer sets applied in ChIP are presented in **Supplementary Table 2**.

**RNA immunoprecipitation (RIP)**

RIP assays were accomplished with the Magna RIP RNA-Binding Protein Immunoprecipitation Kit (Millipore, MA, USA) conforming to the instructions of the manufacturer as previously described ^11,12^. Concisely, 1 × 10^7^ TNBC cells were gained and lysed using RIP lysis buffer, and the lysate was added with the magnetic beads conjugated anti-hnRNPK antibody (11426-1-AP, Proteintech, Wuhan, China) or anti-β-catenin antibody (8480, CST, MA, USA) or negative control IgG antibody (30000-0-AP, Proteintech, Wuhan, China). Ulteriorly, the beads were thoroughly washed and proteinase K was utilized to digest the proteins and purify the RNAs. And the extracted RNA was detected by RT-qPCR analysis using LYPLAL1-DT and GAPDH primers (**Supplementary Table 2**) and validated with nuclear acid electrophoresis.

**RNA pull-down assay and mass spectrometry**

RNA pull-down assays were finalized exploiting Pierce Magnetic RNA-Protein Pull-Down Kit (Thermofisher, MA, USA) following the guidelines as previously described^11^. In brief, the sense or antisense LYPLAL1-DT and specific exons of LYPLAL1-DT were transcribed in vitro and biotinylated by MAXIscript T7 Transcription Kit (Invitrogen, CA, USA) conforming to the manufacturer’s protocols. The biotinylated RNA probe was mixed with streptavidin magnetic beads to gain magnetic beads-probe complex for one hour at room temperature. Afterwards, the magnetic beads-probe complex was incubated with total BT-549 cell lysates overnight at 4°C and the bead-RNA-protein compound were thoroughly washed and resuspended with the protein lysis buffer. Then, pull-down samples from immunoprecipitation were assessed by SDS-PAGE and subsequently stained with Fast Silver Stain Kit (Beyotime, Shanghai, China) in accordance with the instructions. And then, the specific strip was cut for mass spectrometry, which was performed at Fitgene corporation (Guangzhou, China). The primer sets applied for RNA transcription in vitro are exhibited in the **Supplementary Table 2** and the antibodies used in the further Western blot analysis are included in the **Supplementary Table 3**.

**Cell counting kit-8 (****CCK-8) and EdU staining and c****olony formation assays**

Cell viability was analyzed with the CCK-8 kit (Glpbio, CA, USA) in conformity to the manufacturer’s recommendations. Concisely, 2.0 × 10^3^ cells (per well) were seeded in 96-well plates and the culture medium of each well was replaced by ten microliters of CCK-8 solution mixed in 100 ul fresh culture medium in a certain time of culturing, subsequently incubated for 2 hours in the incubator. Finally, the absorbance of each well, representing the viability of cells, was investigated at 450 nm with a microplate spectrophotometer (BioTek, VT, USA).

EdU staining assays were performed with EdU Cell Proliferation Kit with Alexa Fluor 555 (Beyotime, Shanghai, China) and cell viability was calculated by the EdU staining positive rate in conformity to the instructions of the kit. Succinctly, cells were seeded in 24-well plates and cultured until the logarithmic growth phase. Additionally, EdU (5-ethynyl-2' -deoxyuridine) solution was added to the culture medium of each well and incubated for 2 hours. The cells were fixed by 4% paraformaldehyde for 15 min after the medium were removed and the proliferative cells were stained by EdU click additive solution while the cell nucleic was stained by Hoechst solution, which were imaged by a fluorescence microscopy (Olympus, Tokyo, Japan).

For colony formation assays, cells were planted into 6-well plates at 1000 cells/well and cultured at 37°C in the incubator for 14 days. Ulteriorly, cells were fixed with methanol for 25 min after removing the culture medium and stained with 0.1% crystal violet. Eventually, the cell colonies (diameters greater than 100 um) were counted by ImageJ software^13^.

**In vitro transwell assays and wound-healing assay**

The 8-μm pore transwell chambers without or with a Matrigel matrix (Yeasen Biotechnology Co., Shanghai, China) were utilized to explore cell ability of migration and invasion in vitro, respectively. 3 × 10^4^ cells were gained in 200 ul of serum-free medium and planted onto the upper chamber, which was either precoated with 100 ul of Matrigel to assess invasion ability or left uncoated to evaluate migration ability of TNBC cells. Meanwhile, 700 ul of medium with 20% fetal bovine serum was provided to the lower chamber as chemo-attractant. After incubation for 24 hours in the incubator, the migrating or invading cells through the filter were fixed with 100% methanol and dyed with 0.1% crystal violet and the number of cells was calculated by ImageJ software.

To assess cell motility, the wound-healing assay was executed, which was to generate wounds in monolayers of cells with assist of the 200-ul pipette tip. Moreover, the culture medium was substituted by serum-free medium, followed by imaging the scratches in 0 or 24 hours by live-cell analysis microscopy (Olympus, Tokyo, Japan).

**Immunohistochemical (IHC) analysis and** **Hematoxylin-eosin (HE) staining**

In general, all dissected mice tissues for staining with HE or IHC analysis were formalin-fixed and paraffin-embedded as previously described^11^. For IHC analysis, the sections were initially dewaxed and sequentially hydrated, subsequently recovering antigens and eliminating endogenous enzymes by using Universal two-step test kit (ZSGB Bio., Beijing, China) and incubated with antibodies against hnRNPK, β-catenin, Ki-67, E-cadherin, N-cadherin, Vimentin and ZEB1 (shown at Supplementary Table 3) overnight at 4°C and with secondary antibodies for 30 min at room temperature. And the sections continued to visualizing antigen by DAB solution, followed by counterstaining with Mayer’s hematoxylin. Image quantification was completed utilizing the HALO Image Analysis Software (HALO v3.3, Indica labs).

For HE staining, after dewaxed and hydrated, the sections were dipped in hematoxylin for 5 min and washed gently with running tap water for 30 min. Next, the sections were dyed with eosin for 3 min. All the stained slides were scanned and imaged by an auto scan machine (KFBIO, Ningbo, China).

**Promoter dual-luciferase reporter assay and** **TOP/FOP‑flash luciferase reporter analysis**

To confirm that FOXO1 could bind the promoter of LYPLAL1-DT and regulate its expression, 293T cells were transiently co-transfected with the plasmid pEZX-FR01 inserted the sequences of the promoter of LYPLAL1-DT and plasmid pcDNA-3.1 overexpressing FOXO1 or siRNA knock-downing FOXO1 or the corresponding controls including empty vector and NT siRNA. Completing incubation for 48 h, the luciferase activity was assessed with the Dual-Luciferase Reporter Assay Kit (Promega, WI, USA) in accord with the manufacturer’s protocols.

TOP/FOP‑flash luciferase reporter assay was employed to evaluate the transcriptional activity of Wnt/β-catenin signaling pathway. Transient co-transfections were accomplished in 293T cells with the plasmid pRL-SV40 and plasmid TOP-FLASH or FOP-FLASH, and after 24 h incubation, luciferase activity was investigated with the Dual-Luciferase Reporter Assay Kit. The relative luciferase activity of TOP/FOP was regarded as the symbol of β-catenin-mediated transcriptional activity.

**References**

1 Tang, L. *et al.* Long non-coding RNA MIR200CHG promotes breast cancer proliferation, invasion, and drug resistance by interacting with and stabilizing YB-1. *NPJ Breast Cancer* **7**, 94 (2021).

2 Han, Y. J. *et al.* LncRNA BLAT1 is Upregulated in Basal-like Breast Cancer through Epigenetic Modifications. *Sci Rep* **8**, 15572 (2018).

3 Chandrashekar, D. S. *et al.* UALCAN: A Portal for Facilitating Tumor Subgroup Gene Expression and Survival Analyses. *Neoplasia* **19**, 649-658 (2017).

4 Ritchie, M. E. *et al.* limma powers differential expression analyses for RNA-sequencing and microarray studies. *Nucleic Acids Res* **43**, e47 (2015).

5 Kang, Y. J. *et al.* CPC2: a fast and accurate coding potential calculator based on sequence intrinsic features. *Nucleic Acids Res* **45**, W12-W16 (2017).

6 Lin, M. F., Jungreis, I. & Kellis, M. PhyloCSF: a comparative genomics method to distinguish protein coding and non-coding regions. *Bioinformatics* **27**, i275-282 (2011).

7 Lanczky, A. & Gyorffy, B. Web-Based Survival Analysis Tool Tailored for Medical Research (KMplot): Development and Implementation. *J Med Internet Res* **23**, e27633 (2021).

8 Sherman, B. T. *et al.* DAVID: a web server for functional enrichment analysis and functional annotation of gene lists (2021 update). *Nucleic Acids Res*, doi:10.1093/nar/gkac194 (2022).

9 Huang da, W., Sherman, B. T. & Lempicki, R. A. Systematic and integrative analysis of large gene lists using DAVID bioinformatics resources. *Nat Protoc* **4**, 44-57 (2009).

10 Subramanian, A. *et al.* Gene set enrichment analysis: a knowledge-based approach for interpreting genome-wide expression profiles. *Proc Natl Acad Sci U S A* **102**, 15545-15550 (2005).

11 Zheng, S. *et al.* Long non-coding RNA HUMT hypomethylation promotes lymphangiogenesis and metastasis via activating FOXK1 transcription in triple-negative breast cancer. *J Hematol Oncol* **13**, 17 (2020).

12 Liu, P. *et al.* The FUS/circEZH2/KLF5/ feedback loop contributes to CXCR4-induced liver metastasis of breast cancer by enhancing epithelial-mesenchymal transition. *Mol Cancer* **21**, 198 (2022).

13 Schindelin, J. *et al.* Fiji: an open-source platform for biological-image analysis. *Nat Methods* **9**, 676-682 (2012).

**Supplementary Tables**

**Supplementary Table 1.** siRNAs, ASOs and Cy3-labled LYPLAL1-DT probes sequence

Sequence of siRNAs

| Non-target | siCtrl | 5'-UUCUCCGAACGUGUCACGUTT |
| --- | --- | --- |
|  | hnRNPK-Ctrl | 5'-UGGUUUACAUGUUGUGUGAUU |
| FOXO1 | siFOXO1 #1 | 5'-CCAUGGACAACAACAGUAATT |
|  | siFOXO1 #2 | 5'-GGAGGUAUGAGUCAGUAUATT |
| hnRNPK | sihnRNPK  (hnRNPK-KD) | 5'-GCAUUCUGCUUCAGAGCAATT |

Sequence of mixture containing siRNAs and ASOs to knock down LYPLAL1-DT

| LYPLAL1-DT-KD | siLYPLAL1-DT #1 | 5'-GCUUAAUCUUGCACUUGAU |
| --- | --- | --- |
|  | siLYPLAL1-DT #2 | 5'-CACACCAUGUUUACUAGUA |
|  | siLYPLAL1-DT #3 | 5'-CAUCCAAACUUAAGUGAAA |
|  | ASO #1 | 5'-AAACC GCAAGGAACC UGGGA |
|  | ASO #2 | 5'-GCCAU TGGGCCTTGA GUGAA |
|  | ASO #3 | 5'-CAGAA AACTTACTTC CUAGC |

Sequence of Cy3-labled LYPLAL1-DT probes for RNA-FISH

| Cy3-labled LYPLAL1-DT probes | Target sequence of LYPLAL1-DT #1 | 5'- gctgacacacgtagaatacc |
| --- | --- | --- |
|  | Target sequence of LYPLAL1-DT #2 | 5'- ccttaacactagcaagaggc |
|  | Target sequence of LYPLAL1-DT #3 | 5'- caacagctgtcagatcccag |
| Cy3-labled 18S probes | Target sequence of 18S | 5'-CTTCCTTGGATGTGGTAGCCGTTTC |
| Cy3-labled U6 probes | Target sequence of U6 | 5'-TTTGCGTGTCATCCTTGCG |

**Supplementary Table 2.** Primers sequence

Primers for RT-qPCR detection

| LYPLAL1-DT | Forward | GCCAGGCAGTACTTGTCTCA |
| --- | --- | --- |
|  | Reverse | CTGAGCTGGCAATCAAGTGC |
| β-actin | Forward | CATGTACGTTGCTATCCAGGC |
|  | Reverse | CTCCTTAATGTCACGCACGAT |
| β-catenin | Forward | ACAGGGAAGACATCACTGAGCC |
|  | Reverse | CAGTGGGATGGTGGGTGTAAGA |
| GAPDH | Forward | ACAACTTTGGTATCGTGGAAGG |
|  | Reverse | GCCATCACGCCACAGTTTC |
| U6 | Forward | CTCGCTTCGGCAGCACA |
|  | Reverse | AACGCTTCACGAATTTGCGT |
| FOXO1 | Forward | TCGTCATAATCTGTCCCTACACA |
|  | Reverse | CGGCTTCGGCTCTTAGCAAA |
| cyclin D1 | Forward | GCTGCGAAGTGGAAACCATC |
|  | Reverse | CCTCCTTCTGCACACATTTGAA |
| c-Myc | Forward | GGCTCCTGGCAAAAGGTCA |
|  | Reverse | CTGCGTAGTTGTGCTGATGT |
| Met | Forward | GGTTCACTGCATATTCTCCCC |
|  | Reverse | ACCATCTTTCGTTTCCTTTAGCC |
| c-Jun | Forward | TCCAAGTGCCGAAAAAGGAAG |
|  | Reverse | CGAGTTCTGAGCTTTCAAGGT |

Primers for RT-qPCR after ChIP.

| -1115 ~ -971bp (site #1) | Forward | ATGCACCGTCTTGTGATCTT |
| --- | --- | --- |
|  | Reverse | CTTGGCCCAGGGTAAGCTC |
| -597 ~ -486bp (site #2) | Forward | GCAATGGGCAATTACGACTG |
|  | Reverse | CCCCAAGTTTAAACAGAGCA |
| -178 ~ -25bp (site #3) | Forward | AAGATCAGAGAGGCGCTAT |
|  | Reverse | CGTGCCTGCGTATTTGC |

Primers for in vivo transcribed RNA with biotinylating

| LYPLAL1-DT Sense | Forward | taatacgactcactataGggATGCGCAGCGCTGCTGGC |
| --- | --- | --- |
|  | Reverse | TCATGTATATTATCTAATTTAATTCTCATTACAATCCTGTGAAGTACA |
| LYPLAL1-DT Anti-sense | Forward | taatacgactcactataGggTCATGTATATTATCTAATTTAATTCTCAT |
|  | Reverse | ATGCGCAGCGCTGCTGGC |
| Exon 5 | Forward | taatacgactcactataGggGCCTTGCCTCCTGGATGGCATTTTTGAACCCACCC |
|  | Reverse | TCATGTATATTATCTAATTTAATTCTCATTACAATCCTGTGAAGTACA |
| Exon 1+2+3+4 | Forward | taatacgactcactataGggATGCGCAGCGCTGCTGGC |
|  | Reverse | CTTCTGTTCCTCCTCCTTCCTCACAGGCATTCCTC |
| Exon 1+2+3 | Forward | taatacgactcactataGggATGCGCAGCGCTGCTGGC |
|  | Reverse | CTTCTGTTCCTCCTCCTTCCTCACAGGCATTCCTC |
| Exon 1+2 | Forward | taatacgactcactataGggATGCGCAGCGCTGCTGGC |
|  | Reverse | AGCAAGAGGCTTTTTTCCCTTGCGTAGCATACTTC |
| Exon 1 | Forward | taatacgactcactataGggATGCGCAGCGCTGCTGGC |
|  | Reverse | CCTGTATATCAAGAGCTGCAAACATCCTGTGCGAG |

**Supplementary Table 3.** Antibodies used in this study.

| Antibody | Cat No; Source | Application |
| --- | --- | --- |
| FOXO1 | 18592-1-AP; Proteintech | 1:1000 for WB; 5 ul for ChIP and IP |
| GAPDH | 60004-1-Ig; Proteintech | 1:20000 for WB |
| IgG | 30000-0-AP; Proteintech | 5 ul for ChIP, IP and RIP |
| β-catenin | #8480; Cell Signaling Technology | 1:1000 for WB; 1:50 for RIP and IP; 1: 100 for IF; 1:100 for IHC |
| hnRNPK | 11426-1-AP; Proteintech | 1:2000 for WB; 5 ul for RIP and IP; 1:100 for IHC |
| hnRNPK | 67708-1-Ig; Proteintech | 1:2000 for WB; 1:50 for IF |
| E-cadherin | #3195; Cell Signaling Technology | 1:500 for WB; 1:400 for IHC |
| N-cadherin | #13116; Cell Signaling Technology | 1:500 for WB; 1:100 for IHC |
| Vimentin | #5741; Cell Signaling Technology | 1:1000 for WB; 1:200 for IHC |
| SLUG | #9585; Cell Signaling Technology | 1:500 for WB |
| ZEB1 | #70512; Cell Signaling Technology | 1:1000 for WB; 1:500 for IHC |
| cyclin D1 | 60186-1-Ig; Proteintech | 1:2000 for WB |
| c-Myc | 10828-1-AP; Proteintech | 1:1000 for WB |
| Met | #8198; Cell Signaling Technology | 1:1000 for WB |
| c-Jun | 24909-1-AP; Proteintech | 1:1000 for WB |
| β-actin | 81115-1-RR; Proteintech | 1:5000 for WB |
| β-tubulin | 10068-1-AP; Proteintech | 1:1000 for WB |
| H3 histone | 17168-1-AP; Proteintech | 1:1000 for WB |
| myc tag | 16286-1-AP; Proteintech | 5 ul for IP |
| myc tag | 60003-2-Ig; Proteintech | 1:1000 for WB |
| HA tag | 66006-2-Ig; Proteintech | 1:1000 for WB |
| Ki-67 | #9449; Cell Signaling Technology | 1: 1000 for IHC |
| Anti-Rabbit IgG (H+L), HRP Conjugate | bs-0295G; Bioss | 1:5000 for WB |
| Anti-Mouse IgG (H+L), HRP Conjugate | bs-0296G; Bioss | 1:5000 for WB |
| CoraLite594-conjugated Goat Anti-Rabbit IgG (H+L) | SA00013-4; Proteintech | 1:100 for IF |
| CoraLite488-conjugated Goat Anti-Rabbit IgG (H+L) | SA00013-1; Proteintech | 1:100 for IF |

| **Supplementary Table 4**. TFs significantly correlated with LYPLAL1-DT | | |
| --- | --- | --- |
| TFs | Pearson *r* | *p* value |
| ZNF641 | 0.494 | 3.27E-11 |
| ZFP14 | 0.486 | 7.31E-11 |
| ZNF529 | 0.475 | 2.14E-10 |
| ZNF25 | 0.468 | 4.43E-10 |
| ZNF461 | 0.467 | 4.95E-10 |
| ZNF260 | 0.457 | 1.30E-09 |
| ZNF383 | 0.453 | 1.84E-09 |
| NR1D2 | 0.45 | 2.36E-09 |
| ZNF292 | 0.438 | 6.94E-09 |
| ZNF333 | 0.433 | 1.05E-08 |
| ZBTB11 | 0.43 | 1.44E-08 |
| ZNF565 | 0.424 | 2.25E-08 |
| NFATC3 | 0.424 | 2.32E-08 |
| ADNP | 0.422 | 2.73E-08 |
| BBX | 0.421 | 2.99E-08 |
| ZNF669 | 0.421 | 3.06E-08 |
| PLAG1 | 0.421 | 3.06E-08 |
| ZNF678 | 0.419 | 3.38E-08 |
| ZNF33A | 0.419 | 3.57E-08 |
| CTCF | 0.418 | 3.65E-08 |
| CARF | 0.418 | 3.66E-08 |
| ZBTB24 | 0.417 | 4.20E-08 |
| GTF2IRD2B | 0.416 | 4.36E-08 |
| ZNF585B | 0.416 | 4.50E-08 |
| ZNF566 | 0.415 | 4.91E-08 |
| ZNF567 | 0.414 | 5.36E-08 |
| KMT2B | 0.411 | 6.73E-08 |
| HBP1 | 0.408 | 8.47E-08 |
| GTF2IRD2 | 0.404 | 1.19E-07 |
| ZNF148 | 0.402 | 1.35E-07 |
| USF3 | 0.402 | 1.41E-07 |
| KDM2A | 0.397 | 1.98E-07 |
| ASH1L | 0.394 | 2.46E-07 |
| ZNF780A | 0.392 | 2.89E-07 |
| BAZ2B | 0.392 | 2.95E-07 |
| MYNN | 0.391 | 3.13E-07 |
| VEZF1 | 0.391 | 3.20E-07 |
| ZNF420 | 0.391 | 3.22E-07 |
| ZNF792 | 0.39 | 3.35E-07 |
| ATF6B | 0.39 | 3.35E-07 |
| NFAT5 | 0.389 | 3.68E-07 |
| ZNF827 | 0.389 | 3.76E-07 |
| ZNF518A | 0.389 | 3.80E-07 |
| ZNF570 | 0.388 | 3.88E-07 |
| TERF2 | 0.388 | 4.11E-07 |
| ZNF439 | 0.387 | 4.23E-07 |
| ELF2 | 0.385 | 4.84E-07 |
| GTF2I | 0.385 | 5.07E-07 |
| MEF2D | 0.382 | 6.05E-07 |
| ZFP30 | 0.382 | 6.14E-07 |
| REST | 0.381 | 6.55E-07 |
| KLF3 | 0.381 | 6.58E-07 |
| ZNF568 | 0.381 | 6.68E-07 |
| THAP9 | 0.38 | 7.14E-07 |
| ZNF585A | 0.38 | 7.31E-07 |
| ZNF181 | 0.379 | 7.55E-07 |
| ZNF708 | 0.379 | 8.00E-07 |
| ZFP91 | 0.378 | 8.12E-07 |
| ZKSCAN1 | 0.378 | 8.20E-07 |
| CREB1 | 0.378 | 8.27E-07 |
| ZNF345 | 0.376 | 9.42E-07 |
| ZNF780B | 0.376 | 9.44E-07 |
| ZFP90 | 0.376 | 9.57E-07 |
| TRPS1 | 0.375 | 9.97E-07 |
| ZHX1 | 0.375 | 1.01E-06 |
| ZNF322 | 0.375 | 1.04E-06 |
| NCOA2 | 0.374 | 1.13E-06 |
| SKIL | 0.374 | 1.14E-06 |
| ZSCAN25 | 0.373 | 1.20E-06 |
| ZXDC | 0.373 | 1.21E-06 |
| ZNF526 | 0.372 | 1.24E-06 |
| KDM5B | 0.372 | 1.28E-06 |
| ELF1 | 0.371 | 1.39E-06 |
| ZNF670 | 0.37 | 1.50E-06 |
| ZNF721 | 0.368 | 1.66E-06 |
| THAP5 | 0.368 | 1.68E-06 |
| FOXO4 | 0.367 | 1.77E-06 |
| ZFP1 | 0.367 | 1.82E-06 |
| ZKSCAN3 | 0.367 | 1.84E-06 |
| ATF6 | 0.366 | 1.90E-06 |
| SRCAP | 0.365 | 2.03E-06 |
| MSANTD4 | 0.365 | 2.08E-06 |
| AHCTF1 | 0.363 | 2.35E-06 |
| MBNL2 | 0.363 | 2.42E-06 |
| ZBTB37 | 0.363 | 2.43E-06 |
| ATF2 | 0.363 | 2.43E-06 |
| PRDM2 | 0.362 | 2.55E-06 |
| ZNF146 | 0.362 | 2.62E-06 |
| ZNF790 | 0.36 | 3.02E-06 |
| ZNF304 | 0.359 | 3.12E-06 |
| SPEN | 0.359 | 3.12E-06 |
| FOXR2 | 0.359 | 3.13E-06 |
| ZNF658 | 0.358 | 3.34E-06 |
| ETV3 | 0.358 | 3.43E-06 |
| ZNF143 | 0.357 | 3.48E-06 |
| SP1 | 0.355 | 3.99E-06 |
| ZNF184 | 0.355 | 4.06E-06 |
| KAT7 | 0.355 | 4.10E-06 |
| BCL6 | 0.355 | 4.17E-06 |
| ZNF599 | 0.354 | 4.29E-06 |
| NFE2L1 | 0.354 | 4.41E-06 |
| TFDP2 | 0.354 | 4.42E-06 |
| NFX1 | 0.353 | 4.57E-06 |
| SP3 | 0.353 | 4.69E-06 |
| ATMIN | 0.353 | 4.80E-06 |
| SON | 0.352 | 4.91E-06 |
| ZBTB49 | 0.352 | 5.00E-06 |
| THAP6 | 0.351 | 5.27E-06 |
| ZNF718 | 0.351 | 5.31E-06 |
| RXRB | 0.351 | 5.36E-06 |
| ZNF207 | 0.351 | 5.47E-06 |
| PRDM10 | 0.35 | 5.61E-06 |
| ZNF37A | 0.349 | 5.96E-06 |
| ZNF302 | 0.349 | 6.08E-06 |
| ZNF561 | 0.349 | 6.14E-06 |
| ZNF655 | 0.348 | 6.42E-06 |
| ZNF30 | 0.347 | 6.76E-06 |
| ELK4 | 0.347 | 6.96E-06 |
| ZBTB41 | 0.347 | 6.99E-06 |
| CIC | 0.346 | 7.30E-06 |
| UBP1 | 0.346 | 7.30E-06 |
| ZBTB38 | 0.346 | 7.52E-06 |
| ZNF605 | 0.345 | 7.85E-06 |
| ZNF548 | 0.345 | 7.90E-06 |
| ZNF14 | 0.344 | 8.37E-06 |
| ZBTB3 | 0.343 | 8.80E-06 |
| PRDM4 | 0.343 | 9.20E-06 |
| ZNF607 | 0.342 | 9.56E-06 |
| ZNF449 | 0.342 | 9.72E-06 |
| ZNF501 | 0.342 | 9.80E-06 |
| PRDM5 | 0.342 | 9.85E-06 |
| ZFP82 | 0.339 | 1.14E-05 |
| REL | 0.339 | 1.18E-05 |
| ZNF644 | 0.339 | 1.19E-05 |
| AHDC1 | 0.339 | 1.19E-05 |
| ZNF654 | 0.338 | 1.20E-05 |
| ATF7 | 0.338 | 1.21E-05 |
| GPBP1 | 0.338 | 1.26E-05 |
| NFIB | 0.337 | 1.33E-05 |
| POU2F1 | 0.336 | 1.41E-05 |
| ZIC4 | 0.335 | 1.46E-05 |
| FOXN2 | 0.335 | 1.46E-05 |
| ZNF197 | 0.335 | 1.53E-05 |
| KLF11 | 0.333 | 1.70E-05 |
| THAP2 | 0.332 | 1.76E-05 |
| HIVEP1 | 0.332 | 1.83E-05 |
| CGGBP1 | 0.33 | 2.03E-05 |
| TIGD7 | 0.33 | 2.06E-05 |
| SP2 | 0.329 | 2.17E-05 |
| ZBTB5 | 0.329 | 2.21E-05 |
| ZNF774 | 0.329 | 2.23E-05 |
| GATAD2B | 0.328 | 2.23E-05 |
| ZNF451 | 0.328 | 2.26E-05 |
| GABPA | 0.328 | 2.35E-05 |
| CREB3L2 | 0.326 | 2.55E-05 |
| ZNF623 | 0.325 | 2.70E-05 |
| BAZ2A | 0.325 | 2.71E-05 |
| ZKSCAN4 | 0.324 | 2.92E-05 |
| ZNF267 | 0.324 | 2.94E-05 |
| HMG20A | 0.324 | 2.95E-05 |
| CEBPG | 0.324 | 2.98E-05 |
| ZNF471 | 0.324 | 3.00E-05 |
| ZNF431 | 0.323 | 3.06E-05 |
| ZNF41 | 0.323 | 3.09E-05 |
| ZXDB | 0.323 | 3.13E-05 |
| ZNF493 | 0.323 | 3.16E-05 |
| GPBP1L1 | 0.323 | 3.16E-05 |
| ZNF35 | 0.323 | 3.18E-05 |
| ZBTB26 | 0.323 | 3.19E-05 |
| PPARA | 0.322 | 3.35E-05 |
| ZNF845 | 0.322 | 3.38E-05 |
| ZNF169 | 0.321 | 3.57E-05 |
| PBX2 | 0.32 | 3.79E-05 |
| ZBTB14 | 0.319 | 3.85E-05 |
| ZNF140 | 0.319 | 3.91E-05 |
| ZNF507 | 0.319 | 3.98E-05 |
| ZNF888 | 0.319 | 4.05E-05 |
| GRHL2 | 0.318 | 4.09E-05 |
| NFIX | 0.318 | 4.23E-05 |
| ZNF830 | 0.316 | 4.58E-05 |
| ZNF326 | 0.316 | 4.65E-05 |
| RLF | 0.316 | 4.68E-05 |
| DR1 | 0.316 | 4.73E-05 |
| ZNF266 | 0.316 | 4.73E-05 |
| ZNF496 | 0.316 | 4.76E-05 |
| ZSCAN32 | 0.316 | 4.77E-05 |
| ZBTB44 | 0.316 | 4.81E-05 |
| ATF1 | 0.315 | 4.89E-05 |
| ZHX2 | 0.314 | 5.18E-05 |
| PURB | 0.314 | 5.35E-05 |
| TMF1 | 0.313 | 5.65E-05 |
| ZNF430 | 0.313 | 5.68E-05 |
| ZNF778 | 0.312 | 5.76E-05 |
| ZNF791 | 0.311 | 6.28E-05 |
| ZBED5 | 0.311 | 6.34E-05 |
| ZSCAN30 | 0.31 | 6.77E-05 |
| ZNF429 | 0.309 | 6.91E-05 |
| AEBP2 | 0.309 | 6.92E-05 |
| ZNF621 | 0.309 | 7.02E-05 |
| NRF1 | 0.307 | 7.64E-05 |
| ZXDA | 0.307 | 7.65E-05 |
| NCOA1 | 0.307 | 7.70E-05 |
| ZBTB22 | 0.307 | 7.84E-05 |
| TCF20 | 0.307 | 7.92E-05 |
| ZBTB18 | 0.307 | 8.02E-05 |
| TOPORS | 0.307 | 8.03E-05 |
| ZFP3 | 0.306 | 8.15E-05 |
| ZNF112 | 0.306 | 8.34E-05 |
| TET3 | 0.306 | 8.34E-05 |
| TIGD2 | 0.306 | 8.43E-05 |
| ARNT | 0.305 | 8.66E-05 |
| OTX1 | 0.305 | 8.69E-05 |
| ZKSCAN2 | 0.305 | 8.79E-05 |
| POU5F1 | 0.305 | 8.94E-05 |
| ZSCAN20 | 0.304 | 9.48E-05 |
| ZSCAN26 | 0.302 | 0.000105886 |
| TBX4 | 0.302 | 0.000106612 |
| RBPJ | 0.301 | 0.000109723 |
| ZNF525 | 0.301 | 0.000110913 |
| ZNF836 | 0.301 | 0.000111796 |
| PRDM15 | 0.3 | 0.000113027 |
| NR2C2 | 0.3 | 0.000114963 |
| ZNF541 | 0.299 | 0.000122255 |
| FOXO3 | 0.299 | 0.000125435 |
| TEAD3 | 0.298 | 0.000126968 |
| ZNF543 | 0.298 | 0.000129022 |
| CEBPZ | 0.298 | 0.000132204 |
| ZNF627 | 0.298 | 0.000132525 |
| ZNF28 | 0.298 | 0.000132671 |
| ZNF319 | 0.297 | 0.000133828 |
| ZNF765 | 0.297 | 0.000135906 |
| ZNF470 | 0.297 | 0.00014 |
| STAT3 | 0.295 | 0.000150646 |
| SP100 | 0.295 | 0.000154787 |
| RFX3 | 0.295 | 0.000155133 |
| ZNF664 | 0.294 | 0.000157831 |
| JRKL | 0.294 | 0.000158916 |
| ZNF546 | 0.294 | 0.000158978 |
| ZNF512 | 0.294 | 0.000164816 |
| KDM2B | 0.292 | 0.000178542 |
| ZNF343 | 0.292 | 0.000180307 |
| ZKSCAN8 | 0.292 | 0.000182687 |
| ZNF770 | 0.291 | 0.000186841 |
| IKZF4 | 0.29 | 0.0001948 |
| ZBTB1 | 0.29 | 0.00019587 |
| ZNF829 | 0.29 | 0.000197097 |
| THRA | 0.29 | 0.000200026 |
| RARG | 0.29 | 0.000201772 |
| ZNF268 | 0.289 | 0.000213798 |
| ZNF592 | 0.288 | 0.000216929 |
| ZBTB6 | 0.288 | 0.000217317 |
| ZNF646 | 0.288 | 0.000221708 |
| ZNF558 | 0.287 | 0.000228444 |
| ZNF594 | 0.287 | 0.000235382 |
| PHF21A | 0.287 | 0.000237256 |
| ZNF19 | 0.287 | 0.000239097 |
| ZNF280D | 0.287 | 0.000239457 |
| ZNF700 | 0.286 | 0.000243006 |
| ZNF318 | 0.285 | 0.000255731 |
| ZNF273 | 0.285 | 0.000257977 |
| ZNF382 | 0.285 | 0.00026142 |
| SETDB2 | 0.285 | 0.00026152 |
| SMAD4 | 0.285 | 0.000263486 |
| DPF3 | 0.285 | 0.000264649 |
| ZNF69 | 0.283 | 0.000286426 |
| ZBTB4 | 0.283 | 0.000293065 |
| ZNF675 | 0.283 | 0.00029347 |
| ZNF438 | 0.282 | 0.000296591 |
| ZNF454 | 0.282 | 0.000298709 |
| EHF | 0.282 | 0.000300413 |
| ZNF572 | 0.282 | 0.000303544 |
| MGA | 0.282 | 0.000304784 |
| ZNF740 | 0.282 | 0.000309495 |
| ZNF490 | 0.281 | 0.000321519 |
| ZFP64 | 0.281 | 0.000325946 |
| ZNF783 | 0.28 | 0.000334694 |
| ZNF611 | 0.28 | 0.000335372 |
| ZNF619 | 0.28 | 0.000340379 |
| ZNF182 | 0.28 | 0.000342705 |
| ZNF136 | 0.279 | 0.000344715 |
| ZBTB34 | 0.279 | 0.000351692 |
| ZBTB33 | 0.279 | 0.000354809 |
| ZNF879 | 0.278 | 0.000363344 |
| ZNF155 | 0.278 | 0.000363783 |
| BCL11A | 0.278 | 0.000365093 |
| ZNF684 | 0.278 | 0.0003662 |
| ZNF426 | 0.278 | 0.000367527 |
| ZNF396 | 0.277 | 0.000386241 |
| ZNF83 | 0.277 | 0.000390844 |
| ZNF251 | 0.277 | 0.000393572 |
| ZNF397 | 0.276 | 0.000406581 |
| ZNF280B | 0.276 | 0.000411718 |
| ZNF174 | 0.276 | 0.000414245 |
| ZNF417 | 0.275 | 0.00042389 |
| ZNF441 | 0.275 | 0.000430536 |
| CDC5L | 0.275 | 0.000437902 |
| IRF2 | 0.274 | 0.000447623 |
| ZNF711 | 0.274 | 0.000447628 |
| ZBTB40 | 0.274 | 0.000448015 |
| ZNF527 | 0.273 | 0.000466728 |
| ZNF70 | 0.273 | 0.000469306 |
| ZNF766 | 0.273 | 0.000469524 |
| CSRNP2 | 0.273 | 0.0004724 |
| ZNF248 | 0.272 | 0.000499741 |
| ZNF562 | 0.272 | 0.000504792 |
| ZNF142 | 0.272 | 0.000506666 |
| FOXN3 | 0.272 | 0.000510742 |
| FOXJ2 | 0.271 | 0.000517913 |
| KLF13 | 0.271 | 0.000530167 |
| SATB1 | 0.271 | 0.000533689 |
| ZNF445 | 0.271 | 0.000538966 |
| ZNF462 | 0.27 | 0.000547961 |
| ZSCAN21 | 0.27 | 0.000553833 |
| ZNF557 | 0.27 | 0.000558953 |
| ZZZ3 | 0.27 | 0.000564198 |
| ZNF75A | 0.269 | 0.000577062 |
| ZNF347 | 0.269 | 0.000592574 |
| MEF2A | 0.269 | 0.000592688 |
| IRF6 | 0.268 | 0.000613456 |
| MYT1L | 0.268 | 0.000616654 |
| ZNF23 | 0.267 | 0.000639116 |
| GLMP | 0.267 | 0.000639607 |
| STAT5B | 0.266 | 0.000664358 |
| ZFP28 | 0.266 | 0.000666317 |
| ZNF26 | 0.266 | 0.00067144 |
| TCF12 | 0.266 | 0.000675234 |
| ZNF160 | 0.266 | 0.000681887 |
| BPTF | 0.265 | 0.000702568 |
| ZNF281 | 0.265 | 0.000717081 |
| ZFP69B | 0.265 | 0.000720987 |
| ZNF821 | 0.265 | 0.000724066 |
| AC092835.1 | 0.264 | 0.000753908 |
| ZBTB25 | 0.264 | 0.000754414 |
| TET2 | 0.264 | 0.000757539 |
| ZNF320 | 0.264 | 0.000760367 |
| ARID5B | 0.263 | 0.000761854 |
| ZNF763 | 0.263 | 0.000783249 |
| BACH2 | 0.263 | 0.000785349 |
| SP4 | 0.263 | 0.000789233 |
| SOX6 | 0.263 | 0.000791051 |
| DMTF1 | 0.262 | 0.000803323 |
| ZNF521 | 0.262 | 0.000806285 |
| ZBTB39 | 0.262 | 0.000823254 |
| STAT2 | 0.262 | 0.000833516 |
| NR3C2 | 0.261 | 0.000842278 |
| EEA1 | 0.26 | 0.000881071 |
| PURA | 0.26 | 0.000881906 |
| ZNF781 | 0.26 | 0.000899542 |
| ZNF92 | 0.26 | 0.000915872 |
| FERD3L | 0.26 | 0.000919159 |
| ZNF391 | 0.26 | 0.000919932 |
| ZNF880 | 0.259 | 0.000940443 |
| ZNF785 | 0.259 | 0.000948402 |
| C11orf95 | 0.259 | 0.000951296 |
| ZNF212 | 0.258 | 0.000968235 |
| TTF1 | 0.258 | 0.000982357 |
| MXI1 | 0.258 | 0.000991486 |
| ZFP37 | 0.258 | 0.001008706 |
| ZNF540 | 0.257 | 0.001035779 |
| KMT2A | 0.257 | 0.001055797 |
| MTERF2 | 0.256 | 0.001081536 |
| ZC3H8 | 0.256 | 0.001089034 |
| ZNF263 | 0.256 | 0.001096093 |
| ZNF805 | 0.256 | 0.001108498 |
| ZNF135 | 0.255 | 0.001112328 |
| ZNF17 | 0.255 | 0.001117484 |
| ZBTB43 | 0.255 | 0.001144614 |
| ZNF550 | 0.255 | 0.001146011 |
| MXD1 | 0.254 | 0.001164907 |
| ZNF547 | 0.254 | 0.001199132 |
| ARNT2 | 0.253 | 0.001224792 |
| ZNF217 | 0.253 | 0.001230621 |
| ZNF264 | 0.253 | 0.001240212 |
| GMEB1 | 0.252 | 0.001278682 |
| ZNF813 | 0.252 | 0.001300764 |
| ZBTB8A | 0.252 | 0.001302705 |
| ZNF860 | 0.252 | 0.001303183 |
| POGK | 0.252 | 0.001309557 |
| ZNF3 | 0.252 | 0.001323039 |
| ZNF555 | 0.252 | 0.001328652 |
| GZF1 | 0.252 | 0.001329667 |
| BACH1 | 0.251 | 0.001338715 |
| LIN54 | 0.251 | 0.001349781 |
| ZNF506 | 0.251 | 0.001351927 |
| STAT6 | 0.251 | 0.001364064 |
| ZNF141 | 0.251 | 0.001366047 |
| ZIC1 | 0.251 | 0.001373613 |
| ZNF843 | 0.251 | 0.001376408 |
| ZNF45 | 0.251 | 0.001381108 |
| YY1 | 0.251 | 0.001382944 |
| ZNF354C | 0.251 | 0.001390464 |
| DLX6 | 0.25 | 0.001414026 |
| ZNF7 | 0.25 | 0.00141907 |
| ZNF564 | 0.25 | 0.001458198 |
| MYSM1 | 0.249 | 0.001469497 |
| TCFL5 | 0.249 | 0.001476178 |
| TEAD1 | 0.249 | 0.001495754 |
| ZNF101 | 0.248 | 0.00153699 |
| ZNF81 | 0.248 | 0.00158076 |
| ZNF43 | 0.248 | 0.001583651 |
| GLYR1 | 0.247 | 0.001613464 |
| ZNF337 | 0.247 | 0.001622802 |
| ZNF274 | 0.247 | 0.001635137 |
| ZNF8 | 0.247 | 0.001636165 |
| ZNF569 | 0.247 | 0.001668889 |
| PLAGL1 | 0.247 | 0.001671074 |
| ZNF518B | 0.247 | 0.001671217 |
| ZNF687 | 0.246 | 0.001683596 |
| ZNF514 | 0.246 | 0.001723401 |
| ZNF528 | 0.245 | 0.001778902 |
| ZNF253 | 0.245 | 0.001798878 |
| SOX4 | 0.245 | 0.001804495 |
| ZNF84 | 0.244 | 0.001844376 |
| ZNF415 | 0.244 | 0.001881242 |
| ZNF534 | 0.244 | 0.001908423 |
| THAP1 | 0.243 | 0.001927109 |
| ZNF701 | 0.243 | 0.001932953 |
| TRAFD1 | 0.243 | 0.001951197 |
| E2F3 | 0.243 | 0.001972696 |
| PHF1 | 0.243 | 0.001985326 |
| NFE2L2 | 0.243 | 0.001988496 |
| ZNF776 | 0.243 | 0.00199785 |
| MLX | 0.242 | 0.002029104 |
| NFYB | 0.242 | 0.002078314 |
| ETV6 | 0.242 | 0.002086703 |
| ZNF639 | 0.241 | 0.002116925 |
| ZNF667 | 0.24 | 0.002279867 |
| ZNF443 | 0.239 | 0.002288591 |
| ZNF800 | 0.239 | 0.002289742 |
| ZFX | 0.239 | 0.00229079 |
| ZNF629 | 0.239 | 0.002309864 |
| NFKB1 | 0.239 | 0.002377332 |
| ZNF335 | 0.238 | 0.002394952 |
| ZNF699 | 0.238 | 0.002433642 |
| HIVEP2 | 0.238 | 0.002442293 |
| TEF | 0.238 | 0.002454123 |
| PROX1 | 0.238 | 0.002473466 |
| RBSN | 0.238 | 0.00247833 |
| NKX6-1 | 0.237 | 0.002531754 |
| FOXJ3 | 0.237 | 0.002541566 |
| RFX7 | 0.237 | 0.002564169 |
| ZNF600 | 0.237 | 0.002568904 |
| RUNX1 | 0.237 | 0.002580172 |
| PHF20 | 0.237 | 0.00258076 |
| KLF7 | 0.236 | 0.002614502 |
| ZNF624 | 0.236 | 0.002619543 |
| ZKSCAN5 | 0.236 | 0.002690118 |
| NR1I2 | 0.235 | 0.002828185 |
| ZNF510 | 0.234 | 0.002859892 |
| ZNF606 | 0.234 | 0.002877402 |
| ZNF554 | 0.234 | 0.002896261 |
| ZNF2 | 0.234 | 0.002897281 |
| ZBTB7B | 0.234 | 0.002917141 |
| TIGD4 | 0.234 | 0.002959437 |
| ZNF404 | 0.233 | 0.002964695 |
| RFX1 | 0.233 | 0.002970272 |
| FAM200B | 0.233 | 0.002973835 |
| MNT | 0.233 | 0.002981659 |
| IKZF2 | 0.233 | 0.002998012 |
| ZNF436 | 0.233 | 0.003010434 |
| MBD4 | 0.233 | 0.003085698 |
| ZNF717 | 0.232 | 0.003128047 |
| ZNF816 | 0.232 | 0.003155669 |
| FOXL1 | 0.232 | 0.003160777 |
| ZNF79 | 0.232 | 0.003185728 |
| ZNF610 | 0.232 | 0.00318663 |
| ZNF18 | 0.232 | 0.00319772 |
| ZNF24 | 0.231 | 0.003233209 |
| TFAP2C | 0.231 | 0.003234432 |
| CREBL2 | 0.231 | 0.003241399 |
| ZBTB12 | 0.231 | 0.003242904 |
| ZBTB2 | 0.231 | 0.003262718 |
| ALX1 | 0.231 | 0.00326421 |
| ZNF10 | 0.231 | 0.003285882 |
| ZNF473 | 0.23 | 0.003379602 |
| LCOR | 0.23 | 0.003383351 |
| ZNF317 | 0.23 | 0.003414727 |
| ZNF891 | 0.23 | 0.003469693 |
| ZNF574 | 0.23 | 0.003508143 |
| NKX2-3 | 0.229 | 0.003512956 |
| ZNF202 | 0.228 | 0.003659624 |
| ZNF287 | 0.228 | 0.003664601 |
| ZNF615 | 0.228 | 0.003680791 |
| RARB | 0.228 | 0.003751815 |
| CHAMP1 | 0.228 | 0.00377895 |
| ZNF124 | 0.227 | 0.003830474 |
| CREBZF | 0.227 | 0.003896808 |
| ZNF468 | 0.227 | 0.003926353 |
| ZNF761 | 0.227 | 0.003938435 |
| ZMAT1 | 0.226 | 0.004020743 |
| MLXIP | 0.225 | 0.004155076 |
| ZNF793 | 0.225 | 0.004205774 |
| ZNF33B | 0.225 | 0.004259339 |
| ZNF573 | 0.225 | 0.004276675 |
| ZNF724 | 0.224 | 0.004358112 |
| JRK | 0.224 | 0.004415862 |
| ZNF772 | 0.223 | 0.00451553 |
| ZNF665 | 0.223 | 0.004519464 |
| ZNF418 | 0.223 | 0.004557758 |
| TFDP3 | 0.223 | 0.004561334 |
| ZSCAN12 | 0.223 | 0.004577372 |
| GLIS2 | 0.223 | 0.0046163 |
| ZFP62 | 0.222 | 0.004799638 |
| ZNF283 | 0.222 | 0.004810228 |
| THAP11 | 0.221 | 0.004926655 |
| CENPBD1 | 0.221 | 0.004957022 |
| ARID2 | 0.221 | 0.004995506 |
| ZNF787 | -0.221 | 0.005004994 |
| KIN | 0.221 | 0.00501353 |
| ZNF280A | 0.221 | 0.005046072 |
| ZNF469 | 0.221 | 0.005052695 |
| ZNF532 | 0.22 | 0.005117784 |
| ZNF440 | 0.22 | 0.005127563 |
| ZNF12 | 0.22 | 0.005236976 |
| TERF1 | 0.219 | 0.005316369 |
| GSX2 | 0.218 | 0.005525145 |
| POU5F1B | 0.218 | 0.005574296 |
| NR3C1 | 0.218 | 0.005717229 |
| ZNF502 | 0.218 | 0.005727985 |
| ZNF236 | 0.217 | 0.005811315 |
| ZNF44 | 0.217 | 0.005820752 |
| STAT5A | 0.216 | 0.006077113 |
| MTF2 | 0.216 | 0.006170263 |
| ETS1 | 0.216 | 0.006174986 |
| L3MBTL4 | 0.215 | 0.006291922 |
| GRHL1 | 0.215 | 0.006389239 |
| ZNF416 | 0.215 | 0.006396603 |
| TCF7L2 | 0.215 | 0.0063994 |
| ZNF559 | 0.215 | 0.006410637 |
| ZNF275 | 0.214 | 0.006467855 |
| ZNF480 | 0.213 | 0.006757134 |
| ZNF736 | 0.213 | 0.006924271 |
| TFCP2 | 0.213 | 0.006935722 |
| ZNF398 | 0.212 | 0.007141278 |
| PLSCR1 | 0.212 | 0.007190359 |
| NCOA3 | 0.211 | 0.007346557 |
| TIGD1 | 0.211 | 0.007421226 |
| ZNF100 | 0.211 | 0.007438346 |
| ZNF227 | 0.21 | 0.007571033 |
| NOBOX | 0.21 | 0.007680158 |
| MBD6 | 0.21 | 0.007768105 |
| AHRR | 0.21 | 0.007798495 |
| NKX3-2 | 0.209 | 0.007955408 |
| ZNF76 | 0.209 | 0.007966548 |
| KLF5 | 0.209 | 0.008004123 |
| ZNF786 | 0.209 | 0.008038903 |
| GTF2IRD1 | 0.208 | 0.008156748 |
| ZNF131 | 0.208 | 0.008183303 |
| FOXO1 | 0.207 | 0.008551164 |
| MTF1 | 0.207 | 0.008782973 |
| ZNF497 | 0.206 | 0.008866653 |
| ERF | 0.206 | 0.009117611 |
| RELA | 0.206 | 0.00913236 |
| ZNF488 | 0.206 | 0.00913324 |
| HINFP | 0.205 | 0.009141966 |
| ZNF16 | 0.205 | 0.009273381 |
| ZFAT | 0.204 | 0.00950408 |
| ARNTL | 0.204 | 0.009816911 |
| NR2C1 | 0.203 | 0.00990079 |
| ZNF500 | 0.203 | 0.010159896 |
| MTERF4 | 0.202 | 0.010357807 |
| HDX | 0.202 | 0.010587885 |
| ZNF225 | 0.201 | 0.010641821 |
| ZNF230 | 0.201 | 0.010645179 |
| PRMT3 | 0.201 | 0.011013086 |

| **Supplementary Table 5**. TFs down-regulated in TCGA-TNBC | | | |
| --- | --- | --- | --- |
| TFs | logFC | *p* value | adj.*p* value |
| ZBTB16 | -6.034470454 | 9.76E-73 | 1.99E-70 |
| PGR | -5.369403133 | 2.10E-70 | 3.58E-68 |
| FOSB | -4.671500773 | 2.29E-56 | 1.40E-54 |
| ESR1 | -4.461205662 | 6.55E-69 | 9.78E-67 |
| RFX6 | -4.34498752 | 1.69E-45 | 4.91E-44 |
| MLXIPL | -4.254643576 | 4.96E-50 | 1.92E-48 |
| MLXIPL.1 | -4.254643576 | 4.96E-50 | 1.92E-48 |
| MLXIPL.2 | -4.254643576 | 4.96E-50 | 1.92E-48 |
| ZNF728 | -3.951910124 | 5.61E-64 | 5.75E-62 |
| TP63 | -3.905075769 | 1.44E-36 | 2.39E-35 |
| ZNF676 | -3.852642685 | 7.00E-56 | 4.13E-54 |
| ATOH8 | -3.791578195 | 3.53E-67 | 4.61E-65 |
| RXRG | -3.753166259 | 1.80E-46 | 5.55E-45 |
| DACH1 | -3.751063031 | 4.24E-70 | 6.92E-68 |
| MEOX2 | -3.68953028 | 2.13E-57 | 1.39E-55 |
| AR | -3.660245364 | 6.56E-46 | 1.95E-44 |
| HLF | -3.658446204 | 1.46E-75 | 3.75E-73 |
| EGR3 | -3.545978634 | 5.47E-51 | 2.30E-49 |
| DBX2 | -3.542829619 | 6.56E-53 | 3.12E-51 |
| ALX4 | -3.512390559 | 3.55E-38 | 6.49E-37 |
| HIF3A | -3.50446655 | 1.48E-45 | 4.33E-44 |
| FOS | -3.386345185 | 2.81E-50 | 1.12E-48 |
| MEOX1 | -3.378003087 | 3.83E-52 | 1.74E-50 |
| PPARG | -3.301163783 | 3.14E-53 | 1.52E-51 |
| EBF1 | -3.281277655 | 8.73E-61 | 7.18E-59 |
| TFAP2B | -3.196343141 | 1.33E-13 | 4.82E-13 |
| EGR1 | -3.171111868 | 5.06E-57 | 3.24E-55 |
| LMX1A | -3.143993788 | 1.15E-28 | 1.11E-27 |
| TSHZ2 | -3.140941681 | 1.59E-73 | 3.59E-71 |
| NEUROG2 | -3.124166019 | 4.15E-20 | 2.32E-19 |
| EBF3 | -3.102455028 | 2.91E-56 | 1.77E-54 |
| HOXA6 | -3.100083283 | 8.82E-39 | 1.69E-37 |
| HOXA7 | -2.975735027 | 1.09E-44 | 2.99E-43 |
| KLF15 | -2.956453548 | 2.22E-47 | 7.24E-46 |
| HOXA5 | -2.944803731 | 2.28E-54 | 1.18E-52 |
| RHOXF1 | -2.942510475 | 1.58E-41 | 3.60E-40 |
| EBF2 | -2.895712438 | 2.30E-49 | 8.49E-48 |
| KLF4 | -2.838517374 | 1.03E-52 | 4.84E-51 |
| THRB | -2.807997121 | 9.96E-65 | 1.11E-62 |
| RUNX1T1 | -2.763327556 | 1.85E-59 | 1.39E-57 |
| DMRT2 | -2.750818476 | 1.01E-29 | 1.05E-28 |
| CSRNP3 | -2.6835642 | 1.15E-31 | 1.37E-30 |
| TCF21 | -2.666378935 | 1.80E-37 | 3.14E-36 |
| FOXI2 | -2.634310631 | 1.11E-33 | 1.53E-32 |
| NR3C2 | -2.624819334 | 3.80E-64 | 3.96E-62 |
| SOX5 | -2.609303616 | 4.10E-38 | 7.47E-37 |
| HOXA2 | -2.526459987 | 2.66E-37 | 4.58E-36 |
| SOX17 | -2.484137639 | 1.04E-46 | 3.26E-45 |
| FOXA1 | -2.469806913 | 4.44E-12 | 1.46E-11 |
| FEZF2 | -2.431785671 | 5.54E-21 | 3.27E-20 |
| ZSCAN23 | -2.413266822 | 9.16E-25 | 6.86E-24 |
| ZSCAN23.1 | -2.413266822 | 9.16E-25 | 6.86E-24 |
| ZNF385D | -2.41229805 | 1.70E-48 | 6.00E-47 |
| NR2F1 | -2.409824656 | 2.05E-38 | 3.80E-37 |
| TAL1 | -2.406124495 | 1.39E-50 | 5.68E-49 |
| ZSCAN4 | -2.403565311 | 2.19E-32 | 2.74E-31 |
| ZNF99 | -2.386886019 | 2.21E-24 | 1.63E-23 |
| FOXP2 | -2.384992719 | 1.80E-29 | 1.83E-28 |
| HOXA4 | -2.375217936 | 9.92E-38 | 1.76E-36 |
| PRDM16 | -2.35973611 | 5.88E-37 | 9.92E-36 |
| HOXA9 | -2.347112661 | 1.44E-29 | 1.47E-28 |
| ZNF385B | -2.344555399 | 2.51E-27 | 2.22E-26 |
| ZNF366 | -2.304254047 | 2.02E-36 | 3.31E-35 |
| ZNF423 | -2.299979761 | 7.36E-47 | 2.32E-45 |
| ZFP36 | -2.297335579 | 1.24E-39 | 2.51E-38 |
| TBX15 | -2.28232032 | 1.63E-35 | 2.51E-34 |
| RORB | -2.276556685 | 1.26E-21 | 7.77E-21 |
| ZNF208 | -2.256439789 | 3.45E-26 | 2.85E-25 |
| ZNF208.1 | -2.256439789 | 3.45E-26 | 2.85E-25 |
| MSX2 | -2.250484453 | 6.45E-21 | 3.80E-20 |
| ZNF214 | -2.245612532 | 1.12E-34 | 1.62E-33 |
| EGR2 | -2.23634987 | 7.71E-35 | 1.13E-33 |
| ZNF483 | -2.182913596 | 1.11E-42 | 2.71E-41 |
| ZNF727 | -2.169116168 | 6.76E-21 | 3.98E-20 |
| TWIST2 | -2.155648026 | 4.31E-29 | 4.28E-28 |
| NR5A2 | -2.148935223 | 1.81E-51 | 7.82E-50 |
| CPEB1 | -2.143993909 | 4.04E-39 | 7.92E-38 |
| NR0B1 | -2.132522212 | 1.14E-15 | 4.75E-15 |
| ERG | -2.122113928 | 5.66E-55 | 3.08E-53 |
| PKNOX2 | -2.081436601 | 3.56E-22 | 2.27E-21 |
| KLF2 | -2.077271058 | 1.19E-40 | 2.57E-39 |
| POU3F3 | -2.06958739 | 7.60E-09 | 1.97E-08 |
| EPAS1 | -2.064234807 | 9.51E-49 | 3.39E-47 |
| KLF9 | -2.063229469 | 4.19E-55 | 2.31E-53 |
| TCF23 | -2.062383521 | 3.34E-23 | 2.27E-22 |
| HOXD4 | -2.051658964 | 7.40E-27 | 6.37E-26 |
| CSRNP1 | -2.025203365 | 3.84E-43 | 9.57E-42 |
| CUX2 | -1.992256528 | 1.16E-11 | 3.69E-11 |
| FOXO1 | -1.975088423 | 3.23E-55 | 1.80E-53 |
| NR4A1 | -1.973762165 | 2.18E-24 | 1.60E-23 |
| NPAS4 | -1.962173628 | 2.88E-19 | 1.53E-18 |
| ZFHX4 | -1.9556835 | 7.88E-26 | 6.35E-25 |
| HOXD3 | -1.938426191 | 2.06E-36 | 3.37E-35 |
| MKX | -1.890272072 | 7.12E-17 | 3.23E-16 |
| SHOX | -1.882050777 | 7.49E-18 | 3.62E-17 |
| PAX3 | -1.88155651 | 4.73E-11 | 1.44E-10 |
| ZNF540 | -1.878478623 | 4.47E-55 | 2.46E-53 |
| PROX1 | -1.860973835 | 4.64E-20 | 2.58E-19 |
| IRX6 | -1.853557812 | 9.97E-14 | 3.65E-13 |
| HOXA3 | -1.852761603 | 1.16E-23 | 8.13E-23 |
| NR3C1 | -1.851853752 | 1.87E-56 | 1.15E-54 |
| HOXD8 | -1.83759947 | 2.04E-43 | 5.17E-42 |
| CEBPA | -1.837494649 | 1.22E-20 | 7.05E-20 |
| OSR1 | -1.825442744 | 5.02E-15 | 2.01E-14 |
| FOXN1 | -1.816458395 | 1.14E-10 | 3.38E-10 |
| BNC1 | -1.787844681 | 4.35E-09 | 1.15E-08 |
| ATF3 | -1.787207134 | 2.55E-27 | 2.26E-26 |
| PRDM5 | -1.782715023 | 6.09E-46 | 1.82E-44 |
| ZNF781 | -1.771002865 | 6.69E-41 | 1.47E-39 |
| ZMAT1 | -1.769066307 | 8.05E-36 | 1.26E-34 |
| ZNF835 | -1.766106356 | 3.01E-23 | 2.05E-22 |
| ZNF835.1 | -1.766106356 | 3.01E-23 | 2.05E-22 |
| ZFPM2 | -1.761008932 | 2.53E-23 | 1.73E-22 |
| SOX7 | -1.746820565 | 2.83E-35 | 4.27E-34 |
| ZNF154 | -1.730360986 | 5.27E-32 | 6.45E-31 |
| LHX6 | -1.729687391 | 1.84E-30 | 2.01E-29 |
| TEF | -1.72878922 | 1.20E-50 | 4.90E-49 |
| SETBP1 | -1.706861074 | 6.54E-40 | 1.35E-38 |
| ZNF677 | -1.70642296 | 2.38E-27 | 2.11E-26 |
| NR4A2 | -1.702952571 | 1.73E-26 | 1.45E-25 |
| SPDEF | -1.696968615 | 1.58E-06 | 3.37E-06 |
| GATA3 | -1.692527006 | 2.90E-14 | 1.10E-13 |
| GLI3 | -1.674167661 | 3.03E-33 | 4.04E-32 |
| ZEB1 | -1.654180042 | 1.25E-29 | 1.29E-28 |
| PROX2 | -1.650165859 | 3.42E-21 | 2.05E-20 |
| SP5 | -1.650076302 | 4.54E-12 | 1.49E-11 |
| GATA2 | -1.647408483 | 2.64E-19 | 1.40E-18 |
| ZBED9 | -1.643932593 | 4.00E-09 | 1.06E-08 |
| FOXE1 | -1.635019498 | 3.24E-10 | 9.30E-10 |
| LTF | -1.62206066 | 2.19E-05 | 4.21E-05 |
| PEG3 | -1.609552026 | 1.86E-07 | 4.31E-07 |
| ARID5B | -1.606132211 | 7.97E-52 | 3.54E-50 |
| ZNF334 | -1.601967046 | 1.11E-16 | 4.96E-16 |
| TBX18 | -1.594531132 | 1.14E-16 | 5.08E-16 |
| NFIA | -1.593358158 | 1.43E-26 | 1.21E-25 |
| TBX3 | -1.592427059 | 2.10E-27 | 1.87E-26 |
| ZFP2 | -1.581600468 | 1.16E-44 | 3.17E-43 |
| SALL2 | -1.579473919 | 5.94E-27 | 5.15E-26 |
| EMX2 | -1.577966557 | 1.43E-17 | 6.80E-17 |
| MEIS2 | -1.576662789 | 1.14E-28 | 1.10E-27 |
| MECOM | -1.567456752 | 4.99E-26 | 4.07E-25 |
| CREB5 | -1.537995874 | 2.96E-25 | 2.29E-24 |
| RAX2 | -1.53359939 | 5.52E-21 | 3.26E-20 |
| SIM1 | -1.532671858 | 6.17E-07 | 1.37E-06 |
| DACH2 | -1.532121521 | 8.47E-11 | 2.54E-10 |
| ESRRB | -1.510192484 | 1.42E-13 | 5.15E-13 |
| IRX1 | -1.5084026 | 2.52E-07 | 5.78E-07 |
| MITF | -1.503439233 | 4.63E-34 | 6.50E-33 |
| HOXA10 | -1.502001045 | 5.32E-14 | 1.98E-13 |
| POU6F2 | -1.492189364 | 4.30E-06 | 8.81E-06 |
| ZNF662 | -1.491437214 | 7.50E-27 | 6.45E-26 |
| ZBTB7C | -1.489386343 | 2.26E-17 | 1.06E-16 |
| ZNF175 | -1.487012872 | 1.44E-37 | 2.52E-36 |
| ZNF175.1 | -1.487012872 | 1.44E-37 | 2.52E-36 |
| BHLHE41 | -1.485696086 | 9.38E-29 | 9.05E-28 |
| ETV1 | -1.481763824 | 1.23E-22 | 8.08E-22 |
| ZBED6 | -1.470303736 | 2.70E-10 | 7.79E-10 |
| GLI1 | -1.468948583 | 2.65E-18 | 1.32E-17 |
| NHLH2 | -1.463083657 | 2.24E-09 | 6.03E-09 |
| ZNF106 | -1.455928823 | 1.18E-37 | 2.08E-36 |
| HOXC5 | -1.439208488 | 3.05E-20 | 1.72E-19 |
| ZNF536 | -1.43534669 | 6.23E-12 | 2.02E-11 |
| ZEB2 | -1.423789262 | 4.25E-24 | 3.06E-23 |
| THRA | -1.421574723 | 6.74E-53 | 3.20E-51 |
| JUN | -1.420717118 | 1.20E-30 | 1.32E-29 |
| LMX1B | -1.418556489 | 8.83E-10 | 2.46E-09 |
| HOXD9 | -1.409860626 | 5.33E-30 | 5.65E-29 |
| PRDM8 | -1.404291757 | 8.74E-26 | 7.01E-25 |
| ZBTB4 | -1.393115399 | 4.37E-81 | 1.97E-78 |
| NR4A3 | -1.383908139 | 5.39E-11 | 1.64E-10 |
| SMAD9 | -1.378154416 | 7.18E-33 | 9.32E-32 |
| FOXN3 | -1.369744793 | 4.86E-37 | 8.25E-36 |
| TWIST1 | -1.365421136 | 9.70E-16 | 4.07E-15 |
| DPRX | -1.361843958 | 2.14E-19 | 1.14E-18 |
| NPAS3 | -1.355775334 | 2.62E-23 | 1.79E-22 |
| PURG | -1.352593026 | 6.68E-14 | 2.48E-13 |
| ZNF132 | -1.343545648 | 4.87E-39 | 9.46E-38 |
| ESR2 | -1.342988026 | 1.16E-20 | 6.70E-20 |
| TFDP3 | -1.342414558 | 7.70E-07 | 1.69E-06 |
| ZNF441 | -1.338233767 | 9.37E-35 | 1.36E-33 |
| STAT5B | -1.326981493 | 5.40E-56 | 3.21E-54 |
| GSC | -1.326615993 | 5.11E-15 | 2.04E-14 |
| ZNF671 | -1.325590927 | 3.61E-29 | 3.60E-28 |
| TEAD1 | -1.318106449 | 2.30E-30 | 2.49E-29 |
| KLF14 | -1.317495924 | 2.22E-09 | 5.99E-09 |
| KLF8 | -1.317494639 | 5.53E-23 | 3.72E-22 |
| HOXB4 | -1.307171559 | 2.57E-17 | 1.20E-16 |
| HOXB8 | -1.306818998 | 6.97E-10 | 1.95E-09 |
| ZNF471 | -1.298331341 | 1.07E-31 | 1.29E-30 |
| HOXB3 | -1.297157602 | 1.19E-15 | 4.96E-15 |
| NR2E3 | -1.2829823 | 6.93E-18 | 3.36E-17 |
| HOXA13 | -1.280989355 | 2.56E-07 | 5.86E-07 |
| TCF4 | -1.273666862 | 2.13E-26 | 1.78E-25 |
| NFAT5 | -1.268512331 | 5.34E-19 | 2.78E-18 |
| ZNF844 | -1.264584129 | 2.73E-25 | 2.12E-24 |
| FOXI1 | -1.258074433 | 0.001228375 | 0.001988078 |
| ZHX3 | -1.256658908 | 6.04E-47 | 1.92E-45 |
| ZNF608 | -1.255337065 | 1.69E-24 | 1.25E-23 |
| MSX1 | -1.24409379 | 8.51E-16 | 3.58E-15 |
| BNC2 | -1.243184997 | 2.13E-15 | 8.74E-15 |
| ZNF442 | -1.240828144 | 1.96E-31 | 2.30E-30 |
| ZNF843 | -1.233196073 | 1.57E-33 | 2.13E-32 |
| CREBL2 | -1.232141784 | 1.95E-45 | 5.63E-44 |
| KLF10 | -1.226927648 | 8.86E-30 | 9.24E-29 |
| BCL6 | -1.225118579 | 1.10E-31 | 1.32E-30 |
| TBX22 | -1.221644613 | 1.81E-09 | 4.93E-09 |
| TSHZ3 | -1.217063707 | 2.99E-25 | 2.32E-24 |
| ZNF534 | -1.20921717 | 1.16E-10 | 3.44E-10 |
| HOXB1 | -1.204915249 | 1.22E-10 | 3.60E-10 |
| ZNF763 | -1.202640412 | 2.81E-26 | 2.33E-25 |
| ZNF763.1 | -1.202640412 | 2.81E-26 | 2.33E-25 |
| ZNF582 | -1.199655491 | 4.93E-26 | 4.03E-25 |
| ZBTB20 | -1.199228248 | 1.88E-15 | 7.71E-15 |
| ZBTB20.1 | -1.199228248 | 1.88E-15 | 7.71E-15 |
| STAT5A | -1.195451139 | 1.11E-28 | 1.06E-27 |
| MEF2C | -1.193587785 | 3.32E-25 | 2.56E-24 |
| HOXD10 | -1.191810076 | 2.19E-12 | 7.33E-12 |
| RFX2 | -1.189169123 | 2.99E-29 | 3.00E-28 |
| ZNF660 | -1.186668514 | 9.23E-23 | 6.11E-22 |
| OSR2 | -1.176570683 | 4.88E-11 | 1.49E-10 |
| HEY2 | -1.169092041 | 1.84E-09 | 5.01E-09 |
| TSHZ1 | -1.162930354 | 5.66E-42 | 1.32E-40 |
| ZNF578 | -1.157391638 | 1.78E-11 | 5.61E-11 |
| ZNF578.1 | -1.157391638 | 1.78E-11 | 5.61E-11 |
| ZNF502 | -1.156284062 | 2.86E-19 | 1.51E-18 |
| FOXO4 | -1.15272809 | 5.08E-37 | 8.62E-36 |
| MYF6 | -1.149674323 | 3.52E-07 | 7.96E-07 |
| ZNF219 | -1.147137641 | 1.83E-19 | 9.80E-19 |
| FOXP1 | -1.140264377 | 6.85E-41 | 1.50E-39 |
| PURA | -1.138062838 | 1.40E-45 | 4.08E-44 |
| ZNF699 | -1.137758208 | 2.26E-16 | 9.87E-16 |
| MEIS1 | -1.129434199 | 2.25E-23 | 1.55E-22 |
| HAND2 | -1.129145189 | 5.49E-11 | 1.66E-10 |
| IRX4 | -1.128022753 | 0.002817293 | 0.00437334 |
| L3MBTL1 | -1.121980681 | 1.43E-17 | 6.81E-17 |
| PRDM6 | -1.120662712 | 3.17E-14 | 1.20E-13 |
| TBX5 | -1.120046005 | 2.14E-11 | 6.71E-11 |
| FLI1 | -1.115302898 | 1.09E-21 | 6.74E-21 |
| ATF7-NPFF | -1.114146537 | 1.29E-23 | 9.02E-23 |
| MAF | -1.113405397 | 7.43E-22 | 4.64E-21 |
| BACH2 | -1.111978717 | 4.18E-17 | 1.92E-16 |
| CARF | -1.105628156 | 7.28E-38 | 1.31E-36 |
| ZNF25 | -1.104191173 | 9.51E-41 | 2.07E-39 |
| ZNF729 | -1.097337204 | 6.95E-05 | 0.000127223 |
| ZSCAN1 | -1.097135993 | 3.27E-05 | 6.18E-05 |
| HOXB2 | -1.093787709 | 5.69E-14 | 2.12E-13 |
| ESRRG | -1.086378642 | 3.89E-07 | 8.75E-07 |
| ZNF704 | -1.084047101 | 5.86E-15 | 2.33E-14 |
| POU6F1 | -1.083130055 | 1.92E-38 | 3.58E-37 |
| ZNF516 | -1.081374197 | 3.68E-18 | 1.81E-17 |
| GTF2IRD2 | -1.075112748 | 4.96E-40 | 1.03E-38 |
| MBNL2 | -1.071341581 | 2.21E-29 | 2.23E-28 |
| BAZ2B | -1.066037628 | 2.40E-29 | 2.42E-28 |
| HIC1 | -1.05862635 | 1.05E-17 | 5.04E-17 |
| ZNF486 | -1.055725439 | 1.47E-10 | 4.32E-10 |
| NR1D1 | -1.054651203 | 7.44E-19 | 3.83E-18 |
| GLIS1 | -1.053354781 | 1.19E-09 | 3.28E-09 |
| KLF6 | -1.049951843 | 9.42E-23 | 6.23E-22 |
| ZNF804B | -1.046865974 | 2.78E-07 | 6.34E-07 |
| IRX2 | -1.046644835 | 2.22E-07 | 5.10E-07 |
| ZNF395 | -1.040673995 | 1.38E-40 | 2.97E-39 |
| TSC22D1 | -1.040417438 | 8.10E-33 | 1.05E-31 |
| ZBTB47 | -1.03505837 | 2.00E-25 | 1.57E-24 |
| ZNF528 | -1.034545319 | 5.50E-19 | 2.86E-18 |
| ZNF311 | -1.034065582 | 5.04E-11 | 1.53E-10 |
| NEUROD2 | -1.033659475 | 1.03E-08 | 2.64E-08 |
| RORC | -1.029003259 | 2.82E-06 | 5.88E-06 |
| ZNF559-ZNF177 | -1.019064042 | 1.26E-12 | 4.30E-12 |
| GTF2IRD2B | -1.015310143 | 1.67E-41 | 3.79E-40 |
| CCDC169-SOHLH2 | -1.009890455 | 1.46E-07 | 3.40E-07 |
| MXI1 | -1.008312005 | 7.03E-33 | 9.14E-32 |
| TCF15 | -1.0054966 | 7.22E-06 | 1.45E-05 |
| ZNF98 | -1.003435851 | 9.61E-05 | 0.000173634 |

| **Supplementary Table 6**. JASPAR prediction result of interaction between plus-strand LYPLAL1-DT promoter and 38 candidate TFs | | | | | | |
| --- | --- | --- | --- | --- | --- | --- |
| Matrix ID | Name | JASPAR Score | Start(LYPLAL1-DT promoter) | End(LYPLAL1-DT promoter) | Strand | Predicted sequence |
| MA0480.1 | Foxo1 | 13.006845 | 938 | 948 | + | TTTTGTTTTTA |
| MA0848.1 | FOXO4 | 12.713575 | 1339 | 1345 | + | GTAAACA |
| MA0606.2 | Nfat5 | 11.300503 | 166 | 177 | + | CAAAGGAAAAAA |
| UN0332.1 | ZNF534 | 11.065975 | 1200 | 1212 | + | GGTCCTGCCCCTG |
| MA0606.2 | Nfat5 | 10.744961 | 1392 | 1403 | + | GCCTGGAAAAAG |
| MA0090.1 | TEAD1 | 10.709221 | 1088 | 1099 | + | CACAATCCAGTG |
| UN0332.1 | ZNF534 | 10.454845 | 471 | 483 | + | CGTCTTTCCCTTT |
| MA1489.1 | FOXN3 | 9.561016 | 1339 | 1346 | + | GTAAACAG |
| MA0843.1 | TEF | 9.397036 | 1131 | 1142 | + | TGTTAAGTAACG |
| MA0480.2 | Foxo1 | 8.544625 | 480 | 490 | + | CTTTAAACACT |
| MA0090.2 | TEAD1 | 7.819254 | 207 | 216 | + | GACATACCCT |
| MA0090.2 | TEAD1 | 7.4785066 | 683 | 692 | + | AGAATACCTC |
| MA0090.2 | TEAD1 | 7.3789454 | 121 | 130 | + | CGCATACTTG |
| MA0090.2 | TEAD1 | 7.332672 | 146 | 155 | + | TAAATTCTTG |
| MA0090.2 | TEAD1 | 7.310979 | 133 | 142 | + | AGAATTCTTT |
| MA0090.2 | TEAD1 | 7.188091 | 453 | 462 | + | CACCTTCTAA |
| MA0848.1 | FOXO4 | 7.148708 | 996 | 1002 | + | AAAAACA |
| MA0848.1 | FOXO4 | 7.148708 | 1447 | 1453 | + | AAAAACA |
| MA1108.2 | MXI1 | 6.923012 | 444 | 453 | + | TACACACGTC |
| MA1108.2 | MXI1 | 6.808144 | 1154 | 1163 | + | AGCACACGTT |
| MA0090.2 | TEAD1 | 6.5589623 | 110 | 119 | + | CACATTATAC |
| MA0090.2 | TEAD1 | 6.3922715 | 787 | 796 | + | CACTTTCTTC |
| MA0090.2 | TEAD1 | 6.2233257 | 732 | 741 | + | CCAATTCCCT |
| MA1108.2 | MXI1 | 5.4405785 | 184 | 193 | + | CATACATGAC |
| MA1108.2 | MXI1 | 5.4128885 | 845 | 854 | + | ACCACATTTT |
| MA1108.2 | MXI1 | 4.790597 | 1996 | 2005 | + | CGCGCATGCG |

| **Supplementary Table 7**. Genes significantly correlated with LYPLAL1-DT | | |
| --- | --- | --- |
| Gene | Spearman *ρ* | *p* value |
| RCOR3 | 0.541 | 1.51E-13 |
| KLHL24 | 0.521 | 1.60E-12 |
| KMT5B | 0.52 | 1.83E-12 |
| GOPC | 0.518 | 2.18E-12 |
| RNF13 | 0.513 | 3.93E-12 |
| ANGEL2 | 0.51 | 5.81E-12 |
| ETV3 | 0.509 | 6.62E-12 |
| GPATCH8 | 0.504 | 1.08E-11 |
| BICRAL | 0.5 | 1.72E-11 |
| WDR26 | 0.498 | 1.99E-11 |
| RAB3GAP2 | 0.494 | 3.07E-11 |
| ARID4B | 0.494 | 3.33E-11 |
| ARMC8 | 0.492 | 3.84E-11 |
| ZNF641 | 0.491 | 4.26E-11 |
| RAB29 | 0.491 | 4.30E-11 |
| DCAF16 | 0.488 | 5.92E-11 |
| MBOAT1 | 0.487 | 6.55E-11 |
| SPAST | 0.485 | 8.27E-11 |
| OSBPL1A | 0.484 | 9.25E-11 |
| PTPN14 | 0.482 | 1.05E-10 |
| C3orf38 | 0.481 | 1.20E-10 |
| MARF1 | 0.481 | 1.16E-10 |
| CGGBP1 | 0.481 | 1.24E-10 |
| MFN1 | 0.481 | 1.19E-10 |
| BROX | 0.48 | 1.39E-10 |
| CCDC50 | 0.479 | 1.45E-10 |
| ANKRD31 | 0.479 | 1.53E-10 |
| ZNF561 | 0.479 | 1.43E-10 |
| ESCO1 | 0.478 | 1.65E-10 |
| ZBTB11 | 0.477 | 1.81E-10 |
| MAP4K3 | 0.477 | 1.82E-10 |
| BBS10 | 0.476 | 2.03E-10 |
| DCAF17 | 0.476 | 1.97E-10 |
| ZNF33A | 0.476 | 2.01E-10 |
| ATF7IP | 0.476 | 1.96E-10 |
| SOCS5 | 0.474 | 2.38E-10 |
| SDE2 | 0.474 | 2.52E-10 |
| PCNP | 0.473 | 2.63E-10 |
| TASOR | 0.472 | 3.10E-10 |
| PFKFB2 | 0.471 | 3.16E-10 |
| THAP2 | 0.47 | 3.71E-10 |
| DSTYK | 0.468 | 4.30E-10 |
| EPC2 | 0.468 | 4.31E-10 |
| RPS6KC1 | 0.468 | 4.38E-10 |
| TMEM182 | 0.468 | 4.21E-10 |
| LANCL1 | 0.467 | 4.92E-10 |
| SLC25A36 | 0.466 | 5.07E-10 |
| RASA2 | 0.466 | 5.10E-10 |
| ZFP14 | 0.465 | 6.09E-10 |
| RSBN1 | 0.465 | 5.89E-10 |
| MSL2 | 0.465 | 6.12E-10 |
| NR1D2 | 0.465 | 5.77E-10 |
| GGA2 | 0.464 | 6.69E-10 |
| NEK7 | 0.464 | 6.45E-10 |
| APPL1 | 0.464 | 6.27E-10 |
| IFT80 | 0.464 | 6.72E-10 |
| RBBP6 | 0.464 | 6.73E-10 |
| EPC1 | 0.464 | 6.64E-10 |
| OXR1 | 0.464 | 6.67E-10 |
| GGNBP2 | 0.463 | 7.08E-10 |
| SS18 | 0.463 | 7.15E-10 |
| DNAJC27 | 0.462 | 7.51E-10 |
| ZNF14 | 0.462 | 7.60E-10 |
| KANSL1 | 0.462 | 7.63E-10 |
| MLH3 | 0.461 | 8.31E-10 |
| PRMT9 | 0.461 | 8.45E-10 |
| DCUN1D1 | 0.461 | 8.42E-10 |
| PDPR | 0.461 | 8.52E-10 |
| ORC4 | 0.46 | 9.13E-10 |
| ZC3H11A | 0.459 | 1.00E-09 |
| MBNL2 | 0.459 | 1.03E-09 |
| MTR | 0.459 | 1.01E-09 |
| YIPF4 | 0.459 | 1.06E-09 |
| TOR1AIP1 | 0.459 | 1.02E-09 |
| BBS2 | 0.459 | 9.99E-10 |
| CEP44 | 0.458 | 1.10E-09 |
| FZD6 | 0.458 | 1.13E-09 |
| NAPEPLD | 0.458 | 1.16E-09 |
| HNRNPLL | 0.458 | 1.17E-09 |
| ZNF669 | 0.457 | 1.24E-09 |
| ZNF25 | 0.457 | 1.20E-09 |
| SENP6 | 0.457 | 1.20E-09 |
| ZNF654 | 0.456 | 1.36E-09 |
| PUM2 | 0.455 | 1.46E-09 |
| IRF6 | 0.455 | 1.50E-09 |
| BBX | 0.455 | 1.56E-09 |
| SOS1 | 0.454 | 1.58E-09 |
| MMAA | 0.454 | 1.69E-09 |
| RANBP6 | 0.454 | 1.65E-09 |
| PPM1B | 0.454 | 1.61E-09 |
| ZNF507 | 0.453 | 1.87E-09 |
| CNST | 0.453 | 1.82E-09 |
| CTCF | 0.452 | 1.95E-09 |
| DBR1 | 0.452 | 2.04E-09 |
| ZNF566 | 0.452 | 2.03E-09 |
| TLR5 | 0.452 | 2.00E-09 |
| KDM2A | 0.452 | 1.94E-09 |
| ZNF260 | 0.452 | 1.91E-09 |
| CYP39A1 | 0.45 | 2.46E-09 |
| MYNN | 0.449 | 2.56E-09 |
| FAM200A | 0.449 | 2.69E-09 |
| ZNF461 | 0.448 | 2.80E-09 |
| DLG1 | 0.448 | 2.79E-09 |
| ETNK1 | 0.448 | 2.78E-09 |
| CHIC1 | 0.448 | 2.83E-09 |
| IARS2 | 0.447 | 3.11E-09 |
| CDKN1B | 0.447 | 3.06E-09 |
| SEC14L5 | 0.447 | 3.16E-09 |
| YTHDC1 | 0.447 | 3.10E-09 |
| ZNF148 | 0.446 | 3.50E-09 |
| ATF6 | 0.446 | 3.33E-09 |
| SEPTIN10 | 0.446 | 3.37E-09 |
| MAN1A2 | 0.446 | 3.52E-09 |
| ZSCAN26 | 0.446 | 3.35E-09 |
| TMEM184C | 0.446 | 3.49E-09 |
| FAM13A | 0.446 | 3.39E-09 |
| NEPRO | 0.445 | 3.57E-09 |
| UTP25 | 0.445 | 3.63E-09 |
| GOLPH3L | 0.445 | 3.82E-09 |
| GMCL1 | 0.445 | 3.73E-09 |
| DZIP3 | 0.445 | 3.80E-09 |
| KLF3 | 0.445 | 3.73E-09 |
| PIK3CB | 0.445 | 3.62E-09 |
| MIB1 | 0.444 | 3.91E-09 |
| CEPT1 | 0.444 | 3.95E-09 |
| RBBP5 | 0.444 | 4.16E-09 |
| RBM48 | 0.444 | 4.07E-09 |
| PHF3 | 0.444 | 3.96E-09 |
| ZBTB33 | 0.443 | 4.52E-09 |
| FBXO28 | 0.443 | 4.45E-09 |
| PIK3CA | 0.442 | 5.02E-09 |
| ARHGEF9 | 0.441 | 5.33E-09 |
| SENP1 | 0.441 | 5.24E-09 |
| TP53BP2 | 0.441 | 5.46E-09 |
| ADNP | 0.441 | 5.36E-09 |
| RO60 | 0.441 | 5.37E-09 |
| SLC30A6 | 0.441 | 5.11E-09 |
| VANGL2 | 0.44 | 5.94E-09 |
| ZMYM6 | 0.44 | 6.00E-09 |
| BPNT1 | 0.44 | 5.82E-09 |
| ZFAT | 0.44 | 5.95E-09 |
| RFX3 | 0.44 | 5.94E-09 |
| LMBRD1 | 0.439 | 6.62E-09 |
| HBP1 | 0.439 | 6.29E-09 |
| PTAR1 | 0.439 | 6.54E-09 |
| PTPN4 | 0.439 | 6.21E-09 |
| CLDND1 | 0.438 | 6.83E-09 |
| ZBTB24 | 0.438 | 6.89E-09 |
| GON4L | 0.438 | 7.00E-09 |
| ANKRD12 | 0.438 | 6.96E-09 |
| NCK1 | 0.438 | 6.95E-09 |
| MDH1B | 0.437 | 7.41E-09 |
| PI4K2B | 0.437 | 7.27E-09 |
| FBXO11 | 0.437 | 7.47E-09 |
| ZNF639 | 0.436 | 8.17E-09 |
| RIOK3 | 0.436 | 8.43E-09 |
| ZFP1 | 0.436 | 8.48E-09 |
| DUSP19 | 0.435 | 8.88E-09 |
| SLC35A5 | 0.435 | 9.01E-09 |
| ANAPC4 | 0.435 | 9.39E-09 |
| NSUN3 | 0.435 | 9.06E-09 |
| TANK | 0.435 | 8.71E-09 |
| TBC1D23 | 0.435 | 9.27E-09 |
| ELF2 | 0.435 | 9.38E-09 |
| THAP5 | 0.435 | 8.93E-09 |
| RBL2 | 0.434 | 9.69E-09 |
| BRD2 | 0.434 | 1.02E-08 |
| PLEKHG4 | 0.434 | 9.61E-09 |
| ABCB10 | 0.434 | 9.66E-09 |
| MRAS | 0.433 | 1.05E-08 |
| ZNF146 | 0.433 | 1.07E-08 |
| ABHD13 | 0.433 | 1.10E-08 |
| RPRD2 | 0.433 | 1.11E-08 |
| BNIP2 | 0.433 | 1.11E-08 |
| TRMT1L | 0.433 | 1.07E-08 |
| CIPC | 0.432 | 1.21E-08 |
| CREBBP | 0.431 | 1.33E-08 |
| ZNF322 | 0.431 | 1.31E-08 |
| MARCHF7 | 0.431 | 1.24E-08 |
| YY1AP1 | 0.431 | 1.25E-08 |
| PAK2 | 0.431 | 1.32E-08 |
| ZNF292 | 0.431 | 1.28E-08 |
| PRPF40A | 0.431 | 1.27E-08 |
| PRKCI | 0.431 | 1.31E-08 |
| NIPAL3 | 0.431 | 1.24E-08 |
| CRB1 | 0.431 | 1.29E-08 |
| RNF146 | 0.431 | 1.32E-08 |
| ATP13A3 | 0.431 | 1.29E-08 |
| C6orf89 | 0.431 | 1.29E-08 |
| TSNAX | 0.431 | 1.25E-08 |
| ZNF567 | 0.43 | 1.42E-08 |
| NBR1 | 0.43 | 1.42E-08 |
| EXOC8 | 0.43 | 1.36E-08 |
| SUCO | 0.43 | 1.33E-08 |
| TIGD7 | 0.43 | 1.41E-08 |
| UBXN7 | 0.429 | 1.49E-08 |
| SCYL3 | 0.429 | 1.45E-08 |
| MBD5 | 0.429 | 1.47E-08 |
| ZBTB41 | 0.429 | 1.45E-08 |
| MBTD1 | 0.429 | 1.49E-08 |
| DSG2 | 0.429 | 1.54E-08 |
| PALB2 | 0.429 | 1.47E-08 |
| PPP1R2 | 0.428 | 1.71E-08 |
| WDR5B | 0.428 | 1.67E-08 |
| THAP9 | 0.428 | 1.65E-08 |
| BIRC2 | 0.428 | 1.61E-08 |
| LRP6 | 0.428 | 1.58E-08 |
| KLF11 | 0.428 | 1.61E-08 |
| ZNF664 | 0.428 | 1.68E-08 |
| DYNC1LI2 | 0.428 | 1.61E-08 |
| RAB28 | 0.428 | 1.59E-08 |
| DHFR2 | 0.428 | 1.58E-08 |
| PPFIBP2 | 0.428 | 1.72E-08 |
| HAUS3 | 0.427 | 1.76E-08 |
| ZKSCAN8 | 0.427 | 1.79E-08 |
| VPS52 | 0.427 | 1.83E-08 |
| CREB1 | 0.427 | 1.84E-08 |
| MOB1B | 0.427 | 1.84E-08 |
| FAM20B | 0.426 | 1.94E-08 |
| KLF5 | 0.426 | 2.04E-08 |
| NCOA2 | 0.426 | 2.02E-08 |
| ACAP2 | 0.426 | 1.88E-08 |
| OTUD4 | 0.426 | 1.89E-08 |
| THNSL1 | 0.426 | 1.98E-08 |
| ASB8 | 0.426 | 1.88E-08 |
| REST | 0.426 | 2.00E-08 |
| GAB1 | 0.426 | 1.99E-08 |
| CLUL1 | 0.425 | 2.20E-08 |
| MMGT1 | 0.425 | 2.19E-08 |
| RC3H1 | 0.425 | 2.20E-08 |
| MAML2 | 0.424 | 2.26E-08 |
| ZDHHC23 | 0.424 | 2.23E-08 |
| ZNF670 | 0.424 | 2.30E-08 |
| WAC | 0.424 | 2.39E-08 |
| USP13 | 0.424 | 2.37E-08 |
| TTC13 | 0.424 | 2.40E-08 |
| HLTF | 0.423 | 2.51E-08 |
| SWT1 | 0.423 | 2.60E-08 |
| ZBTB14 | 0.423 | 2.44E-08 |
| SLC26A2 | 0.423 | 2.56E-08 |
| CHD4 | 0.423 | 2.47E-08 |
| ZKSCAN4 | 0.422 | 2.76E-08 |
| ROCK1 | 0.422 | 2.66E-08 |
| DENND6A | 0.422 | 2.73E-08 |
| RAD17 | 0.422 | 2.82E-08 |
| SIKE1 | 0.422 | 2.66E-08 |
| ARHGEF11 | 0.422 | 2.82E-08 |
| TRAPPC8 | 0.422 | 2.70E-08 |
| ZNF627 | 0.422 | 2.85E-08 |
| MFSD4A | 0.422 | 2.75E-08 |
| ABRAXAS1 | 0.422 | 2.80E-08 |
| STRN | 0.422 | 2.75E-08 |
| FBXL4 | 0.422 | 2.69E-08 |
| PPP4R3B | 0.421 | 3.04E-08 |
| HNRNPH3 | 0.421 | 3.03E-08 |
| ZNF675 | 0.421 | 3.05E-08 |
| IMPACT | 0.421 | 2.99E-08 |
| CTNND1 | 0.421 | 3.02E-08 |
| FAM168B | 0.421 | 3.00E-08 |
| ZNF721 | 0.421 | 3.09E-08 |
| EPM2A | 0.421 | 2.98E-08 |
| ZNF383 | 0.421 | 2.95E-08 |
| KDM3A | 0.421 | 3.09E-08 |
| ZNF708 | 0.421 | 2.89E-08 |
| DHX15 | 0.421 | 2.97E-08 |
| CASC3 | 0.42 | 3.21E-08 |
| SENP2 | 0.42 | 3.27E-08 |
| ADGRA3 | 0.42 | 3.36E-08 |
| YOD1 | 0.42 | 3.15E-08 |
| CCDC121 | 0.419 | 3.45E-08 |
| HSPA1L | 0.419 | 3.56E-08 |
| COMMD2 | 0.419 | 3.42E-08 |
| TBC1D5 | 0.419 | 3.55E-08 |
| USP38 | 0.419 | 3.54E-08 |
| ZNF791 | 0.419 | 3.55E-08 |
| FOXO4 | 0.419 | 3.58E-08 |
| FAM8A1 | 0.419 | 3.49E-08 |
| CILK1 | 0.419 | 3.55E-08 |
| MOSMO | 0.418 | 3.92E-08 |
| CTTNBP2NL | 0.418 | 3.83E-08 |
| NUDT21 | 0.418 | 3.82E-08 |
| PPFIA1 | 0.418 | 3.92E-08 |
| PPP1R21 | 0.418 | 3.94E-08 |
| LYSMD3 | 0.418 | 3.71E-08 |
| SLC24A1 | 0.417 | 4.22E-08 |
| SMG8 | 0.417 | 4.29E-08 |
| SP3 | 0.417 | 4.15E-08 |
| APPBP2 | 0.417 | 4.25E-08 |
| NPAT | 0.417 | 4.03E-08 |
| GNPTAB | 0.417 | 4.06E-08 |
| ZMYND11 | 0.417 | 4.00E-08 |
| ZNF396 | 0.417 | 4.28E-08 |
| TOMM20 | 0.416 | 4.39E-08 |
| DDR1 | 0.416 | 4.59E-08 |
| KLHL12 | 0.416 | 4.32E-08 |
| ZNF37A | 0.416 | 4.50E-08 |
| ZNF827 | 0.416 | 4.44E-08 |
| ZNF181 | 0.416 | 4.33E-08 |
| TBL1XR1 | 0.416 | 4.35E-08 |
| BLOC1S5 | 0.416 | 4.36E-08 |
| ZDHHC17 | 0.416 | 4.59E-08 |
| FYTTD1 | 0.416 | 4.62E-08 |
| MTMR6 | 0.416 | 4.50E-08 |
| CHMP2B | 0.416 | 4.52E-08 |
| ATXN1L | 0.416 | 4.66E-08 |
| TSPOAP1 | 0.415 | 4.81E-08 |
| ZNF623 | 0.415 | 4.95E-08 |
| UTP23 | 0.415 | 4.95E-08 |
| RNF168 | 0.415 | 4.99E-08 |
| MFSD14A | 0.415 | 5.00E-08 |
| USF3 | 0.415 | 4.87E-08 |
| OSBP | 0.415 | 4.93E-08 |
| TAF5L | 0.415 | 5.01E-08 |
| FOXN2 | 0.415 | 4.82E-08 |
| NBN | 0.415 | 5.05E-08 |
| PREPL | 0.415 | 4.85E-08 |
| FAM76A | 0.415 | 4.73E-08 |
| TRAM1L1 | 0.415 | 4.75E-08 |
| VEZF1 | 0.414 | 5.28E-08 |
| TBC1D32 | 0.414 | 5.06E-08 |
| BPTF | 0.414 | 5.09E-08 |
| CREBRF | 0.414 | 5.34E-08 |
| RNF227 | 0.414 | 5.21E-08 |
| MIA3 | 0.414 | 5.23E-08 |
| JMY | 0.414 | 5.35E-08 |
| BOD1L1 | 0.414 | 5.41E-08 |
| ACBD3 | 0.414 | 5.14E-08 |
| C1orf74 | 0.414 | 5.33E-08 |
| UHMK1 | 0.414 | 5.13E-08 |
| PTPRK | 0.414 | 5.06E-08 |
| PABPC4L | 0.414 | 5.44E-08 |
| VPS54 | 0.414 | 5.17E-08 |
| IMPA1 | 0.413 | 5.65E-08 |
| TAF1A | 0.413 | 5.62E-08 |
| ARHGAP21 | 0.413 | 5.79E-08 |
| RNF225 | 0.413 | 5.54E-08 |
| ZKSCAN3 | 0.413 | 5.83E-08 |
| MMUT | 0.413 | 5.55E-08 |
| USO1 | 0.413 | 5.68E-08 |
| TUG1 | 0.413 | 5.87E-08 |
| QTRT2 | 0.413 | 5.61E-08 |
| DSC2 | 0.412 | 6.31E-08 |
| ZFP62 | 0.412 | 6.04E-08 |
| VPS41 | 0.412 | 5.98E-08 |
| MSL1 | 0.412 | 6.12E-08 |
| ICE2 | 0.412 | 6.05E-08 |
| CDC40 | 0.412 | 6.23E-08 |
| TGDS | 0.412 | 6.03E-08 |
| RBM45 | 0.412 | 6.11E-08 |
| WASHC5 | 0.412 | 6.34E-08 |
| TRIM23 | 0.412 | 6.21E-08 |
| AIDA | 0.412 | 6.15E-08 |
| WWP2 | 0.411 | 6.94E-08 |
| SACM1L | 0.411 | 6.84E-08 |
| MAP10 | 0.411 | 6.73E-08 |
| MBTPS1 | 0.411 | 6.47E-08 |
| PCMTD2 | 0.411 | 6.98E-08 |
| ZFP91 | 0.411 | 6.77E-08 |
| N4BP1 | 0.411 | 6.62E-08 |
| UBR5 | 0.411 | 6.79E-08 |
| DDX17 | 0.41 | 7.01E-08 |
| ZNF182 | 0.41 | 7.48E-08 |
| SPRTN | 0.41 | 7.57E-08 |
| CCDC191 | 0.41 | 7.21E-08 |
| C2orf49 | 0.41 | 7.22E-08 |
| ZNF420 | 0.41 | 7.48E-08 |
| HEATR5B | 0.41 | 7.00E-08 |
| SIAH2 | 0.409 | 7.91E-08 |
| KRIT1 | 0.409 | 7.90E-08 |
| DVL3 | 0.409 | 8.18E-08 |
| ARL6 | 0.409 | 7.67E-08 |
| DHX9 | 0.409 | 7.93E-08 |
| EIF4A2 | 0.409 | 7.60E-08 |
| TRIM37 | 0.409 | 8.09E-08 |
| STK38 | 0.409 | 8.02E-08 |
| PPM1D | 0.409 | 8.19E-08 |
| CRIM1 | 0.409 | 7.66E-08 |
| CUL5 | 0.409 | 7.60E-08 |
| CPSF6 | 0.408 | 8.51E-08 |
| KIAA2026 | 0.408 | 8.59E-08 |
| SOCS7 | 0.408 | 8.88E-08 |
| ZBTB44 | 0.408 | 8.64E-08 |
| TNRC6A | 0.408 | 8.81E-08 |
| ADSS2 | 0.408 | 8.21E-08 |
| FAM135A | 0.408 | 8.25E-08 |
| ZNF607 | 0.408 | 8.81E-08 |
| B3GALNT1 | 0.408 | 8.80E-08 |
| CCSER2 | 0.408 | 8.49E-08 |
| SERTAD2 | 0.407 | 8.98E-08 |
| TCP11L2 | 0.407 | 9.07E-08 |
| ITSN2 | 0.407 | 9.01E-08 |
| GTF2IRD2B | 0.407 | 9.00E-08 |
| NFYA | 0.407 | 9.05E-08 |
| ARL6IP6 | 0.407 | 9.09E-08 |
| ZNF354C | 0.407 | 9.09E-08 |
| CEP192 | 0.407 | 9.24E-08 |
| ZNF529 | 0.407 | 9.21E-08 |
| RSF1 | 0.407 | 8.90E-08 |
| ASH1L | 0.407 | 9.40E-08 |
| METTL14 | 0.407 | 9.26E-08 |
| POGLUT1 | 0.407 | 9.58E-08 |
| ZFP90 | 0.406 | 1.01E-07 |
| OSGIN2 | 0.406 | 1.03E-07 |
| KRCC1 | 0.406 | 9.99E-08 |
| DIS3 | 0.406 | 1.01E-07 |
| USP4 | 0.406 | 1.01E-07 |
| MORC3 | 0.406 | 1.01E-07 |
| DCP1A | 0.406 | 1.01E-07 |
| CUL2 | 0.406 | 1.01E-07 |
| CENPC | 0.406 | 1.03E-07 |
| ZNF644 | 0.405 | 1.13E-07 |
| SMARCA2 | 0.405 | 1.07E-07 |
| CFAP97 | 0.405 | 1.11E-07 |
| RIF1 | 0.405 | 1.06E-07 |
| DHX36 | 0.405 | 1.08E-07 |
| PCMTD1 | 0.405 | 1.10E-07 |
| TRPS1 | 0.405 | 1.09E-07 |
| RAD9B | 0.405 | 1.05E-07 |
| ZNF333 | 0.405 | 1.07E-07 |
| ZHX1 | 0.405 | 1.10E-07 |
| ZFP30 | 0.405 | 1.09E-07 |
| PCGF5 | 0.405 | 1.11E-07 |
| PPP6R3 | 0.405 | 1.07E-07 |
| CCNG2 | 0.404 | 1.21E-07 |
| SF3B1 | 0.404 | 1.19E-07 |
| TMEM128 | 0.404 | 1.18E-07 |
| IRF2BP2 | 0.404 | 1.18E-07 |
| ZNF594 | 0.404 | 1.22E-07 |
| FXR1 | 0.404 | 1.14E-07 |
| TTC5 | 0.404 | 1.16E-07 |
| CD2AP | 0.404 | 1.17E-07 |
| DNAJC14 | 0.403 | 1.26E-07 |
| ZNF790 | 0.403 | 1.27E-07 |
| NAB1 | 0.403 | 1.23E-07 |
| NUP133 | 0.403 | 1.28E-07 |
| USP49 | 0.403 | 1.31E-07 |
| ZNF24 | 0.403 | 1.27E-07 |
| DBT | 0.403 | 1.22E-07 |
| TMEM30A | 0.403 | 1.30E-07 |
| ZFP82 | 0.403 | 1.27E-07 |
| ZSCAN25 | 0.403 | 1.24E-07 |
| DDX59 | 0.403 | 1.22E-07 |
| ZNF718 | 0.402 | 1.41E-07 |
| ATF6B | 0.402 | 1.34E-07 |
| ZNF184 | 0.402 | 1.33E-07 |
| ADAR | 0.402 | 1.40E-07 |
| CDC73 | 0.402 | 1.36E-07 |
| ATMIN | 0.402 | 1.39E-07 |
| CLCN3 | 0.402 | 1.41E-07 |
| ZSCAN30 | 0.402 | 1.33E-07 |
| GOLGA4 | 0.402 | 1.39E-07 |
| KANK1 | 0.402 | 1.34E-07 |
| UFL1 | 0.402 | 1.37E-07 |
| ABHD18 | 0.402 | 1.36E-07 |
| ZFP3 | 0.402 | 1.41E-07 |
| ZNF845 | 0.402 | 1.38E-07 |
| PUM1 | 0.401 | 1.47E-07 |
| DCAF1 | 0.401 | 1.54E-07 |
| FAM160A1 | 0.401 | 1.51E-07 |
| WDR48 | 0.401 | 1.51E-07 |
| RYK | 0.401 | 1.44E-07 |
| RAD21 | 0.401 | 1.46E-07 |
| ZNF638 | 0.401 | 1.43E-07 |

| **Supplementary Table 8**. List of proteins recovered by the RNA pull-down samples analyzed by Mass Spectrometry. | | | | | | | | | | |
| --- | --- | --- | --- | --- | --- | --- | --- | --- | --- | --- |
| prot_acc | prot_desc | prot_score | prot_mass | prot_matches | prot_matches_sig | prot_sequences | prot_sequences_sig | prot_cover | prot_pi | emPAI |
| sp\|Q15365\|PCBP1_HUMAN | Poly(rC)-binding protein 1 OS=Homo sapiens OX=9606 GN=PCBP1 PE=1 SV=2 | 117 | 37987 | 2 | 2 | 2 | 2 | 6.2 | 6.66 | 0.18 |
| sp\|P07437\|TBB5_HUMAN | Tubulin beta chain OS=Homo sapiens OX=9606 GN=TUBB PE=1 SV=2 | 112 | 50095 | 4 | 4 | 4 | 4 | 9 | 4.78 | 0.29 |
| sp\|Q15366\|PCBP2_HUMAN | Poly(rC)-binding protein 2 OS=Homo sapiens OX=9606 GN=PCBP2 PE=1 SV=1 | 99 | 38955 | 2 | 2 | 2 | 2 | 6 | 6.33 | 0.18 |
| sp\|P52597\|HNRPF_HUMAN | Heterogeneous nuclear ribonucleoprotein F OS=Homo sapiens OX=9606 GN=HNRNPF PE=1 SV=3 | 94 | 45985 | 5 | 5 | 3 | 3 | 7.7 | 5.38 | 0.32 |
| sp\|Q12906\|ILF3_HUMAN | Interleukin enhancer-binding factor 3 OS=Homo sapiens OX=9606 GN=ILF3 PE=1 SV=3 | 63 | 95678 | 3 | 3 | 2 | 2 | 2.3 | 8.86 | 0.07 |
| sp\|Q08211\|DHX9_HUMAN | ATP-dependent RNA helicase A OS=Homo sapiens OX=9606 GN=DHX9 PE=1 SV=4 | 58 | 142181 | 5 | 5 | 5 | 5 | 3.7 | 6.41 | 0.12 |
| sp\|Q99729\|ROAA_HUMAN | Heterogeneous nuclear ribonucleoprotein A/B OS=Homo sapiens OX=9606 GN=HNRNPAB PE=1 SV=2 | 42 | 36316 | 1 | 1 | 1 | 1 | 3.9 | 8.22 | 0.09 |
| sp\|P0CG38\|POTEI_HUMAN | POTE ankyrin domain family member I OS=Homo sapiens OX=9606 GN=POTEI PE=3 SV=1 | 34 | 122858 | 1 | 1 | 1 | 1 | 1.2 | 5.83 | 0.03 |
| sp\|P61978\|HNRPK_HUMAN | Heterogeneous nuclear ribonucleoprotein K OS=Homo sapiens OX=9606 GN=HNRNPK PE=1 SV=1 | 33 | 51230 | 1 | 1 | 1 | 1 | 2.2 | 5.39 | 0.06 |
| sp\|P04406\|G3P_HUMAN | Glyceraldehyde-3-phosphate dehydrogenase OS=Homo sapiens OX=9606 GN=GAPDH PE=1 SV=3 | 31 | 36201 | 1 | 1 | 1 | 1 | 2.1 | 8.57 | 0.09 |
| sp\|Q04828\|AK1C1_HUMAN | Aldo-keto reductase family 1 member C1 OS=Homo sapiens OX=9606 GN=AKR1C1 PE=1 SV=1 | 31 | 37221 | 1 | 1 | 1 | 1 | 2.5 | 8.02 | 0.09 |
| sp\|P25705\|ATPA_HUMAN | ATP synthase subunit alpha, mitochondrial OS=Homo sapiens OX=9606 GN=ATP5F1A PE=1 SV=1 | 30 | 59828 | 1 | 1 | 1 | 1 | 1.3 | 9.16 | 0.05 |
| sp\|P0C7V9\|ME15P_HUMAN | Putative methyltransferase-like protein 15P1 OS=Homo sapiens OX=9606 GN=METTL15P1 PE=5 SV=1 | 26 | 27154 | 1 | 1 | 1 | 1 | 6.4 | 5.61 | 0.12 |
| sp\|Q9NU22\|MDN1_HUMAN | Midasin OS=Homo sapiens OX=9606 GN=MDN1 PE=1 SV=2 | 26 | 638008 | 1 | 1 | 1 | 1 | 0.1 | 5.46 | 0.01 |
| sp\|Q9UN86\|G3BP2_HUMAN | Ras GTPase-activating protein-binding protein 2 OS=Homo sapiens OX=9606 GN=G3BP2 PE=1 SV=2 | 26 | 54145 | 1 | 1 | 1 | 1 | 2.3 | 5.41 | 0.06 |
| sp\|P36957\|ODO2_HUMAN | Dihydrolipoyllysine-residue succinyltransferase component of 2-oxoglutarate dehydrogenase complex, mitochondrial OS=Homo sapiens OX=9606 GN=DLST PE=1 SV=4 | 25 | 49067 | 1 | 1 | 1 | 1 | 1.8 | 9.11 | 0.07 |
| sp\|Q9Y6M1\|IF2B2_HUMAN | Insulin-like growth factor 2 mRNA-binding protein 2 OS=Homo sapiens OX=9606 GN=IGF2BP2 PE=1 SV=2 | 25 | 66195 | 1 | 1 | 1 | 1 | 2 | 8.48 | 0.05 |
| sp\|Q9H361\|PABP3_HUMAN | Polyadenylate-binding protein 3 OS=Homo sapiens OX=9606 GN=PABPC3 PE=1 SV=2 | 24 | 70215 | 1 | 1 | 1 | 1 | 1.3 | 9.68 | 0.05 |
| sp\|A8K2U0\|A2ML1_HUMAN | Alpha-2-macroglobulin-like protein 1 OS=Homo sapiens OX=9606 GN=A2ML1 PE=1 SV=3 | 23 | 162430 | 1 | 1 | 1 | 1 | 1 | 5.5 | 0.02 |
| sp\|O95573\|ACSL3_HUMAN | Long-chain-fatty-acid--CoA ligase 3 OS=Homo sapiens OX=9606 GN=ACSL3 PE=1 SV=3 | 21 | 81338 | 1 | 1 | 1 | 1 | 1.1 | 8.65 | 0.04 |
| sp\|Q9P2R7\|SUCB1_HUMAN | Succinate--CoA ligase [ADP-forming] subunit beta, mitochondrial OS=Homo sapiens OX=9606 GN=SUCLA2 PE=1 SV=3 | 18 | 50627 | 1 | 1 | 1 | 1 | 1.3 | 7.05 | 0.07 |
| sp\|P11142\|HSP7C_HUMAN | Heat shock cognate 71 kDa protein OS=Homo sapiens OX=9606 GN=HSPA8 PE=1 SV=1 | 18 | 71082 | 1 | 1 | 1 | 1 | 2.5 | 5.37 | 0.05 |
